# The senescence-associated secretory phenotype is potentiated by feedforward regulatory mechanisms involving Zscan4 and TAK1

**DOI:** 10.1038/s41467-018-04010-4

**Published:** 2018-04-30

**Authors:** Boyi Zhang, Da Fu, Qixia Xu, Xianling Cong, Chunyan Wu, Xiaoming Zhong, Yushui Ma, Zhongwei Lv, Fei Chen, Liu Han, Min Qian, Y. Eugene Chin, Eric W. -F. Lam, Paul Chiao, Yu Sun

**Affiliations:** 1 Key Laboratory of Stem Cell Biology, Institute of Health Sciences, Shanghai Institutes for Biological Sciences, Chinese Academy of Sciences and Shanghai Jiao Tong University School of Medicine, University of Chinese Academy of Sciences, 200031 Shanghai, China; 2 Central Laboratory for Medical Research, Shanghai Tenth People’s Hospital, Tongji University School of Medicine, 200072 Shanghai, China; 3 Institute of Health Sciences, Shanghai Jiao Tong University School of Medicine and Shanghai Institutes for Biological Sciences, Chinese Academy of Sciences, 200031 Shanghai, China; 4 Tissue Bank, China-Japan Union Hospital, Jilin University, 130033 Changchun, Jilin China; 5 Department of Pathology, Shanghai Pulmonary Hospital, Tongji University School of Medicine, 200433 Shanghai, China; 6 Department of Radiology, Jiangxi Provincial Tumour Hospital/Ganzhou City People’s Hospital, 330029 Nanchang, Jiangxi China; 7 Department of Nuclear Medicine, Shanghai Tenth People’s Hospital, Tongji University School of Medicine, 200072 Shanghai, China; 8 Department of Surgery and Cancer, Imperial College London, London, W12 0NN UK; 9 Department of Molecular and Cellular Oncology, The University of Texas, MD Anderson Cancer Center, Houston, TX 77030 USA; 10 Department of Medicine and VAPSHCS, University of Washington, Seattle, WA 98195 USA

## Abstract

The senescence-associated secretory phenotype (SASP) can be provoked by side effects of therapeutic agents, fueling advanced complications including cancer resistance. However, the intracellular signal network supporting initiation and development of the SASP driven by treatment-induced damage remains unclear. Here we report that the transcription factor Zscan4 is elevated for expression by an ATM-TRAF6-TAK1 axis during the acute DNA damage response and enables a long term SASP in human stromal cells. Further, TAK1 activates p38 and PI3K/Akt/mTOR to support the persistent SASP signaling. As TAK1 is implicated in dual feedforward mechanisms to orchestrate the SASP development, pharmacologically targeting TAK1 deprives cancer cells of resistance acquired from treatment-damaged stromal cells in vitro and substantially promotes tumour regression in vivo. Together, our study reveals a novel network that links functionally critical molecules associated with the SASP development in therapeutic settings, thus opening new avenues to improve clinical outcomes and advance precision medicine.

## Abstract (web summary)

In cancer the side effects of therapeutic agents can provoke senescence-associated secretory phenotype (SASP), which can drive cancer resistance. During the DNA damage response, transcription factor Zscan4 expression is elevated by an ATM-TRAF6-TAK1 axis leading to long term SASP in human stromal cells.

## Introduction

Anticancer strategies including chemotherapy, ionizing radiation (IR), and targeted therapy are initially effective in debulking the tumour mass by producing significant responses^1^. However, most malignancies resist subsequent treatments, and frequently progress to advanced stages with lethal phenotypes^2^. Despite promising advances supported by both cancer research and pharmaceutical pipelines, therapy resistance is hitherto a major barrier to clinical success. With the mounting arsenal of therapeutic agents and high-throughput screening technologies, there are now unprecedented opportunities to circumvent drug resistance via establishment of predictive biomarkers to enable patient stratification^3^. Diverse mechanisms of cancer resistance are identified and can be generalized into two major categories: de novo and acquired^4^. In contrast to de novo resistance, which pre-exists as a relatively static issue to tackle, acquired resistance arises unpredictably upon treatments and poses a daunting challenge to clinical management^5, 6^. Although these mechanisms are clearly operative, targeting malignant cells in the tumour foci can rarely cure patients, implying the presence of extrinsic forces that exert seemingly cryptic, but indeed substantial and decisive, if any, effects to cause disease resilience.

Tumour development involves the co-evolution of transformed cells and the tumour microenvironment (TME), the latter frequently dominating therapeutic response^2^. The TME comprises many non-cancerous cell types including fibroblasts, endothelial cells, and infiltrating lymphocytes. The specific mechanisms through which the TME promotes malignant progression are, however, markedly 'co-opted' in the context of therapy resistance, with soluble factors, structural components, and even metabolic products jointly altering clinical indexes^7^. Importantly, we and others have identified the senescence-associated secretory phenotype (SASP)^8–10^, a distinct secretory phenotype of senescent cells including those generated by the side effect of chemotherapy and radiation, can unintendedly, but significantly promote drug resistance, local inflammation, and tumour metastasis by enforcing the secretion of cells that survive treatments^11, 12^. As a full senescence response is indeed not required for the SASP, a phenomenon mainly observed upon genotoxic stimuli, the DNA damage secretory program (DDSP) is probably more appropriate to depict the mechanism responsible for a typical SASP^11, 13–15^. Expression of soluble factors in the full SASP spectrum not only induces an epithelial to mesenchymal transition (EMT), but also creates an immunosuppressive milieu, thereby driving tumorigenesis and shaping tumour resistance against multiple types of anticancer agents^16–18^.

In contrast to the telomere erosion-associated SASP as a chronic event, the therapy-induced SASP usually arises 5 to 8 days after the onset of treatment and develops from an acute stress associated phenotype (ASAP), the relatively rapid cellular response to cytotoxic agents before the appearance of senescence markers^19, 20^. Thus, the ASAP represents a spatiotemporally specific response, through which cells sense environmental stress and initiate rapidly a self-protective program in adverse conditions. Significant collateral macromolecular damages, specifically DNA strand breaks, to the non-cancerous stroma represent a primary force to elicit the in vivo SASP at the residual foci of cancer survivors and generate deleterious effects on tissue homeostasis^21^. Previous studies revealed the functional role of several molecules in SASP development, such as γH2AX and macroH2A1 histone variants, NF-κB and C/EBP-β transcription factors, ATM, p38, mTOR and JAK1/2 kinases, TGF-β and IL-1α chemokines, and SIRT3/5 mitochondrial sirtuins^14, 15, 22–27^. However, the number of functionally crucial factors that can be specifically exploited to pharmacologically target the SASP is limited, except a small handful of intracellular kinases including p38 and mTOR, which mainly regulate the SASP via NF-κB transcription^28, 29^.

Despite research advances in senescence biology, whether there are additional key molecules favoring development of the acute ASAP and supporting its transition toward the chronic SASP, remains an intriguing topic. By comparative transcriptomics analysis of human stromal cells exposed to anticancer treatments, we disclosed that the Zinc Finger and Scan Domain Containing 4 (Zscan4), a zinc finger transcription factor, is essential for mediating DNA damage response (DDR) signals to forge the SASP development after the ASAP burst induced by genotoxic stimuli. There are sequential events that relay DNA damage signals, including ATM-TRAF6-TAK1 axis formation, Zscan4 positive feedback, p38 activation, PI3K/Akt/mTOR engagement, IL-1α-mediated TAK1 consolidation, and the SASP expression in stromal cells. Importantly, targeting TAK1 by a small molecule inhibitor dampens the pro-tumourigenic activities of the SASP, especially cancer resistance acquired from the treatment-damaged stroma. Taken together, identifying the functional implication of these molecules in a TME landscape provides new options to improve clinical outcomes of cancer patients by minimizing the TME-conferred therapeutic resistance in contemporary medicine.

## Results

### Zscan4 expression is enhanced in DNA-damaged stromal cells

**Fig. 1:**
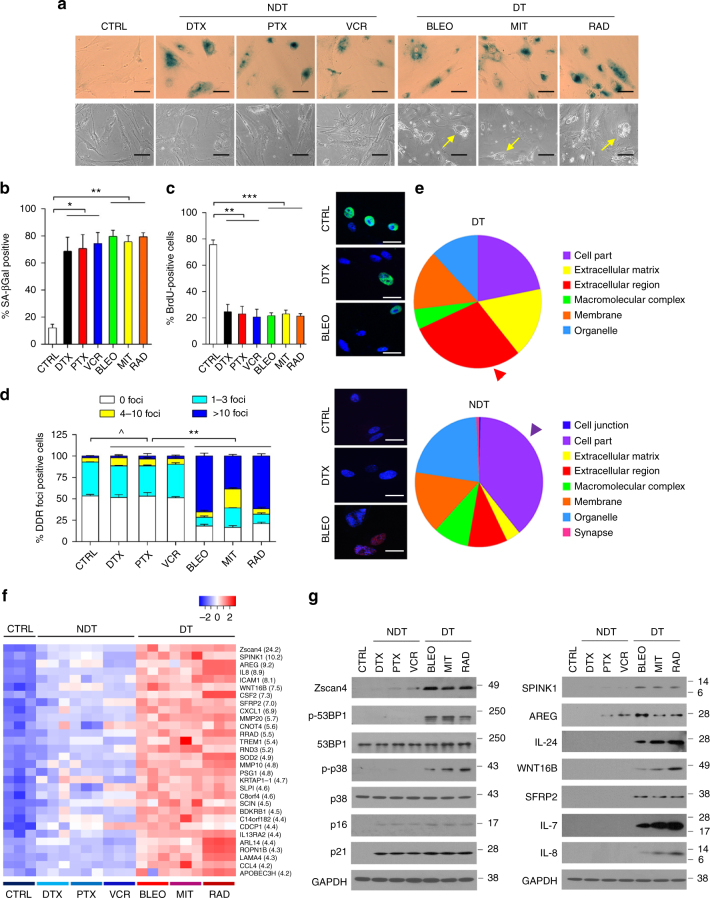
Genotoxic treatments cause distinct gene expression in human stromal cells with remarkable Zscan4 upregulation. **a** Representative images of human stromal cell (PSC27) senescence, developed 7–10 d after treatment by two different classes of anticancer agents. CTRL control, NDT non DNA-damaging treatment, DTX docetaxel, PTX paclitaxel, VCR vincristine, DT DNA-damaging treatment, BLEO bleomycin, MIT mitoxantrone, and RAD radiation. Upper panel, SA-β-Gal staining; lower panel, phase contrast. Yellow arrows, cytoplasmic vacuoles. Scale bars, 15 μm. **b** SA-β-Gal staining statistics of cells in the individual groups of (**a**). Data obtained from counting of at least 200 cells per independent experiment, and analyzed using Student’s *t*-test. **c** BrdU incorporation profiles of cells in the individual groups of (**a**), with representative images provided on the right (CTRL/DTX/BLEO shown for example; green, BrdU; blue, DAPI). At least 200 cells counted per independent assay. Scale bars, 15 µm. Data analyzed using Student’s *t*-test. **d** Quantification of DNA damage foci (DDF). The number of DDF per cell falls into each of the 0, 1–3, 4–10, and >10 counting categories. Right, representative images (CTRL/DTX/BLEO displayed for example; red, γH2AX; blue, DAPI). At least 200 cells counted per sample. Scale bars, 15 µm. Data analyzed using two-way ANOVA. **e** GO analysis (cellular component, top 100 genes) of cells treated by DT and NDT agents, respectively. Color annotations shown on the right of Panther pie charts. **f** Heatmap depiction of top 30 human genes significantly upregulated in stromal cells after treatment by DT agents, with expression profiles generated by NDT agents shown side-by-side for parallel comparison. Numeric values in the bracket per gene refer to the expression fold change of the DT groups against the control. **g** Immunoblot analysis of the expression or activation of proteins including Zscan4, 53BP1, p38, p16, p21, and a subset of canonical SASP factors. Unless noted, data in all bar plots are shown as mean ± SD and representative of 3 biological replicates. * *P* < 0.05, ** *P* < 0.01, *** *P* < 0.001, ^ *P* > 0.05

To characterize the genome-wide expression of human stromal cells, we employed a diploid primary normal human prostate stromal line PSC27, and two distinct subtypes of anticancer therapeutics, namely non-DNA-damaging treatment (NDT) versus DNA-damaging treatment (DT) (Fig. 1a). Notably, all treatments caused evident cellular senescence, as indicated by positive SA-β-Gal staining and remarkable morphological alterations (Fig. 1a, b). However, these agents markedly differed in several aspects including vacuole development in the cytoplasm, DNA synthesis rate, and DNA damage severity (Fig. 1a, c, d). Microarray analysis indicated that the DT group caused enhanced expression of genes encoding extracellular matrix (ECM) components and soluble factors, in relative to the NDT group. This is supported by data from analysis of gene ontology (GO)-cellular component (CC), which specifies the location of a protein linked with certain function (Fig. 1e and Supplementary Fig. 1a). The number of genes concordantly upregulated by these two classes counts for 12.5% of the overall gene list of the DT, and 47.9% of the NDT (>3 fold change in ease case), respectively (Supplementary Fig. 1b). Converged with the parallel data from profiling of downregulated genes (24.3% and 67.3%, respectively, Supplementary Fig. 1c), the results were strongly indicative of differentially altered genome-wide expression pattern of human stromal cells by functionally distinct agents.

Beyond diverse extracellular proteins, Zscan4, a transcription factor correlated with telomere elongation and genomic stability in embryonic stem cells^30^, emerged as one of the major intracellular molecules that differentiate the DT effects from the NDT influence (Fig. 1f). Further, Zscan4 expression appeared more readily inducible by DT agents in stromal cells rather than cancer epithelial cells of the same organ of origin, the prostate (Supplementary Fig. 1d), suggesting a cell lineage dependency.

Hierarchical clustering revealed the outstanding similarity and intimate correlation of gene expression profile generated by the DT group, in contrast to that induced by NDT agents (Supplementary Fig. 1e,f). Upon genotoxic stress, Zscan4 protein was detectable throughout the cytoplasm and nucleus, with signals more prominent in the nuclear region, indicating a potential role in DDR-associated activities (Supplementary Fig. 1g,h). We also noticed p38, a stress-inducible mitogen-activated protein kinase (MAPK), that regulates the SASP in normal human fibroblasts^28^, was specifically activated by the DT rather than the NDT drugs (Fig. 1g). Immunoblots indicated the differential expression of a subset of canonical SASP factors such as SPINK1 and AREG^11, 29^, further demarcating the distinct functionalities (Fig. 1g). As supporting evidence, we observed a similar DNA damage-forged phenotype in HBF1203 (Supplementary Fig. 1i), a stromal cell line isolated from human breast tissue^11^. Together, the concomitant expression of Zscan4 and hallmark SASP effectors in stromal cells imply there might be specific mechanisms functionally connecting these molecules in a DNA damage context.

### Stromal Zscan4 expression in chemo-treated cancer patients

**Fig. 2:**
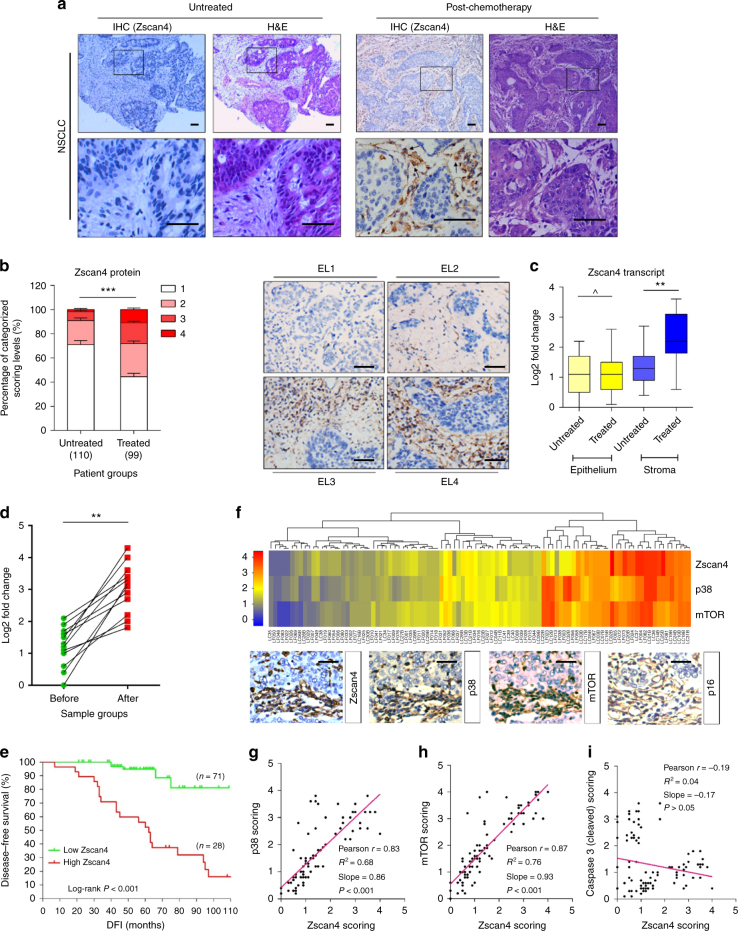
Zscan4 is expressed in human lung tumour stroma after chemotherapy and correlated with poor survival statistics. **a** Histological assessment of Zscan4 in primary tumour tissues of NSCLC patients. Arrows in the IHC image, intensive nuclear signals of Zscan4 in stroma. Scale bars, 50 µm. Cisplatin was used as a chemotherapeutic agent for the treated patient. **b** Pathological appraisal of stromal Zscan4 expression in NSCLC samples. Patients were individually assigned into 4 categories per IHC staining intensity of the stroma. 1, negative; 2, weak; 3, moderate; 4, strong expression. Left, statistical comparison. Right, representative images. ES, expression level. Scale bars, 50 µm. Data analyzed by two-way ANOVA. **c** Boxplot summary of Zscan4 transcript expression by qRT-PCR analysis upon laser capture microdissection of cells from tumour and stroma, individually from samples of 10 randomly selected patients. **d** Zscan4 expression at transcription level in stromal cells collected before versus after chemotherapy of the same individuals. **e** Kaplan–Meier analysis of NSCLC patient. Disease free survival (DFS) stratified according to Zscan4 expression. DFS represents the length (months) of period calculated from the date of NSCLC diagnosis to the point of first time disease relapse. Data compared with log-rank (Mantel–Cox) test. **f** Pathological correlation between Zscan4, p38, and mTOR in the stroma of NSCLC patients post treatment. Columns represent individual patients, rows different molecules. Totally 99 patients treated by chemotherapy were analyzed. Bottom, representative IHC images of Zscan4, p38 (T-180), and mTOR (S-2448), respectively. *P* < 0.0001 for score-score matching by two-way ANOVA. **g** Statistical correlation between Zscan4 and p38 scores in the 99 tumours with matching protein expression data. **h** Statistical correlation between Zscan4 and mTOR scores in the same group of tumours described in **f**. **i** Statistical correlation between Zscan4 and caspase 3 (cleaved) scores in the same group of tumours described in **f**. **j** Data in all bar plots are shown as mean ± SD and representative of 3 biological replicates. Data in **c**, **d** were analyzed using Student’s *t*-test.* *P* < 0.05, ** *P* < 0.01, *** *P* < 0.001, ^ *P* > 0.05

We next sought to determine whether a similar response can be observed in vivo. As the SASP is a cell non-autonomous phenotype developed upon macromolecular damage independent of tissue or organ types^21^, we first assessed tumour samples acquired from a cohort of non-small-cell lung cancer (NSCLC) patients. Although Zscan4 protein level in both tumour and stroma of patients without a pre-surgery chemotherapy history was largely negligible, we found striking Zscan4 expression in tumour foci of NSCLC patients who underwent chemotherapeutic regimens that mainly involved genotoxic agents, with the protein substantially localized in the stroma, in sharp contrast to the surrounding epithelium (Fig. 2a).

To further delineate Zscan4 expression in tissue, we adopted a pre-determined pathological evaluation system that allows qualitative interpretation of a target protein expression according to its IHC signal intensity. Relative to the tumours of untreated patients, samples of patients who received chemotherapy displayed higher Zscan4 levels (Fig. 2b). Upon separation of epithelium and stroma from tumour tissues by laser capture microdissection (LCM), we observed considerably increased Zscan4 transcript in the stroma of post-treated patients (Fig. 2c). Specifically, we interrogated whether Zscan4 expression increased even in the same patient after chemotherapy. To address this, we investigated a subgroup of patients whose pre- and post-treatment samples were both accessible, and found consistently enhanced Zscan4 signals in their post-treatment tissues (Fig. 2d). More importantly, Kaplan–Meier analysis of NSCLC patients indicated that enhanced stromal Zscan4 expression significantly correlates with poor clinical outcome in the treated cohort, with lower Zscan4 expression favoring longer survival (Fig. 2e).

p38 is a key mediator of the SASP by both regulating NF-kB activity and stabilizing SASP effector mRNA stability post-transcriptionally in senescent fibroblasts^27, 28^. Another intercellular kinase, mTOR, governs the SASP development through promoting IL-1α protein synthesis and controlling MAPKAPK2 kinase translation^29, 31^. However, the biological relationship between p38, mTOR, and Zscan4 in the SASP setting remains unclear. We pathologically analyzed p38 and mTOR activation via a similar appraisal strategy in a case-to-case manner, and found synchronized changes in Zscan4 expression and p38/mTOR activation in the tumour stroma of NSCLC patients (Fig. 2f). Further, statistical data suggested close correlations between Zscan4 and p38/mTOR, but not a SASP-unrelated factor caspase 3 (Fig. 2g–i).

Of note, both the stroma-intensive expression of Zscan4 in the post-treatment patients and significant correlations between Zscan4 and p38/mTOR were validated through pathological assessment of human breast cancer (BCa) patient samples (Supplementary Fig. 2a–i). Altogether, clinical data demonstrated that Zscan4 is preferentially expressed in solid tumour stroma, and raised the possibility that a biological mechanism links Zscan4 expression with central mediators of the SASP,

### Zscan4 functionally changes the SASP but not senescence

**Fig. 3:**
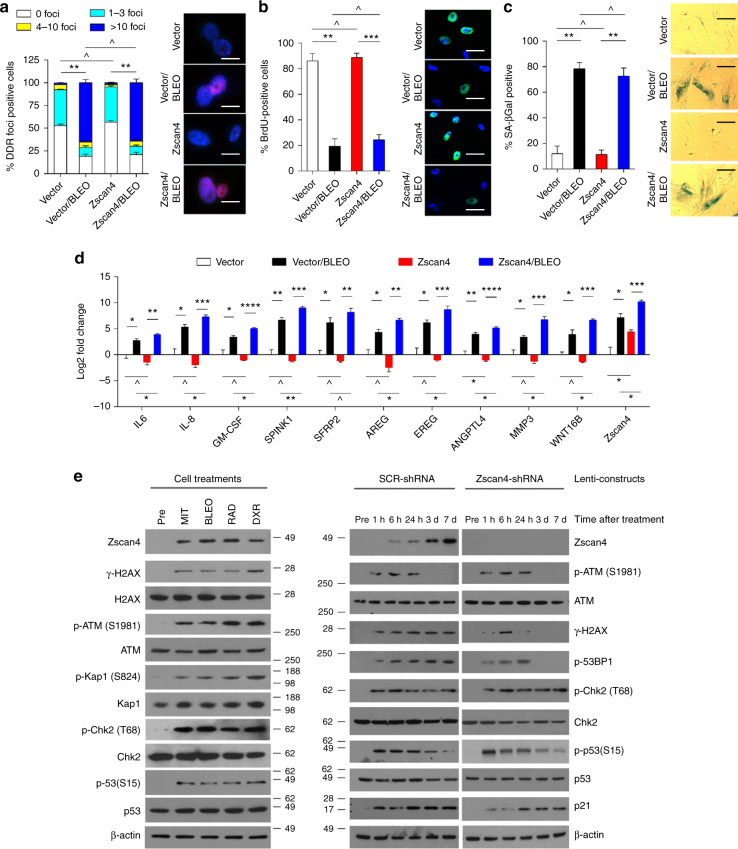
Zscan4 overexpression does not change stress-induced cell phenotypes but alters SASP expression and DDR signaling. **a** Statistics of DDR foci in PSC27 stable sublines established via lentiviral infection and treated by bleomycin (50 µg/ml). The number of cells displaying each category of DDR foci was counted and converted into percentage per cell population. Right, representative images (red, γH2AX; blue, DAPI). At least 200 cells counted per sample. Scale bars, 10 µm. Data analyzed by two-way ANOVA. **b** DNA synthesis evaluation by BrdU incorporation of cell sublines assayed in **a**, with BrdU-positive cells calculated in percentage. Right, representative images (green, BrdU; blue, DAPI). At least 200 cells counted per sample. Scale bars, 15 µm. Data analyzed by Student’s *t*-test. **c** Cellular senescence assessment by SA-β-Gal staining of above cell sublines, with positively stained cells shown in percentage. Right, representative images. At least 200 cells counted per sample. Scale bars, 20 µm. Data analyzed by Student’s *t*-test. **d** Transcript expression of canonical SASP factors in stromal sublines treated in **a**, with values normalized to the vector control per factor. Data analyzed by Student’s *t*-test. **e** Left, immunoblot analysis of inducible expression of Zscan4 and activation of DDR factors including H2AX, ATM, Kap1, Chk2, and p53 in primary PSC27 cells treated by chemotherapeutic agents. DXR, doxorubicin, a supplementary genotoxic control; Right, cells were lentivirally transduced with scramble-shRNA (SCR-shRNA) or gene-specific shRNA for Zscan4 (Zscan4-shRNA), and treated by bleomycin. Cell lysates were collected at the individual time points post treatment for immunoblotting against Zscan4 expression and DDR factor activation. Data in all bar plots are shown as mean ± SD and representative of 3 representative biological replicates. *P < 0.05, **P < 0.01, ***P < 0.001, ****P < 0.0001, ^*P* > 0.05

The prominent concordance between distinct Zscan4 expression and key SASP regulator activation observed in cancer patient specimens prompted us to further explore the functional implication of Zscan4 in damaged stromal cells. First, we examined the effects of Zscan4 overexpression or elimination (PSC27-Zscan4 and PSC27-Zscan4-KD, respectively). Neither forcibly increased nor basically depleted expression of Zscan4 changed the genotoxicity-induced patterns characterized by increased DNA damage foci (DDF) number, reduced DNA synthesis, enhanced cellular senescence, or decreased colony formation (Fig. 3a–c and Supplementary Fig. 3a–e). However, exogenously transduced Zscan4 suppressed the expression of most SASP factors (including but not limited to IL-6, IL-8, GM-CSF, SPINK1, SFRP2, AREG, EREG, ANGPTL4, MMP3, and WNT16B), although moderately. In contrast, expression of these canonical SASP factors was dramatically enhanced after DNA damage to PSC27-Zscan4 cells, with the fold change per factor even higher than that observed in damaged control cells (PSC27-Vector) (Fig. 3d).

To gain further insights into the role of Zscan4 during the SASP development, we evaluated the consequence of Zscan4 elimination on the SASP expression. Interestingly, PSC27-Zscan4-KD cells displayed slightly increased expression of the SASP factors at basal level, albeit not significantly (Supplementary Fig. 3f). As compared with control cells (PSC27-SCR-shRNA), Zscan4 knockdown caused essentially diminished expression of most SASP factors upon DNA damage. Thus, overexpressed Zscan4 seems to restrain the basal SASP level, but remarkably amplifies diverse SASP canonical factor expression when stromal cells are exposed to DNA damage.

Next, we sought to determine the effect of Zscan4 deficiency on cellular responses to DNA damage, by mainly dissecting DDR signaling. We found that Zscan4 expression occurs almost simultaneously with activation of the DDR machinery and its downstream targets including phosphorylated histone H2AX (γ-H2AX), ATM, Kap, Chk2, and p53 (Fig. 3e left). Upon DNA damage, Zscan4 was readily upregulated, although in a time-dependent manner, with the protein detectable as early as 6 h post treatment. This was generally behind the time point when most of central DDR factors including p-ATM, γ-H2AX, p-53BP1, and their substrates appeared in cell lysates. However, in the absence of Zscan4, activation of both H2AX and 53BP1 was transient and did not last longer than 24 h (Fig. 3e right). Despite such a short-term response, DDR downstream molecular events including phosphorylation of Chk2, p53, and upregulation of p21, persisted throughout the entire SASP development period as we extended the experiment to 7d after treatment. The data indicate that the genotoxicity-induced cellular senescence is basically intact, with checkpoint arrest and downstream reaction occurring in a largely unaffected cascade. Conversely, we analyzed the response of DDR-associated factors in PSC27-Zscan4 cells, and found elongated duration of ATM phosphorylation with strengthened H2AX/53BP1 activation in damaged cells (Supplementary Fig. 3g). As observed earlier, after synthesized in stromal cells, a large amount of Zscan4 translocated from the cytoplasm to the nucleus, where the DDR signals arised after DNA damage (Supplementary Fig. 1g,h). The results suggest that Zscan4 is pivotal for the maintenance of a typical DDR chain and may have the capacity to mediate the transition of an acute response (ASAP) to a chronic event (SASP) by functionally participating in early DDR signaling activities within the nucleus of damaged stromal cells.

### Zscan4 expression is acutely induced by ATM-TRAF6-TAK1 axis

**Fig. 4:**
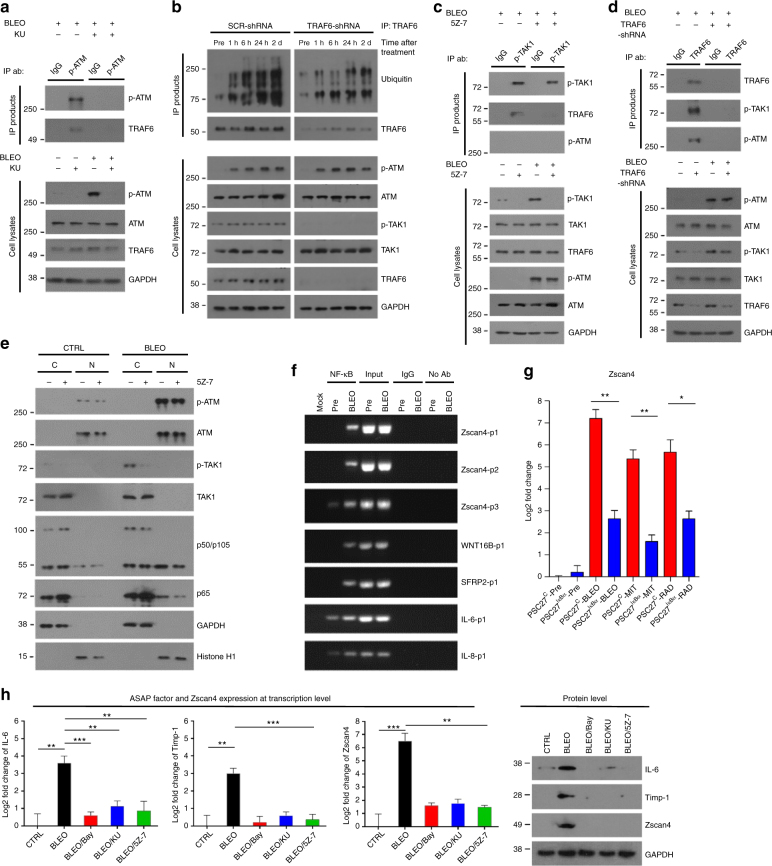
Zscan4 expression is induced by DNA damage and regulated by the ATM/TRAF6/TAK1/p65 signaling axis. **a** PSC27 cells were treated by bleomycin (50 μg/ml) with or without KU55933 (KU, 10 μM) in culture. Cell lysates were immunoprecipitated with anti-p-ATM, with the immunoprecipitates (IPs) analyzed with anti-p-ATM and anti-TRAF6, respectively. **b** PSC27 cells lentivirally infected with scramble or TRAF6-specific shRNAs were treated with bleomycin, with lysates collected at indicated time points and subject to IP and immunoblot assays. **c** Anti-TAK1-based IP of PSC27 cells treated with bleomycin in the presence or absence of 5Z-7 (500 nM), followed by immunoblot analysis. **d** Primary PSC27 cells or those stably expressing shRNA to TRAF6 were treated with bleomycin, with IPs pulled down by anti-TRAF6 and examined by immunoblots. **e** Cytoplasmic and nuclear protein samples from control and bleomycin-treated PSC27 cells were analyzed for TAK1 activation and NF-κB nuclear translocation. GAPDH and Histone H1, cytoplasmic and nuclear loading controls, respectively. **f** Chromatin immunoprecipitation (ChIP) was performed to identify potential NF-κB binding sites in Zscan4 proximal promoter. Zscan4-p1/p2/p3 denotes 3 representative genomic sites in promoter region, with known NF-κB binding sites from WNT16B, SFRP2, IL-6, and IL-8 selected as positive controls. **g** Zscan4 transcript expression in PSC27 cells stably expressing an NF-κB-null mutant and treated by bleomycin, mitoxantrone or radiation. Signals normalized to untreated cells. **h** Expression profiling of typical ASAP factors (IL-6/Timp-1) and Zscan4 in stromal cells exposed to bleomycin. Left (histograms), cells were pretreated with inhibitors of NF-κB, ATM, or TAK1 (Bay, KU or 5Z-7, respectively) before addition of bleomycin, with transcripts collected for analysis 24 h after genotoxic treatment (data normalized to untreated sample per factor set). Right (immunoblots), protein level assessment of IL-6, Timp-1, and Zscan4 24 h after cell exposure to bleomycin. GAPDH, loading control. Data in all bar plots are shown as mean ± SD and representative of 3 biological replicates. BLEO bleomycin, KU KU55933, 5Z-7 5*Z*-7-oxozeaenol, Bay Bay 11–7082. Data in **g**, **h** were analyzed by Student’s *t*-test. *P < 0.0*5*, **P < 0.01, ***P < 0.001, ^*P* > 0.05

Functional data of Zscan4 prompted us to further investigate Zscan4 expression mechanism in stromal cells damaged by genotoxicity. It was reported that DNA damage induces rapid ATM activation and subsequent translocation from nucleus to cytoplasm, eventually causing downstream changes including activation of the NF-κB complex^32^. Thus, we first analyzed PSC27 cell lysates by phosphorylated ATM (p-ATM)-based immunoprecipitation (IP) shortly after bleomycin treatment, and found a physical interaction between activated ATM and TRAF6, albeit diminished by an ATM inhibitor KU55933 (Fig. 4a). As binding of ATM to TRAF6 triggers TRAF6-mediated poly-ubiquitination in cancer cells, a step responsible for subsequent TAK1 activation in their cytoplasm^32^, we asked whether these findings are reproducible in stroma cells. To address this, we first examined the cell lysates by IP with anti-TRAF6 after DNA damage, and noticed rapidly augmented TRAF6 auto-ubiquitination, an indicator of its ubiquitin ligase activity in damaged cells (Fig. 4b). We then performed phosphorylated TAK1 (p-TAK1)-mediated IP, and found physical association between activated TAK1 and TRAF6, a process that occurred shortly after DNA damage but subject to suppression by a TAK1 inhibitor, 5*Z*-7-oxozeaenol (hereafter as 5Z-7) (Fig. 4c). In contrast, there was essentially no interaction between TAK1 and ATM, although both molecules were activated shortly after DNA damage, indicating the specificity of TAK1-TRAF6 interplay (Fig. 4c). As supporting evidence, data from reverse IP with an antibody to TRAF6 showed that TRAF6 can interact with both ATM and TAK1 in their activated state, implying the role of TRAF6 as a central factor that transmits ATM-relayed damage signals to TAK1 (Fig. 4d). As TRAF6 knockdown abolished activation of TAK1, but not ATM in damaged cells, the function of TRAF6 in acute signaling network of the ASAP was further substantiated (Fig. 4b). We further asked if TAK1 indirectly activated by upstream DDR signaling is responsible for NF-κB activation, a process reported to be mediated by mono-ubiquitination of the IκB kinase subunit γ (IKKγ) in the cytoplasm^32^. Upon separation of nuclear contents from cytoplasmic extracts, we noticed that TAK1 phosphorylation was linked with nuclear recruitment of p50 and p65, the major subunits of NF-κB complex (Fig. 4e). In the presence of 5Z-7, however, both TAK1 phosphorylation and p50/p65 nuclear translocation were weakened, confirming NF-κB activation as a downstream event of TAK1-mediated signaling (Fig. 4e). Alternatively, we analyzed the cytoplasmic and nuclear fractions of the breast stromal cell line HBF1203 after DNA damage, and the data essentially reproduced the changes observed in PSC27 (Supplementary Fig. 4a–d).

We next reasoned whether the NF-κB complex, a key transcription machinery of the transcriptome-wide SASP expression^33^, drives acute Zscan4 expression in damaged stromal cells. Bioinformatics analysis disclosed 6 putative NF-κB binding sequences in the proximal promoter region of Zscan4 (Supplementary Fig. 4e). Promoter characterization through sequential cloning followed by reporter assays with tumour necrosis factor (TNF-α) and genotoxic treatments, well-known NF-κB activators, revealed 4 bona fide NF-κB binding motifs in a 4000 bp region immediately upstream of Zscan4 transcription start site (TSS) (Supplementary Fig. 4f,g). The results were further validated by chromatin IP with stromal cell lysates (Fig. 4f). We previously generated stromal cells stably expressing an IκBα mutant that blocks NF-κB nuclear translocation and attenuates its transcriptional activity in stressful settings including those associated with DNA damage (PSC27^IκBα^)^11^. Compared with the wild type control, PSC27^IκBα^ cells with NF-κB deficiency had significantly lower induction of Zscan4 upon treatment by genotoxic agents (Fig. 4g). Thus, our data consistently demonstrated that Zscan4 is an authentic and direct NF-κB target in DNA-damaged stromal cells.

In addition, we found targeting the NF-κB complex with a specific inhibitor (Bay 11–7082) not only minimized the SASP (Supplementary Fig. 4h) but also abolished the ASAP, as evidenced by the diminished expression of IL-6 and Timp-1, two typical ASAP factors reported previously^19^ (Fig. 4h). Compared with NF-κB inhibition, pharmacological targeting ATM or TAK1 generated similar impacts on both ASAP formation and Zscan4 induction (Fig. 4h). These findings, combined with our earlier data supporting Zscan4 as a critical molecule in regulating the SASP expression (Fig. 3d), together implied that once upregulated via a ATM-TRAF6-TAK1 axis in the acute DDR response, Zscan4 functionally feeds back by favoring the formation of a feedforward mechanism that further amplifies the SASP phenotype, thereby potentiating a chronic SASP development in the subsequent time period.

### TAK1 mediates p38 signaling but not DDR

**Fig. 5:**
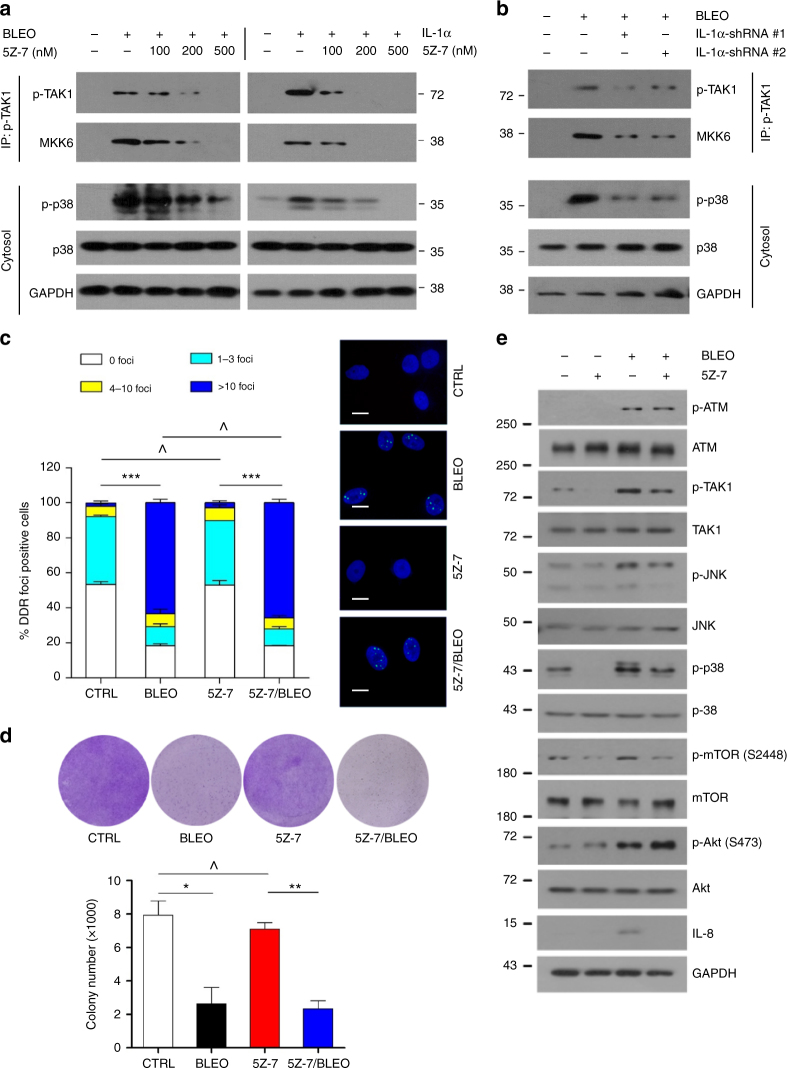
TAK1 activation engages p38 but its inhibition does not influence DDR signaling. **a** PSC27 cells were treated with bleomycin with or without increasing concentrations of the TAK1 inhibitor 5Z-7. Cell lysates were IPed with anti-p-TAK1, and subject to in vitro kinase assay with MKK6 as a substrate. Activation of kinase p38 was analyzed, with GAPDH as a loading control. Alternatively, IL-1α (20 ng/ml) was used to stimulate cells with or without 5Z-7, with the lysates subsequently analyzed. **b** IL-1α was eliminated from PSC27 cells by shRNAs. TAK1/MKK6 interaction and p38 activation in the cytosol were assessed by IP in the same conditions of **a**. **c** PSC27 cells were treated by bleomycin, or 5Z-7, or both, and subject to immunofluorescence (IF) staining of γ-H2AX. DNA damage extent in stromal cells was depicted as DDR statistics by counting the number of DDR foci per cell. Right, representative images of IF staining. Green, γ-H2AX; Blue, DAPI. Scale bars, 10 μm. Data analyzed by two-way ANOVA. **d** Top, representative images for clonogenic assay. PSC27 cells were treated by bleomycin alone for 6 h then released until 7d later, or treated by 5Z-7 alone continuously for 7d, or treated by both agents for individual time length in culture, after which clonogenic staining was performed. Bottom, statistics of stromal cell clonogenic growth. Data analyzed by Student’s *t*-test. **e** PSC27 cells were treated with bleomycin and/or 5Z-7, and cell lysates were collected 7d after drug treatment for immunoblot analysis. JNK1 phosphorylation and IL-8 expression were used to probe TAK1 activation and SASP development, respectively. Activation of mTOR and Akt were assayed with the same set of lysates, as well. Data in all bar plots are shown as mean ± SD. All data are representative of 3 biological replicates. * *P* < 0.05, ** *P* < 0.01, *** *P* < 0.001

Next, we interrogated whether and how the acute DDR signals can activate multiple factors essential for the SASP development. As TAK1 can functionally activate p38 in multiple biological events including local inflammation and tissue homeostasis^34^, we speculated that TAK1 may be involved in cellular responses responsible for the late stage progression of the SASP. First, we analyzed the activity of TAK1 in DNA-damaged PSC27 cells in the presence or absence of 5Z-7, a resorcylic acid lactone that inhibits the kinase activity of TAK1 with high potency and specificity^35^. Data from IP and in vitro kinase assay indicated that DNA damage prominently enhanced catalytic activity of TAK1 in PSC27 cells (Fig. 5a upper left). However, increasing concentrations of 5Z-7 caused progressive reduction of such an interaction, the latter essentially abrogated by 5Z-7 raised at 500 nM in culture. Analysis of cell lysates revealed p38 phosphorylation, which largely paralleled TAK1 activation in stromal cells (Fig. 5a lower left). As IL-1α is an upstream inducer of TAK1 in mammalian cells, we posited that stromal TAK1 can also be activated by the proinflammatory cytokine IL-1α, another hallmark SASP effector that once produced can in turn further consolidate the SASP phenotype^29, 36^. Thus, we treated PSC27 cells with recombinant IL-1α, and found remarkably enhanced TAK1/MKK6 interaction and p38 activation, changes that resembled the pattern observed in the case of bleomycin treatment (Fig. 5a right). To confirm the finding, we depleted PSC27 cells of IL-1α via shRNA before exposing them to DNA damage. Interestingly, IL-1α elimination substantially abolished the activation of TAK1 and its downstream target p38 in PSC27 cells after bleomycin treatment (Fig. 5b), suggesting that TAK1 activity is subject to IL-1α regulation in genotoxic settings. Of note, TAK1 inhibition did not alleviate DNA damage extent, nor did it affect the capacity of colony formation by stromal cells (Fig. 5c, d).

To exclude potential off-target effects of bleomycin, we treated cells with alternative genotoxic agents including mitoxantrone (MIT, a type II topoisomerase inhibitor) and satraplatin (SAT, a platinum analogue), which target DNA with different mechanisms. As a result, we observed similar patterns when profiling DNA damage in PSC27 cells (Supplementary Fig. 5a,b), confirming that DNA integrity is subject to the damage caused by genotoxic insults, whereas TAK1 inhibition has no influence. Further, TAK1 deficiency neither changed the overall cell proliferative potential of PSC27 cells, nor prevented the appearance of genotoxicity-induced cellular senescence (Supplementary Fig. 5c,d).

In addition, we asked if TAK1 inhibition changes the phosphorylation status of ATM after genotoxic treatment, another perspective to analyze its potential actions on DDR signaling. Immunoblots indicated that DNA damage-induced overall activation of ATM was not subject to TAK1 inhibition, although minimized activation of JNK and p38 was observed (Fig. 5e). Moreover, expression of IL-8, a hallmark index of the SASP, was considerably abolished upon TAK1 inhibition even when DNA lesions persisted. Thus, our results suggested that TAK1 is indispensable for the SASP development in damaged cells. Further, we noticed 5Z-7-mediated TAK1 suppression led to reduced mTOR but seemingly enhanced Akt phosphorylation, although both kinases were activated in DNA-damaged stromal cells (Fig. 5e). Specifically, augmented Akt activation in TAK1-inhibited cells implies that DDR signals may be transmitted through a TAK1-p38-PI3K/Akt-mTOR axis in the cytoplasm, wherein the negative feedback loop between mTOR and Akt is functionally engaged, a modality we recently observed in the rapamycin-restrained SASP^29^. Either TAK1 inhibition by 5Z-7 or mTOR suppression by rapamycin can further enhance Akt activation in damaged cells, suggesting TAK1 and mTOR may be connected via the same signaling axis. Nevertheless, the landscape of how there molecules are connected in the context of a long term SASP remains unexplored.

### mTOR is essential for SASP but downstream of TAK1 and p38

**Fig. 6:**
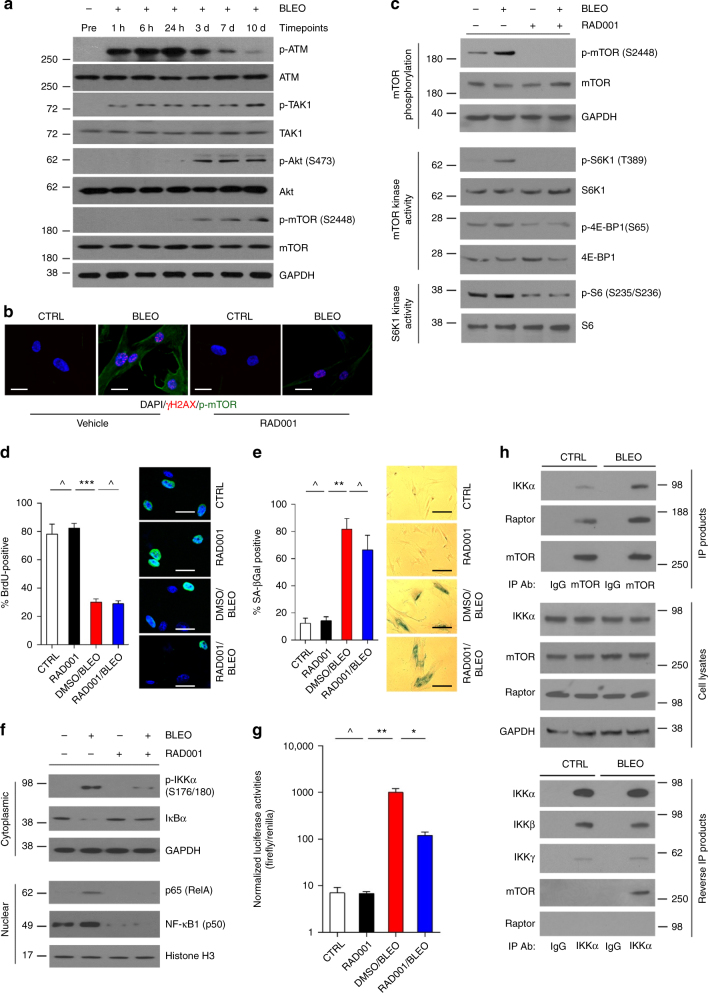
mTOR is activated and interacts with the IKK complex upon genotoxic treatment. **a** Immunoblot analysis of ATM, TAK1, Akt and mTOR activation at different time points after bleomycin treatment of PSC27 cells. Total proteins of these factors and GAPDH were loading controls. **b** Representative IF images for mTOR phosphorylation (S2448) in PSC27 cells treated by bleomycin with or without RAD001 (50 nM) (blue, DAPI; red, γH2AX; green, p-mTOR). Cells were stained 7d after bleomycin treatment. Scale bars, 15 μm. **c** Immunoblot analysis of mTOR activation and downstream substrate (S6K1/4E-BP1) phosphorylation in cells treated by bleomycin with or without RAD001. Lysates were collected 7d after initiation of treatment, the same time point for sample acquirement of following assays. **d** DNA synthesis assay by BrdU incorporation for cells treated in different conditions, with positive ratios displayed in percentage. Right, representative images (green, BrdU; blue, DAPI). Scale bars, 15 μm. Data analyzed by Student’s *t*-test. **e** Stromal cells were stained in culture for SA-β-Gal, with results presented as positivity ratio. Right, representative images. Scale bars, 20 μm. Data analyzed by Student’s *t*-test. **f** Immunoblot examination of NF-κB pathway in stromal cells treated by bleomycin, with or without RAD001. Cell lysates were fractionated into cytoplasmic and nuclear extracts for comparative analysis. **g** Cells were transfected with a reporter construct that encodes NF-κB binding sites in the promoter region of a luciferase transgene. Signals were normalized as readings of firefly/renilla ratio. Data analyzed by Student’s *t*-test. **h** Co-IP assay to determine the interaction between mTOR and IKK complex. PSC27 cells were treated with bleomycin, and cell lysates were collected 7d later for IP analysis with anti-mTOR or anti-IKKα. IgG, control. Data in all bar plots are shown as mean ± SD and representative of 3 biological replicates. *P < 0.05, **P < 0.01, ***P < 0.001, ^*P* > 0.05

We next asked whether other factors are activated and/or modified after acute cellular responses but contribute to the chronic SASP formation. Contrasting rapid phosphorylation of ATM1 and TAK1 upon genotoxic treatment, the Akt/mTOR pathway was activated at the end of acute response, as evidenced by phosphorylation of both Akt (Ser473) and mTOR (Ser2448), with signaling initiated between 24 h and 3d followed by a platform from 7d post treatment (Fig. 6a). Besides localization of phosphorylated mTOR in the cytoplasm, remarkable alterations of two major mTOR targets including phosphorylation of S6K1/catalytic substrate S6 and 4E-BP1 were observed, substantiating mTOR activation in DNA-damaged cells (Fig. 6b, c). We previously reported that rapamycin reduces phosphorylation of S6K1 and 4E-BP1 in radiation-induced senescent stromal cells, affecting the helicase machinery to suppress translation of mRNAs with stable secondary structure^29^. Herein, we noticed RAD001, a rapamycin analogue, similarly diminished activation of mTOR and its immediate targets, thus confirming the capacity of rapamycin as a SASP inhibitor (Fig. 6c). Although RAD001 prevented mTOR phosphorylation, cell cycle arrest and SA-β-Gal positivity persisted, indicating a maintenance of cellular senescence and metabolic activities (Fig. 6b, d, e).

Although mTOR inhibition diminishes NF-κB transcriptional activity via selective suppression of IL-1α translation^29^, we reasoned whether signaling mediated by such an mTOR-IL-1α axis is the sole mechanism for NF-κB activation. To address this, we first examined the lysates of DNA-damaged stromal cells and observed degradation of IκBα and stabilization of p65 (Rel A), changes that imply NF-κB engagement, and were accompanied by increased NF-κB activity but restrained by RAD001 (Fig. 6f, g). Through mTOR antibody-mediated IP analysis, we found enhanced mTOR association with IKKα and Raptor (Fig. 6h upper). Reciprocal IP with an IKKα antibody showed increased association of IKKα with mTOR but not Raptor (Fig. 6h lower). Thus, the data suggests that IKKα may have a role distinct from other IKK subunits by specifically interacting with mTOR in damaged stromal cells.

To substantiate above findings, we used PP242, a second-generation mTOR inhibitor that selectively targets mTOR kinase activity by competing for ATP binding sites with a mechanism different from that of rapamycin and analogues^37^. Data from in vitro assay indicated that IKKα was specifically phosphorylated as a direct mTOR target after DNA damage (Supplementary Fig. 6a,b). Although mTOR physically interacts with IKKα, the functional relevance of IKKβ, the other catalytic subunit of the IKK complex, remains yet elusive. Interestingly, we found elimination of IKKα substantially decreased the nuclear activity of NF-κB, while depletion of IKKβ affected even more, with minimal NF-κB activity observed upon removal of both subunits (Supplementary Fig. 6c). The data suggested that both α and β subunits transduce signals in DNA-damaged cells.

We recently showed that IL-1α translation promoted by mTOR amplifies the SASP by forming a positive feedback loop between IL-1α and NF-κB^29^. However, how IL-1α controls NF-κB transcriptional activity remains undefined. Here, we observed phosphorylation of IKKβ, degradation of IL-1α receptor-associated kinase 1 (IRAK1) and IκBα, and nuclear translocation of NF-κB subunits after DNA damage (Supplementary Fig. 6d). While RAD001 abolished these alterations, exogenous IL-1α rescued all the responses. To the contrary, elimination of IL-1α reduced IKKβ phosphorylation, whereas IKKα activation remains unaffected (Supplementary Fig. 6e). Further, we observed persistent NF-κB activation even when IL-1α was depleted, as evidenced by decreased IκBα in the cytoplasm and remaining amount of p65 and p50 in the nucleus (Supplementary Fig. 6e).

We next asked whether PI3K/Akt pathway is directly responsible for mTOR activation in genotoxic settings. Subsequent data showed consistently phosphorylated p38 after DNA damage, regardless of PI3K/Akt signaling alteration caused by either treatment of MK-2206, a highly selective allosteric pan-Akt (Akt1/2/3) inhibitor, or elimination of the PI3K catalytic subunit p110 (Supplementary Fig. 6f,g), suggesting p38 functions upstream of PI3K/Akt/mTOR. Further, contrasting either IKKα or IKKβ deficiency, treatment with a p38 inhibitor (SB203580) abolished p38 activity (Supplementary Fig. 6h). DNA damage-induced NF-kB activation and IL-8 expression were significantly reduced upon treatment with several small molecule inhibitors, among which 5Z-7 appeared most effective (Supplementary Fig. 6i,j). As 5Z-7 mainly suppresses TAK1 activity, our data suggested that the SASP may be pharmacologically controlled with a higher efficiency by targeting an upstream kinase of the SASP signaling network in damaged stromal cells.

### Suppression of stromal TAK1 restrains cancer cell malignancy

**Fig. 7:**
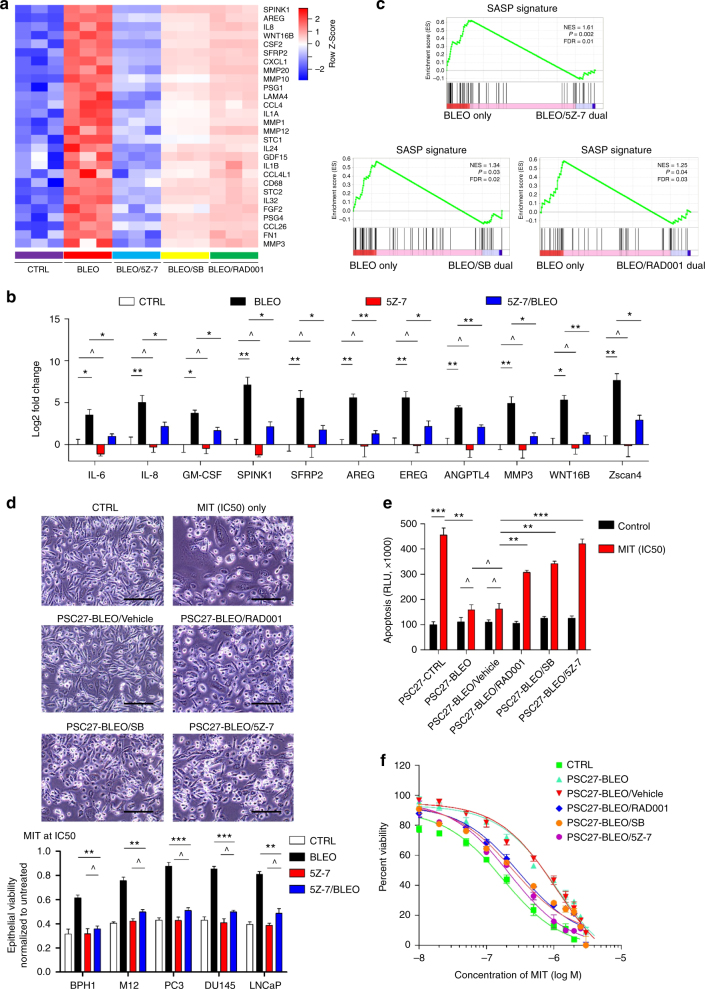
TAK1 inhibition abrogates the SASP development and deprives cancer cells of stroma-conferred malignancy. **a** Heatmap profiling of stromal cell transcriptomics after treatment by bleomycin alone, or together with each of 5Z-7, SB203580 (SB), and RAD001. Cells were collected 7d after drug treatment. The SASP-associated soluble factors are selectively displayed on the top list per expression fold change. **b** Transcript expression assay of several SASP canonical factors. Cells were treated by bleomycin alone or co-treated by bleomycin/SASP inhibitors. Signals were normalized to untreated samples per factor. Data analyzed by Student’s *t*-test. **c** GSEA profiling with significant enrichment scores showing a SASP-specific expression signature composed of canonical soluble factors. Head-to-head comparison of differentially expressed SASP factors was performed between cells treated by bleomycin only and cells co-treated by bleomycin/SASP inhibitors. *P* values determined by GSEA software. **d** The prostate cancer (PCa) cell line PC3 was subject to treatment by mitoxantrone (MIT) while being incubated with several types of conditioned media (CM). Upper, representative images of PC3 cells upon different treatments. Arrows, apoptotic cells; arrowheads, damaged cells. Scale bars, 50 μm. Lower, viability assessment of several cell lines incubated with various types of CM and exposed to MIT at the IC50 concentration pre-determined per line. Data analyzed by Student’s *t*-test. **e** Measurement of apoptosis in culture conditions. Results were depicted in relative luminescence units (RLUs) representing signal readings proportional to caspase-3/7 activity of PC3 cells. Data analyzed by Student’s *t*-test. **f** Dose response curves (non-linear regression fit) of PC3 cells treated by PSC27-derived CM. The concentration of MIT was plotted in an exponential scale, with cell viability assessed in relative to the untreated group and calculated in percentage. Data in all bar plots are shown as mean ± SD and representative of 3 biological replicates.* *P* < 0.05, ** *P* < 0.01, *** *P* < 0.001. BLEO bleomycin, SB SB203580, 5Z-7 5*Z*-7-oxozeaenol

Next, we assessed the biological impact of TAK1 suppression in the TME, specifically cancer cell gain of functions driven by stromal cells developing a SASP. Comparative transcriptomics of stromal cells treated by bleomycin alone and those by bleomycin/5Z-7 dual agents revealed diminished expression of most SASP factors (Fig. 7a). As compared with the consequence generated by SB203580 or RAD001, 5Z-7-mediated TAK1 inhibition appeared to be even more effective in abrogating the expression of most SASP factors and Zscan4 (Fig. 7a, b). Although reduction fold change varied among secreted factors, the overall consistent tendency suggested a substantially weakened SASP phenotype.

Given the prominent efficacy of TAK1suppression in attenuating the SASP, we dissected the SASP phenotype with Gene Set Enrichment Analysis (GSEA). Notably, a pre-defined expression signature comprising the vast majority of the SASP factors was found to be remarkably dampened when TAK1 activity was restrained (*P* < 0.01) (Fig. 7c upper). Although the SASP signature was also weakened when activation of either p38 or mTOR was blocked (*P* < 0.05) (Fig. 7c lower), the output profiles were generally inferior to that of TAK1 suppression. Alternatively, we expanded on data mining with meta-analysis of protein interaction network and found genes downregulated upon TAK1 inhibition were systematically correlated with cytokine/chemokine/ECM-receptor interaction or signaling pathways (Supplementary Fig. 7a–c), further consolidating the efficacy and specificity of TAK1 targeting.

We then evaluated the ability of stromal cell-derived conditioned media (CM) to stimulate cancer epithelial cell proliferation, a basic property of tumour-adjacent stroma. PSC27 cells were treated by bleomycin, with their CM collected 7–10d post treatment and applied to prostate cancer (PCa) cells (Supplementary Fig. 7d), the later chosen due to their same tissue of origin with PSC27 cells. Interestingly, growth potential was widely reduced in all lines tested, which was phenocopied by decreased migration and invasion of PCa cells (Supplementary Fig. 7e–g). More importantly, the ability of a full spectrum of SASP factors in protecting cancer cells against MIT-delivered genotoxicity was markedly compromised, indicating a reduced gain of survival potential upon 5Z-7-mediated stromal TAK1 inhibition (Fig. 7d upper). Cancer cell viability was significantly enhanced by CM from damaged stromal cells, but reduced to almost their basal levels upon TAK1 suppression (Fig. 7d lower). Decreased viability was accompanied by increased apoptotic index of cancer cells upon exposure to MIT-mediated genotoxicity in culture, as evidenced by enhanced caspase 3/7 activities (Fig. 7e). Changes in PCa cell chemoresistance were also reflected by the overall shift of non-linear cell survival curves plotted against cell viability percentage across MIT concentrations from 0.1–1 μM, a range close to serum concentration of this agent delivered to cancer patients^38, 39^ (Fig. 7f). Each case, TAK1 inhibition caused significant changes to the gain of functions of cancer cells conferred by damaged stromal cells, with an efficacy even more prominent than the alterations generated by RAD001 and SB203580 (Fig. 7d–f).

To expand on the generality of the chemoresistance-associated in vitro data, we validated the competence of TAK1 inhibition by applying the same set of CM to PCa cells exposed to docetaxel (DTX), a microtubule toxin widely used in cancer clinics. Again, TAK1 deficiency mediated by 5Z-7 substantially augmented DTX-induced cytotoxic effects (Supplementary Fig. 7h–j). Thus, TAK1 suppression can considerably attenuate the effects of stromal CM on cancer cell malignancy, including acquired resistance to various anticancer agents.

### Targeting TAK1 in vivo sensitizes tumours to chemotherapy

**Fig. 8:**
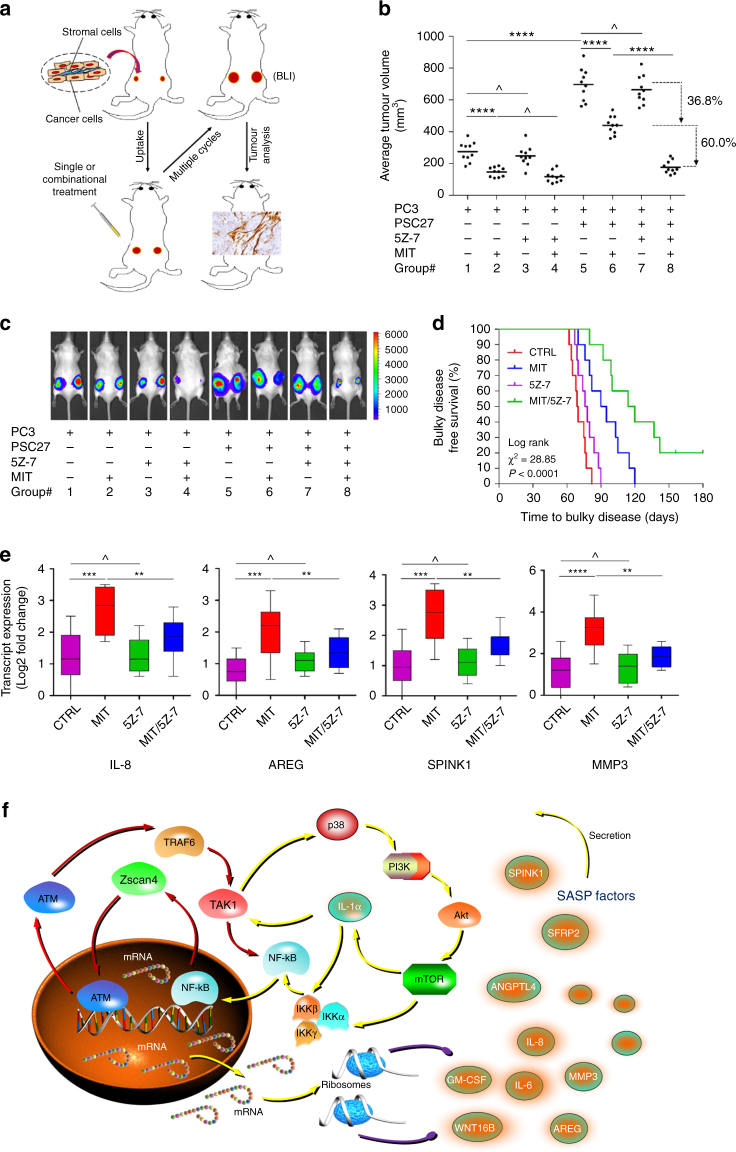
Targeting TAK1 restricts in vivo SASP and minimizes drug resistance acquired from the treatment-damaged TME. **a** Experimental design of severe combined immunodeficient (SCID) mouse-based in vivo studies. Two weeks after tumour implantation and stable uptake, mice received either single or combinational agents administered as metronomic treatments composed of several cycles. **b** Comparative statistics of tumour volumes. PC3 cells were implanted alone or with stromal cells subcutaneously to animals, which were then subject to cyclic treatments. Tumour volumes were measured at the end of an 8-week preclinical regimen. **c** Representative images of tumour-bearing animals in the preclinical trial. Digital signals were proportional to in vivo luciferase activities measured by an IVIS device. **d** Mice were sacrificed upon presence of advanced bulky diseases. Survival duration was calculated from the time of tissue recombinant injection until the day of death. Data compared with log-rank (Mantel–Cox) test. **e** Transcript assessment of several canonical SASP factors expressed in stromal cells isolated from PC3 tumours. Animals that had both stromal and cancer cells in the tumour foci were selected for analysis, with stromal cells acquired by LCM. **f** Graphical summary. DNA damage triggers an acute response of stromal cells, during which Zscan4 is expressed via the ATM-TRAF6-TAK1 axis and translocates to the nucleus. Zscan4 promotes the expression of a subset of ASAP-associated factors through NF-κB signaling, forming the first positive feedback loop. Further, TAK1 collaterally activates p38, a kinase that subsequently engages the PI3K/Akt/mTOR pathway. mTOR subsequently activates the NF-κB machinery both directly via interaction with IKKα and indirectly by promoting the translation of IL-1α, a cytokine that strengthens TAK1 phosphorylation in the chronic SASP. Together, several feedforward mechanisms favor the SASP development until its culmination in stromal cells, which confers substantial resistance on surviving cancer cells in the damaged TME and enables disease recurrence post-therapy. Data in all bar plots are shown as mean ± SD and representative of 3 biological replicates. For animal treatments, *n* = 10/group. Data in **b**, **e** were analyzed by Student’s *t*-test. *P < 0.05, **P < 0.01, ***P < 0.001, ****P < 0.0001, ^*P* > 0.05

Expression of a full SASP in the TME contributes to multiple pathological events including tumorigenesis, systemic inflammation, and therapeutic resistance^11, 12^. However, whether and how the progression toward advanced malignancy can be controlled by specifically controlling the SASP activated by side effects of anticancer treatments, remain as outstanding questions. To mimick the clinical conditions, we generated tissue xenografts by admixing PSC27 and PC3 cells before subcutaneous implantation to SCID mice, which were subject to an 8-week chemotherapeutic regimen designed to cover 3 times of single- or dual-agent administration (Fig. 8a and Supplementary Fig. 8a). In the absence of stromal cells, PC3 xenografts developed tumours even under chemotherapeutic pressure throughout the regimen, but with sizes generally smaller than those comprising both stromal and cancer cells, suggesting considerable pro-tumorigenic effects of the TME (Fig. 8b). Although 5Z-7 alone appeared to have no effect on tumour volume, treatment with the chemotherapeutic agent MIT markedly restrained tumour growth by ~37% (Fig. 8b, lane 5 versus lane 6). Not surprisingly, MIT administration caused prominent in vivo cellular senescence, as evidenced by both IHC and SA-β-gal staining (Supplementary Fig. 8b). Co-administration of MIT and 5Z-7 caused a further shrinkage of tumours and reduced their volumes by 60% (Fig. 8b, lane 6 versus lane 8). Alternatively, to ensure tumours grow subcutaneously at the primary foci without ectopic metastasis, which may interfere with data interpretation, we generated xenografts with PC3 cells stably expressing firefly luciferase to make tumours trackable by bioluminescence imaging (BLI). Signal intensity from these animals appeared largely proportional to virtual tumour sizes and reflective of tumour growth tendency, thus confirming the observed difference between experimental groups (Fig. 8c).

We next evaluated tumour progression consequence by comparing the survival of different animal groups in a time-extended preclinical cohort. All animals were monitored for tumour growth, with bulky disease considered present once the tumour burden was prominent (size ≥2000 mm^3^), an approach described previously^40^. Mice that received MIT/5Z-7 combinational treatment displayed the most prolonged median survival duration, gaining a minimally 50% longer survival when compared with the group treated by MIT alone (Fig. 8d, green versus blue). However, 5Z-7 treatment alone did not provide significant benefit, as it conferred only marginal survival elongation (Fig. 8d, purple versus red). The data suggest that TAK1 inhibition in the TME neither changes tumour growth nor animal survival, while MIT/TAK1 co-treatment can significantly improve both parameters.

Given the striking effects of the TME in promoting tumour progression and chemotherapeutic resistance, we reasoned the efficiency of in vivo targeting of TAK1 by comparing with that of inhibiting mTOR or p38 in chemotherapeutic settings. In similar preclinical trials, we observed that administration of both MIT/RAD001 and MIT/SB203580 caused significant reduction of tumour volumes at the end of regimen. Specifically, MIT/RAD001 co-treatment generated a further shrinkage of tumour size by 44% compared with MIT alone, while MIT/SB203580 co-treatment generated a 46% further reduction (Supplementary Fig. 8c,d). In contrast, the efficacy of TAK1-specific inhibition in the animal studies seemed to be more prominent (Fig. 8b). To validate the effect of in vivo TAK1 targeting on the SASP expression in the TME, we analyzed the tumours particularly those comprising both stromal and cancer cells. Upon LCM-guided isolation of stromal cells from the tumour foci, we found markedly reduced expression of several canonical SASP factors (Fig. 8e and Supplementary Fig. 8e). Notably, treatments performed in these studies appeared to be well tolerated by animals, as no significant perturbations in urea, creatinine, liver enzymes, or body weight were observed (Supplementary Fig. 9a–e). As supporting evidence, data from experimental mice carrying breast tumours composed of MDA-MB-231 cancer cells alone or with HBF1203 stromal cells largely reproduced PCa findings (Supplementary Fig. 10a–i). Specifically, the chemotherapeutic agents and 5Z-7 administered at the doses designed in this study did not significantly interfere with immune system and tissue homeostasis even in immune-competent mice (Supplementary Fig. 11a–d). These results further substantiate that a TAK1 inhibitor combined with conventional chemotherapy has the potential to enhance tumour response without causing severe systemic toxicity.

We finally investigated the correlation of TAK1 with human cancer patient survival. Clinical data suggested that TAK1 activation in the stroma is significantly, albeit negatively correlated with the survival of both NSCLC and BCa patient cohorts (Supplementary Fig. 12a–d). Thus, we concluded that combinational treatment by co-targeting cancer cells and stromal TAK1, a member of the MAPK kinase kinase (MAPKKK) family, may be a promising therapeutic strategy to improve patient outcome in clinical oncology.

## Discussion

The development of resistance to anticancer treatments is a major barrier to cure cancer, and poses a daunting challenge to both clinicians and scientists. Numerous efforts have been made to elucidate resistance mechanisms particularly those involving the tumour-adjacent stroma, but selection of the appropriate target remains an issue of debate. Although various types of cytotoxicity are directly responsible for disease remission, genotoxicity is the most frequently exploited modality. So far, less is understood on how DNA-damaging agents specifically modify the phenotypes of stromal cells in the TME, which frequently causes cancer resistance^1^. Herein, we demonstrate that stromal cells respond to genotoxic, rather than general cytotoxic treatments, in a seemingly static but pathologically dynamic manner via activation of a typical SASP. Specifically, we unraveled intracellular feedforward mechanisms implicated in the SASP development, a paracrine secretory phenotype often utilized by cancer cells to survive frontline pharmaceuticals.

Genotoxicity-induced tissue responses in the TME can arise rapidly following drug administration, as exemplified by the release of IL-6 from both human and mouse endothelial cells in the thymus within 24 h of chemotherapy during the ASAP^19, 20^. Distinct from the ASAP, however, the SASP typically develops over a course of 6 to 8d and occurs only after establishment of senescence-associated markers, such as p16 and SA-β-Gal^8^. Although the ASAP is an acute response with relatively limited influence to tissue homeostasis, the SASP develops as a chronic phenotype that holds the potential to exert long term effects in vivo and favors multiple aging-related pathologies including cancer^21, 41^. A recent study further proved that the ASAP and the SASP are not only temporally, but also mechanistically distinct, as the expression pattern of secretory factors in endothelial cells at 24 h clearly differs from that of 5d after chemotherapy^42^. Although genotoxic agents can trigger senescence, the ASAP is limited in both duration and composition when compared with the SASP^42^. Nevertheless, how the ASAP converts into the SASP after the onset of treatment remains elusive and represents an intriguing topic for the scientific community.

Here, we scrutinized the genome-wide expression microarray data of human stromal cells treated by two classical groups of chemotherapeutic agents. Upon comparison of stromal expression profiles generated by these drugs, we noticed Zscan4, which stands on the top list of upregulated genes including multiple SASP factors. Of note, Zscan4 is also one of the ASAP factors expressed rapidly after DNA damage. Depletion of Zscan4 not only affected the efficiency of DNA damage repair, but also markedly diminished the SASP intensity, suggesting a pivotal role of Zscan4 in regulating DDR and sustaining the SASP. We further discovered Zscan4 involvement in a positive feedback loop formed within the first 2d after DNA damage, a period when the DDR-induced ASAP culminates. As genotoxic treatment causes comprehensive DNA damage across whole chromosomes, generation of DSB lesions on telomeres should be inevitable. Given that telomeric DNA damage is essentially irreparable and triggers persistent DDR signaling, the latter responsible for the long term SASP maintenance^14, 43^, we speculate that Zscan4 is specifically implicated in telomeric DNA damage repair, and transmit signals from the ASAP-associated DDR machinery. Despite our evidence of substantial Zcan4 translocation to the nucleus after synthesized in the cytoplasm, whether and how Zscan4 associates with central DDR components, such as the MRN complex, ATM, γ-H2AX, and p53-BP1, or more specifically, telomere binding factors that compose the shelterin complex^44^, remains an open question and deserves continued investigation.

As part of the genotoxic stress response, ATM is phosphorylated and translocates to the cytoplasm of stromal cells, where it induces TRAF6 auto-ubiquitination and TAK1 activation, forming an ATM-TRAF6-TAK1 axis. Among these molecules, TAK1 seems to be a special kinase in DNA damage events, as we found it is indeed critical for multiple events even after the ASAP surge, including p38 activation. In contrast to the ATM-TRAF6-TAK1 axis, which is formed rapidly after genotoxic treatment, p38 phosphorylation is subject to TAK1 catalytic activity and occurs at a time between the ASAP and the SASP. Indeed, p38 activation is an event upstream of PI3K/Akt/mTOR pathway, but after TAK1 implication in the treatment-induced acute response. Complementing the data of former studies reporting activation of p38 as a major SASP regulator via control of RNA stability and PI3K/AKT/mTOR pathway as a molecular rheostat that determines SASP factor production^27, 42^, our work clarifies the sequential molecular events including p38 engagement and PI3K/AKT/mTOR activation in genotoxic settings. Thus, DNA damage triggers the ASAP by generating the ATM-TRAF6-TAK1 axis, wherein the kinase TAK1 subsequently initiates lateral signaling events including activation of p38 and PI3K/AKT/mTOR, together potentiating the chronicity of the SASP via several feedforward mechanisms (Fig. 8f). Our work makes an essential contribution to the SASP biology by unmasking the molecular events starting from the acute response to Zscan4 expression, TAK1 activation, p38/PI3K/mTOR implication, and IL-1α/NF-kB engagement, a cellular signaling network that underpins the SASP development in a well-ordered and precisely-tuned manner.

The dark side of the SASP-linked events in clinical oncology has raised the possibility that therapeutic targeting senescent cells including those generated by clinical drugs is a promising option to overcome treatment-induced side effects. Senotherapies represent a new wave of efforts that mainly exploit various chemical and/or biological approaches to remove senescent cells by apoptotic (senoptosis) or non-apoptotic (senolysis) means^21^. However, given the beneficial functions of cellular senescence including its contribution to wound healing and tissue repair in aged individuals^45^, particularly during recovery after anticancer treatment, selectively inhibiting deleterious SASP factor expression rather than eradicating the senescent cells per se, appears to be more sagacious by circumventing the potential pitfalls, particularly in the current era of precision medicine.

## Methods

### Cell lines and cell culture

Human primary prostate stromal cell line PSC27 and primary breast stromal cell line HBF1203 were gifts from Dr. Peter Nelson, and were maintained in PSC complete medium as reported^11^. Prostate epithelial cell lines PC3, DU145, and LNCaP (ATCC) were routinely cultured in RPMI 1640 (5% FBS) as recommended. BPH1 was from prostatic tissue with benign hyperplasia, immortalized by SV40-LT antigen and cultured as documented^46^, while M12 was derived from BPH1, but essentially neoplastic and metastatic^47^. Both BPH1 and M12 were generously provided by Dr. Stephen Plymate. All cell lines were routinely tested for microbial contamination (including mycoplasma) and authenticated with STR assays.

### Human lung cancer and breast cancer patient samples

Administration of chemotherapeutic agents was performed for relapsed and refractory NSCLC patients (Clinical trial no. NCT02889666), and infiltrating ductal BCa patients (NCT02897700) by following the CONSORT Statement to report randomized trials. Patients with a clinical stage ≥I subtype A (IA) (T1a, N0, M0) of primary cancer but without manifest distant metastasis were enrolled into the randomized, controlled and multicentered pilot studies. Age ≤75 years with histologically proven NSCLC, or age ≥18 years with histologically proven infiltrating ductal BCa was required for recruitment into the individual clinical cohorts. Written informed consent was provided by all patients. Data regarding tumour size, histological type, tumour penetration, lymph node metastasis, and pathologic TNM disease stage were obtained from the pathologic records. Tumours were processed as FFPE biospecimens and sectioned for histological assessment. OCT-frozen chunks were processed via LCM for gene expression analysis. Specifically, stromal compartments associated with glands and cancer epithelium were separately isolated from tumour biopsies before and after chemotherapy using an Arcturus (Veritas Microdissection) laser capture microscope following the criteria defined formerly^11^. The immunoreactive scoring (IRS) gives a range of 1–4 qualitative scores according to staining intensity per tissue sample. Categories for the IRS include 0–1 (negative), 1–2 (weak), 2–3 (moderate), and 3–4 (strong)^48^. The diagnosis of NSCLC and BCa tissues was confirmed based on histological evaluation by independent pathologists. Randomized control trial (RCT) protocols and all experimental procedures were approved by the Institutional Review Board of Shanghai Jiao Tong University School of Medicine, with methods carried out in accordance with the official guidelines and relevant ethical regulations strictly followed.

### Cell treatments and senescence appraisal

PSC27 cells were grown until 80% confluent (CTRL) and treated with docetaxel (10 nM, DTX), paclitaxel (10 nM, PTX), vincristine (20 nM, VCR), bleomycin (50 μg/ml, BLEO), and mitoxantrone (500 nM, MIT) for 6 h or γ-radiation by a ^137^Cs source at 743 rad/min for 10 Gy (RAD). Alternatively, HBF1203 cells were treated with docetaxel (10 nM, DTX), paclitaxel (10 nM, PTX), vincristine (20 nM, VCR), doxorubicin (10 μM, DOX), carboplatin (200 μM, CARB), or cisplatin (100 μM, CIS) for 6 h. After treatment, the cells were rinsed thrice with PBS and allowed to stay for 7–10 days in media. Alternatively, the cells were allowed to passage consecutively for replicative exhaustion. To examine cellular senescence, SA-β-Gal staining and BrdU incorporation were performed with the commercial kits (BioVision and BD Biosciences, respectively) as previously reported^49^. Specifically, a single pulse of BrdU (10 μM) was performed for 12 h and cells were subject to immunofluorescence staining with an anti-BrdU antibody (1:1000 dilution, Cell Signaling, 5292) before counterstained with DAPI and assessed by confocal microscopy. DNA-damage extent was evaluated by immunostaining for γ-H2AX or 53BP1 foci by following a 4-category counting strategy as formerly reported^11^. Random fields were chosen to show DDR foci and SA-β-gal positivity, while BrdU incorporation and DDR foci were quantified using CellProfiler (http://www.cellprofiler.org) with a minimum of 200 cells counted per sample for above cell-based assays. For clonogenic assays, cells were seeded at 1 × 10^3^ cells/dish in 10 mm dishes for 24 h before treated with bleomycin. The cells were fixed with 2% paraformaldehyde 7–10 days post treatment, gently washed with PBS, and stained instantly with 10% crystal violet prepared in 50% methanol. Excess dye was removed with PBS, with plates photographed. Colony formation were evaluated by quantifying the number of single colonies per dish.

### Quantitative PCR, immunoblotting, and immunofluorescence

Quantitative real-time PCR was performed using the reagents of Applied Biosystems. Sequences of gene-specific primers used for this study are listed in Supplementary Table 1. For immunoblotting, cells were lysed in 0.15 M NaCl, 20 mM Tris-HCl (pH7.9), 10% glycerol, 0.1% NP40, with complete protease/phosphatase cocktail inhibitors (Bimake). Lysates were centrifuged at 10,000× *g* for 10 min, with supernatants applied to standard procedures of SDS-PAGE, transferred to nitrocellulose membrane. Proteins were identified by incubating with appropriate primary antibodies at dilutions as indicated in Supplementary Table 2 (antibodies section). The scans of the most important blots are shown at Supplementary Fig. 13. For immunofluorescence staining, cells were cultured in dishes and pre-seeded for at least 24 h on coverslips. After brief washing, the cells were fixed with 4% paraformaldehyde in PBS for 8 min, blocked with 5% normal goat serum (NGS, Thermo Fisher) for 30 min, and incubated with primary antibodies diluted in PBS containing 5% NGS for 2 h at room temperature. Alexa Fluor 488 or 595-conjugated secondary antibodies (Invitrogen, 1:400) were used. Nuclei were stained with Hoechst 33342, with slides mounted with Vectashield medium. Fluorescence imaging was performed on a fluorescence microscope (Nikon Eclipse Ti S). Captured images were analyzed and processed with the Nikon DS-Ri2 fluorescence workstation and processed with NIS-Elements F4.30.01. Alternatively, a confocal microscope (Zeiss LSM 780) was applied to acquire confocal images.

### Immunohistochemistry

The tissue specimens were fixed overnight in 10% neutral-buffered formalin before dehydrated in increasing concentrations of isopropyl alcohol and cleared of alcohol by xylene. The specimens were then embedded in paraffin wax in cassettes for facilitation of tissue sectioning. Standard staining with hematoxylin and eosin was performed on sections of 5 mm thickness processed from each specimen block. For immunohistochemistry, liver sections were deparaffinized and incubated in citrate buffer at 95 °C for 40 min for antigen retrieval before incubated overnight at 4 °C with the primary antibodies including anti-Zscan4 (1:200 dilution, Abclonal, A12015), anti-p-p38 (1:200 dilution, R&D, AF869), anti-p-mTOR (1:500 dilution, Cell Signaling, 2971). After 3 washes, tissue sections were incubated with biotinylated anti-mouse IgG (1:200 dilution, Vector Laboratories) for 1 h at RT then washed 3 times, after which streptavidin–horseradish peroxidase conjugates (Vector Laboratories, CA, USA) were added and the slides incubated for 45 min. After 3 washes with PBS, DAB solution (Vector Laboratories) was added and the slides were counterstained with haematoxylin.

### NF-κB reporter assays

NF-κB transcription activity was determined by luciferase signals derived from a NAT11-Luc2CP-IRES-nEGFP construct^11^ that encodes multiple copies of NF-κB binding sequences and an optimized IL-2 minimal promoter as part of NF-κB-activated transgene (NAT) system. The reporter vector (firefly) was co-transfected with pRL-TK plasmid (renilla) for transfection efficiency controls and internal normalization for gene expression. Cell lysates were analyzed for luciferase activities using a luminometer and a commercial kit (Promega).

### Human genome-wide microarray

Total RNAs from experimental samples were isolated using the RNeasy plus mini kit (Qiagen), incorporating on-column DNase treatment using the RNase-Free DNase Set. RNA quantity and quality were assessed by a NanoDrop ND-1000, with RNA integrity determined by standard denaturing agarose gel electrophoresis. Arraystar Human LncRNA microarray v3.0 designed for global profiling of human LncRNAs (32,586) and protein-coding transcripts (26,109) covering all entries of Refseq, UCSC known genes and Gencode was employed. RNA labeling and array hybridization were performed according to a one-color microarray-based gene expression analysis protocol (Agilent) with minor modifications. Briefly, mRNA was purified from total RNA after removal of rRNA. Each sample was amplified and transcribed into fluorescent cRNA along the entire length of the transcripts without 3′ bias utilizing a mixture of oligo(dT) and random primers (Arraystar Flash RNA labeling kit, Arraystar). The labeled cRNAs were purified by RNeasy Mini kit (Qiagen). The concentration and specific activity of the labeled cRNAs (pmol Cy3/μg cRNA) were measured by NanoDrop ND-1000. One microgram of each labeled cRNA was fragmented by adding 5 μl 10 × blocking agent and 1 μl of 25 × fragmentation buffer, then heated the mixture at 60 °C for 30 min, finally 25 μl 2 × GE hybridization buffer was added to dilute the labeled cRNA. A volume of 50 μl of hybridization solution was dispensed into the gasket slide and assembled to the LncRNA expression microarray slide (Arraystar). The slides were incubated for 17 h at 65 °C in an Agilent hybridization oven. The hybridized arrays were washed, fixed and scanned using the Agilent DNA microarray scanner (G2565BA), with data extracted with the Agilent Feature Extraction software (v11.0.1.1). Resulting single channel raw data were analyzed with Agilent GeneSpring GX software (v12.1). Raw datasets were imported and log2-transformed according to the recommended default procedure and were subsequently quantile normalized. Entities showing technical impairments were excluded from final analysis.

### GO and network analyses

Gene transcripts expressed in PSC27 stroma cells were analyzed with PANTHER (http://www.pantherdb.org) and Ingenuity Pathway Analysis (IPA) (http://www.ingenuity.com/products/ipa). Using PANTHER, protein classification was performed according to three ontological terms: biological process (BP), CC, and molecular function (MF). For PANTHER analysis, we used statistics overrepresentation (i.e., the default setting) to compare classifications of multiple clusters of lists with a reference list to statistically identify the over- or underrepresentation of PANTHER ontologies. Venn diagram was prepared with Bioinformatics and Evolutionary Genomics (http://bioinformatics.psb.ugent.be). Significance was set to a *p*-value of 0.05. NetworkAnalyst^50^ was used to construct and visualize molecular nodal networks, based on the differentially expressed genes (DEG) generated from the transcriptomics database of stromal cells subject to DNA damage and/or SASP inhibition. The IMEx Interactome database was employed for protein-protein interaction (PPI) analysis, with literature-curated comprehensive data from InnateDB^51^.

### Promoter analysis and ChIP assays

A 4000 bp region immediately upstream of the human Zscan4 gene (Gene ID 201516, Genbank accession NM_152677.2) transcription start site was analyzed for core NF-κB binding sites using the CONSITE computerized DNA-binding motif search program. Three PCR primer sets were designed to amplify representative regions within the proximal promoter sequence (primer sequences available in Supplementary Table 3). As positive controls, four additional primer sets were used to amplify individual regions that encompass known NF-κB binding sites within the promoters of the human WNT16B, SFRP2, IL-6, and IL-8 genes, respectively. ChIP assays were performed on PSC27 cells of early passage (p10, as untreated) and those damaged by bleomycin at 50 μg/ml in culture (treated). Chromatin fixed by 2% formaldehyde was immunoprecipitated using monoclonal mouse anti-p65 antibody (F-6, Santa Cruz). DNA was extracted from the precipitates upon reverse crosslinking, and amplified using the primer sets mentioned above. The immediate 5′ upstream sequences containing putative NF-κB binding motifs of Zscan4 were amplified from human genomic DNA, with PCR products sequentially cloned into a pCR2.1-TOPO vector (Invitrogen) and a reporter luciferase vector pGL4.22 vector (Promega). The NAT11-Luc2CP-IRES-nEGFP construct that contains multiple copies of typical NF-κB binding sequences and an optimized IL-2 minimal promoter as part of NAT system was used as positive control, and a pRL-TK vector (Addgene) was co-transfected for signal normalization.

### ELISA assay of canonical SASP factors

CM were collected in serum free media after a 72 h culture of cells, after which cells were immediately counted for normalization. CM were adjusted to match the equivalent numbers of cells per sample. Human IL-8 immunoassay was performed using kits as recommended by the manufacturers (Perkin Elmer and Enzo Life Sciences, respectively).

### Co-immunoprecipitation

Cells were rinsed twice with cold PBS then lysed on ice for 20 min in 1 ml of lysis buffer (40 mM Hepes at pH 7.5, 120 mM NaCl, 1 mM EDTA, 10 mM pyrophosphate, 10 mM glycerophosphate, 50 mM NaF, 0.5 mM orthovanadate, EDTA-free protease inhibitors) containing 0.3% CHAPS. Four micrograms of antibodies specific to target proteins were added to the cleared cellular lysates and incubated with rotation for overnight. Then, 50 μl of protein A/G-agarose beads (Pierce) were added and the incubation continued for 12 h at 4 °C. Immunoprecipitates captured with the beads were washed thrice with the CHAPS lysis buffer and two times by wash buffer A (50 mM Hepes at pH 7.5, 150 mM NaCl, protease phosphatase inhibitors included), and boiled in 4 × SDS sample buffer prior to electrophoresis and immunoblotting.

### In vitro kinase assays

PSC27 cells either untreated or treated in various conditions were lysed in 1 ml of lysis buffer with 0.3% CHAPS. Constructs encoding Flag-IKKα and GST-mTOR were co-transfected into cells 48 h prior to bleomycin treatment. Total cell lysates were collected 72 h after treatment (a time point when mTOR activity was prominent), while PP242 (Selleckchem) and vehicle control were applied 3 h prior to bleomycin addition to restrain mTOR kinase activity and prevent phosphorylation of IKKα before cells were damaged by genotoxicity. Lysis buffer contains protease/phosphatase inhibitors and 20 nM PP242, with lysates incubated with anti-Flag antibody for 12 h, followed by another 1 h of incubation with 25 μl of protein G-agarose beads. Anti-Flag immunoprecipitates were washed 4 times with lysis buffer to remove PP242 and 3 times with kinase buffer without ATP (20 mM Hepes at pH 7.7, 2 mM MgCl_2_, 2 mM MnCl_2_, 10 mM β-glycerophosphate, 10 mM NaF, 10 mM p-Nitrophenyl Phosphate [PNPP], 300 μM orthovanadate, 1 mM Benzamidine, 2 mM PMSF, 1 mM DTT, 10 μg/ml aprotinin, 1 μg/ml Leupeptin, 1 μg/ml pepstatin, 1 mM DTT). Immunoprecipitates were also incubated with calf intestinal phosphatase or vehicle control for 45 min at 37 °C at room temperature to inhibit the co-precipitating mTOR activity, thus serving as a specific control. Kinase assay toward recombinant GST-mTOR using washed immunoprecipitates was performed for 60 min at 30 °C in 30 μl of kinase buffer with 10 μM ATP and [γ-^32^P]ATP (0.5 μCi for per kinase reaction). Since no exogenous mTOR was available following immunoprecipation, all IKKα phosphorylation observed during this assay must be due to co-precipitating GST-mTOR. To stop the reaction, 8 μl of 4 × SDS sample buffer was added to each reaction, which was boiled for 10 min. The reaction was then separated by 4%–12% SDS-PAGE and transferred to PVDF membrane. Immunoblot analysis using the p-IKKα antibody was then carried out to examine IKKα phosphorylation status in the immunoprecipitates. Alternatively, ^32^P incorporated into Flag-IKKα was assessed by autoradiography to confirm the results. An antibody against the GST tag was used in parallel immunoblots to examine the expression of mTOR in the cell lysates to confirm its presence after transfection. For TAK1 kinase assay, immunoprecipitates were incubated with 5 μCi of [γ-^32^P]ATP (3000 Ci/mM, Perkin Elmer) and 1 μg of commercial MKK6 (Origene) in the 10 μl kinase buffer (10 mm HEPES, pH 7.4, 1 mm dithiothreitol, 5 mm MgCl_2_) at 25 for 10 min. Resulting samples were subject to 12% SDS-PAGE separation and visualized with autoradiography.

### Cancer cell phenotype assays

Epithelial cell proliferation was determined following the MTT procedure (Promega). Migration and invasion were assessed in culture using Transwells (Cultrex 24-well Cell Migration Assay plates) containing a porous (8 μm pore size) membrane uncoated (for the migration assay) or coated (for the invasion assay) with a 0.5 × solution of basement membrane extract and the indicated CM in the bottom portion of the well. After 24 h, migrating or invading cells on the bottom side of the porous membranes were stained and quantified by absorbance as recommended by the supplier.

### Clonogenic assay for stromal cells

PSC27 cells were trypsinized and counted with a hemocytometer. Two milliliters of DMEM full medium containing 500 cells were plated in each well of the six-well plates. The cells were maintained at 37 °C for 7 days to allow colony formation before stained with 0.5% crystal violet (Sigma-Aldrich) in absolute methanol. Colonies per well were counted and numbers were recorded from three independent experiments.

### Mouse experiments

ISR SCID male mice (Taconic) of 6–8 weeks old were housed and maintained in accordance with the guidelines of Institutional Animal Care and Use Committees (IACUC) at University of Washington and Shanghai Institutes for Biological Sciences, Chinese Academy of Sciences. ARRIVE (Animals in Research: Reporting In Vivo Experiments) guidelines were followed to promote high-quality, comprehensive reporting to allow an accurate critical review of laboratory animal procedures. Briefly, human prostate stromal cells (PSC27) and cancer epithelial cells (PC3) were mixed at a ratio of 1:4, with each ex vivo recombinant comprised of 1.25 × 10^6^ cells prior to subcutaneous implantation. Two weeks later, mice were randomized into groups and subject to preclinical treatments. RAD001 (Selleckchem), SB203580 (MedChem Express), or 5Z-7-Oxozeaenol (TOCRIS) solution was freshly prepared by diluting a stock into 30% propylene glycol/1% Tween 80/H_2_O to make an injection agent. Animals were treated with mitoxantrone (0.2 mg/kg) alone or mitoxantrone plus SASP inhibitors (RAD001 at 2.5 mg/kg, SB203580 at 4 mg/kg or 5Z-7-Oxozeaenol at 5 mg/kg). Agents were delivered intraperitoneally (i.p.) once per biweekly starting from the beginning of the 3rd week, with totally three 2-week cycles throughout the therapeutic regimen. Mice were sacrificed at end of the 8^th^ week post tumour xenograft. Primary tumour size was measured upon animal dissection, with approximate ellipsoid tumour volume (*V*) measured and calculated according to the tumour length (*l*) and width (*w*) by the formula: *V* = (π/6) × ((*l* + *w*)/2)^3^ used previously. Excised tumours were either freshly snap-frozen, or fixed in 10% buffered formalin and subsequently processed as formalin-fixed and paraffin-embedded sections (FFPE) for IHC staining. Alternatively, to measure overall survival, animals were allowed to live until they have developed bulky disease, which was considered present once the tumour burden became prominent (volume ≥1000 mm^3^) in the mouse abdomen. When at least five of the 10 mice in a treatment group had bulky disease, the median survival duration for that group was considered reached. Tumour growth in mice was evaluated using the bioluminescence emitted by PC3-luc cells which stably express the firefly luciferase. The Xenogen IVIS Imager (Caliper Lifesciences) was applied to document BLI across the visible spectrum, with the substrate D-Luciferin (150 mg/kg, BioVision) injected subcutaneously each time freshly for tumour surveillance.

Alternatively, breast stromal cells (HBF1203) and cancer epithelial cells (MDA-MB-231) were mixed in a way that resembles the one applied to prostate stromal cells and epithelial cells, and implanted to subcutaneous sites. Two weeks later, mice were randomized into groups and subject to preclinical treatments. Animals were treated with doxorubicin (0.5 mg/kg doses), alone or combined with SASP inhibitors (RAD001 at 2.5 mg/kg, SB203580 at 4 mg/kg or 5Z-7-Oxozeaenol at 5 mg/kg). Agent delivery, tumour surveillance and volume measurement were performed by following the procedure established for prostate tumour assays. Tumour-bearing animals were randomized for treatment with chemotherapeutic agents and/or SASP inhibitors. Investigators were blinded to the group allocation while assessing experimental outcomes, with the group identities appropriately masked. All experiments were done with approval from the Institutional Animal Care and Use Committee of University of Washington and Shanghai Institutes for Biological Sciences, Chinese Academy of Sciences.

### Toxicity appraisal

Mouse blood was collected by cardiac puncture at the completion of therapeutic regimen, and placed in microcuvette (Sarstedt) and microtainer tubes (BD Pharmingen). Full blood examination was subsequently carried out on an Advia 2120 blood analysis machine (Siemens), with serum analysis conducted on an Architect auto-analyzer (Abbott) after serum separation according to the manufacturer’s instructions.

To evaluate the potential cytotoxic influence of therapeutic agents on immune-competent animals, we followed above preclinical treatment regimen with C57BL/6 mice of 6–8 weeks old (Taconic), but waived tumour implantation performance. Blood examination was performed as described above, with the number of whole blood cells, lymphocytes and neutrophils counted for overall pathological appraisal.

### Quantification and statistical analyses

All in vitro data were representative of at least three independent experiments, and all animal work included at least 10 mice per group except conditions stated specifically. Each statistical analysis was performed with GraphPad Prism 6, data presented as the mean ± SD, and the significance calculated with Student’s unpaired two-tailed *t*-test. Survival data were processed with Kaplan–Meier method (clinical and histopathological data provided in Supplementary Tables 4 and 5). The ratio of the relative expression of the indicated factors from the tumour patients was plotted and applied with the linear regression *t*-test. Comparisons between the means of more than two groups were assessed using one-way ANOVA analysis followed by a Tukey’s post hoc test (95% confidence), while comparisons made between groups with more than two variables were conducted by two-way ANOVA followed by Bonferroni’s post hoc test (95% confidence). In each case, a *P*-value of <0.05 is considered statistically significant per analysis, and variance was similar between statistically compared groups.

To determine sample size, we began by setting the values of type I error (*α*) and power (1-*β*) to be statistically adequate: 0.05 and 0.80, respectively^52^. We then determine *n* on the basis of the smallest effect we wish to measure. If the required sample size is too large, we chose to reassess the objectives or to more tightly control the experimental conditions to reduce the variance.

### Data availability

Microarray data have been deposited in the NCBI GEO (http://www.ncbi.nlm.nih.gov/geo) under accession numbers GSE82033 and GSE90866. All microarray experiments were performed as independent triplicates, with samples collected from human PSC27 stromal cells treated as indicated for specific assays. All relevant data supporting the key findings are available from the corresponding author upon reasonable request.

## Electronic supplementary material

Supplementary Information
