# Supplementary material for: The senescence-associated secretory phenotype is potentiated by feedforward regulatory mechanisms involving Zscan4 and TAK1
Source: Nat Commun. 2018 Apr 30;9:1723. doi: 10.1038/s41467-018-04010-4 (PMC5928226; doi:10.1038/s41467-018-04010-4)
Supplement: Supplementary file 1 — Supplementary Information [file 41467_2018_4010_MOESM1_ESM.pdf]

The senescence-associated secretory phenotype is potentiated by feedforward regulatory mechanisms involving Zscan4 and TAK1

Sun, Y. et al

File Name: Supplementary Information

Description: Supplementary Figures and Supplementary Tables

****

Supplementary Figures: Supplementary Figure 1-13

Supplementary Tables: Supplementary Table 1-5

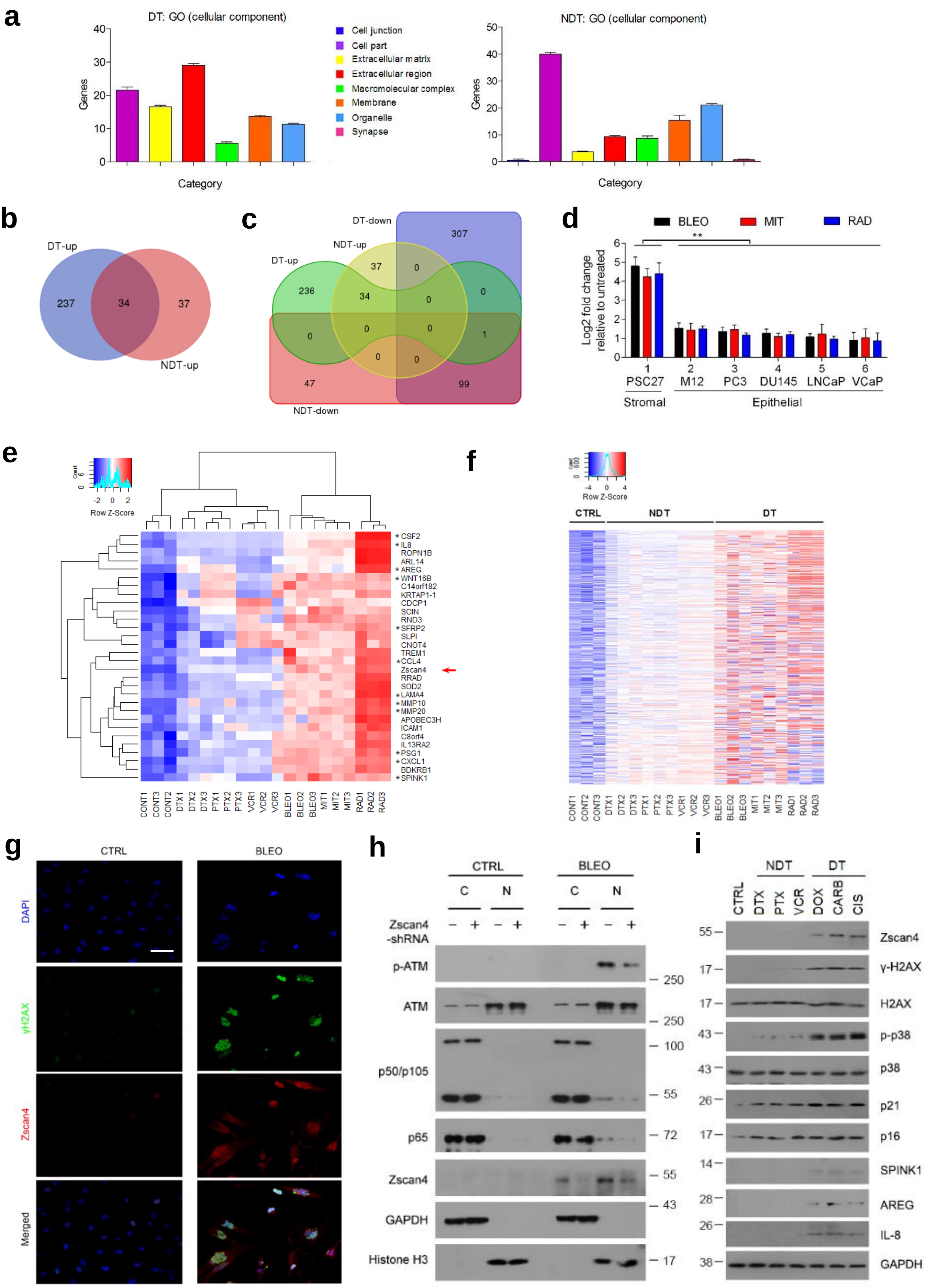

a
DT: GO (cellular component)
Genes
Category
Cell junction
Cell part
Extracellular matrix
Extracellular region
Macromolecular complex
Membrane
Organelle
Synapse
NDT: GO (cellular component)
Genes
Category
b
DT-up
237
34
37
NDT-up
c
DT-down
307
NDT-up
DT-up
37
0
236
34
0
0
0
0
0
1
0
0
47
99
NDT-down
d
BLEO
MIT
RAD
**
Log2 fold change relative to untreated
1 PSC27
2 M12
3 PC3
4 DU145
5 LNCaP
6 VCaP
Stromal
Epithelial
e
Row Z-Score
*CSF2
*IL8
ROPN1B
ARL14
*AREG
*WNT16B
C14orf182
KRTAP1-1
CDCP1
SCIN
RND3
*SFRP2
SLPI
CNOT4
TREM1
*CCL4
Zscan4
RRAD
SOD2
*LAMA4
*MMP10
*MMP20
APOBEC3H
ICAM1
C8orf4
IL13RA2
*PSG1
*CXCL1
BDKRB1
*SPINK1
CONT1 CONT3 CONT2 DTX1 DTX2 DTX3 PTX1 PTX2 PTX3 VCR1 VCR2 VCR3 BLEO1 BLEO2 BLEO3 MIT1 MIT2 MIT3 RAD1 RAD2 RAD3
f
Row Z-Score
CTRL
NDT
DT
CONT1 CONT2 CONT3 DTX1 DTX2 DTX3 PTX1 PTX2 PTX3 VCR1 VCR2 VCR3 BLEO1 BLEO2 BLEO3 MIT1 MIT2 MIT3 RAD1 RAD2 RAD3
g
CTRL
BLEO
DAPI
γH2AX
Zscan4
Merged
h
CTRL
BLEO
C
N
C
N
Zscan4 -shRNA
p-ATM
ATM
p50/p105
p65
Zscan4
GAPDH
Histone H3
250
250
100
55
72
55
43
17
i
NDT
DT
CTRL
DTX
PTX
VCR
DOX
CARB
CIS
Zscan4
γ-H2AX
H2AX
p-p38
p38
p21
p16
SPINK1
AREG
IL-8
GAPDH
55
17
17
43
43
26
17
14
28
26
38

Supplementary Figure 1. Gene expression profiling of human stromal cells treated by chemotherapeutic agents and subcellular localization of Zscan4. (**a**) Cellular component depiction for upregulated genes (top 100 per agent group) of PSC27 cells treated by the DT and NDT agents, respectively. Color annotations are shown aside the bar plots. (**b**) Venn diagram displaying genes upregulated by the DT and NDT agents, respectively. There were 271 unique genes with an upregulation fold change > 3 for the DT group, whereas 71 for the NDT group. (**c**) Venn diagram presenting both upregulated and downregulated genes by these two agent groups. In addition to the upregulated genes selected above, there were 407 unique genes with a downregulation fold change > 3 for the DT group, whereas 147 genes for the NDT group. (**d**) Quantitative RT-PCR measurement of Zscan4 induction in prostate stromal cells and cancer epithelial cells, respectively, in response to treatment by bleomycin (50 µg/ml), mitoxantrone (500 nM) or radiation (10 Gy). Data normalized to the untreated sample per line, and analyzed using two-way ANOVA. (**e**) Heatmap depiction of top 30 human genes that are significantly upregulated in stromal cells after treatment by DT agents, with expression values in stromal cells treated by NDT agents shown side-by-side for parallel comparison. Hierarchical clustering was performed for both treatments and genes. Red arrow, Zscan4. Asterisks, genes encoding soluble factors. (**f**) Genome-wide profiling of human gene expression, by comparing the first 3107 genes upregulated by DT agents (fold change > 1) versus NDT agents. Hierarchical clustering was waived to allow for condensed heatmap presentation. (**g**) PSC27 cells were subject to bleomycin treatment (50 µg/ml). Cells were fixed 7 days post-treatment and analyzed with immunofluorescence microscopy (γ-H2AX and Zscan4 co-stained). Images are representative of 3 independent experiments, scale bar, 20

μm. (**h**) Immunoblot analysis of Zscan4 expression and localization in PSC27 cells 7 days after bleomycin treatment. Upon separate preparation of cytoplasmic and nuclear proteins, ATM activation, NF-κB complex subunits (p50, p65) nuclear translocation and Zscan4 expression were assessed simultaneously. GAPDH and Histone H3, loading controls for cytoplasmic and nuclear samples, respectively. (**i**) Expression profiling of proteins including Zscan4, H2AX, p38, p16, p21 and a subset of hallmark SASP factors in human breast stromal cell line HBF1203 by immunoblots. Cells were treated by a group of NDT and DT agents (DTX, docetaxel; PTX, paclitaxel; VCR, vincristine; DOX, doxorubicin; CARB, carboplatin; CIS, cisplatin), with lysates collected 7 d after drug treatment. Unless noted, data in bar plots are shown as mean ± SD and representative of 3 biological replicates. * $P < 0.05$, ** $P < 0.01$, ^ $P > 0.05$.

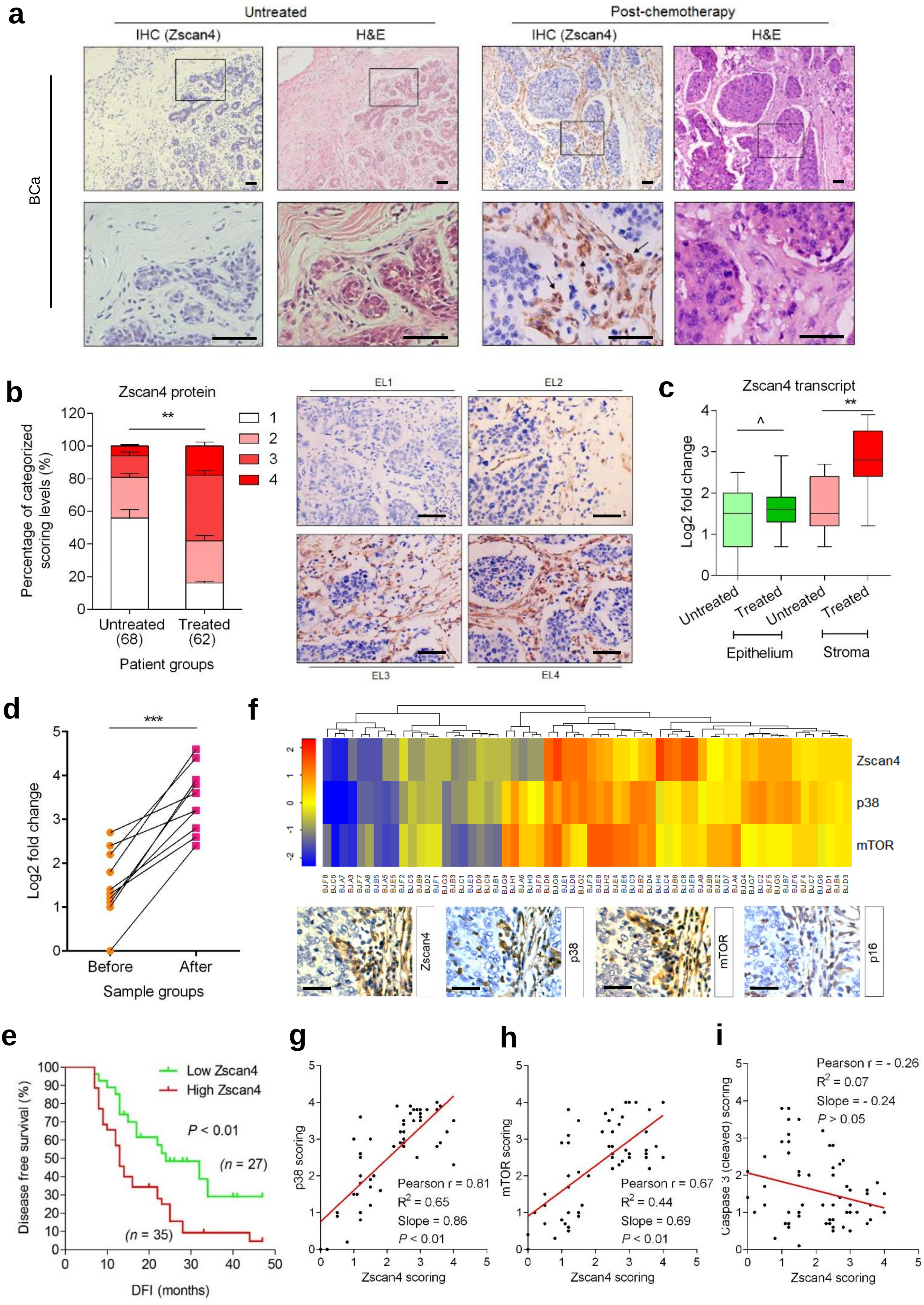

a
Untreated
Post-chemotherapy
IHC (Zscan4)
H&E
IHC (Zscan4)
H&E
BCa
b
Zscan4 protein
**
Percentage of categorized scoring levels (%)
120
100
80
60
40
20
0
1
2
3
4
Untreated (68)
Treated (62)
Patient groups
EL1
EL2
EL3
EL4
c
Zscan4 transcript
^
**
Log2 fold change
4
3
2
1
0
Untreated
Treated
Untreated
Treated
Epithelium
Stroma
d
***
Log2 fold change
5
4
3
2
1
0
Before
After
Sample groups
f
Zscan4
p38
mTOR
2
1
0
-1
-2
Zscan4
p38
mTOR
p16
e
Disease free survival (%)
100
90
80
70
60
50
40
30
20
10
0
Low Zscan4
High Zscan4
P < 0.01
(n = 27)
(n = 35)
0
10
20
30
40
50
DFI (months)
g
p38 scoring
Pearson r = 0.81
R2 = 0.65
Slope = 0.86
P < 0.01
Zscan4 scoring
h
mTOR scoring
Pearson r = 0.67
R2 = 0.44
Slope = 0.69
P < 0.01
Zscan4 scoring
i
Caspase 3 (cleaved) scoring
Pearson r = - 0.26
R2 = 0.07
Slope = - 0.24
P > 0.05
Zscan4 scoring

Supplementary Figure 2. Zscan4 is expressed in the stroma of human breast tumors after chemotherapy, and correlates with poor survival of patients. (**a**) Immunohistochemistry (IHC) assessment of Zscan4 protein in primary tumors of breast cancer (BCa) patients. Samples were from patients without chemotherapy experience (untreated) or with the experience of receiving genotoxic chemotherapy (post-chemotherapy). Tissues were subject to Zscan4-specific IHC and general hematoxylin-eosin (HE) staining. Arrows, intensive nuclear signals of Zscan4 in the stroma. Scale bars, 50 µm. Doxorubicin was used a chemotherapeutic agent for this treated patient. (**b**) Pathological appraisal of stromal Zscan4 expression in BCa patient tumors. Patients were individually assigned into 4 categories per IHC staining intensity. 1, negative; 2, weak; 3, moderate; 4, strong expression. Left, statistical comparison of the percentage of each category. Right, representative images of each category regarding Zscan4 signals. ES, expression level. Scale bars, 50 µm. $P < 0.05$ by two-way ANOVA. (**c**) Quantitative RT-PCR analysis of Zscan4 transcript expression with total RNAs collected upon laser capture microdissection of BCa patient tumor and stroma, respectively. Signals were normalized to the lowest value in the untreated epithelium group. Comparison was performed between treated and untreated samples per cell group. Data analyzed using Student's *t*-test. (**d**) Comparative analysis of Zscan4 expression at transcription level between stromal cells collected before and after chemotherapy. Each dot represents an individual patient, with the data of "before" and "after" connected to allow direct assessment of Zscan4 induction in the same patient. Data analyzed using Student's *t*-test. (**e**) Kaplan-Meier analysis of the disease free survival (DFS) duration of BCa patients stratified according to Zscan4 expression (low, average score < 2, green line, n = 27; high, average score ≥ 2, red line, n =

35). DFS represents the length (months) of period calculated from the date of BCa diagnosis to the point of first time disease relapse. Survival curves calculated according to the Kaplan–Meier method, with data compared with log-rank (Mantel-Cox) test. (**f**) Pathological association between Zscan4, p38 and mTOR in the stroma of BCa patients post-treatment. Scores were from the assessment of molecule-specific IHC staining, with expression levels colored to reflect low (blue) via modest (yellow) to high (red) protein abundance. Columns represent individual patients, rows individual molecules. Totally 62 patients treated by chemotherapy were analyzed, with scores of each patient averaged from 3 independent pathological readings per tumor. Bottom, representative IHC images of Zscan4, p38 (T-180) and mTOR (S-2448), respectively. Images are representative of tissue sections from 10 randomly selected patients who displayed relatively high Zscan4 expression. $P < 0.0001$ for score-score matching by two-way ANOVA. (**g**) Overall correlation between Zscan4 and p38 scores (Pearson analysis, r = 0.81; $P < 0.0001$) in the 62 tumors with matching protein expression data. Data analyzed using Student's *t*-test. (**h**) Overall correlation between Zscan4 and mTOR scores (Pearson analysis, r = 0.67; $P < 0.0001$) in the same group of tumors assessed in (**f**). Data analyzed using Student's *t*-test. (**i**) Statistical correlation between Zscan4 and caspase 3 (cleaved) scores (Pearson analysis, r = - 0.26; $P > 0.05$) in the same group of tumours described in (**f**). Data analyzed using Student's *t*-test. Data in bar plots are shown as mean ± SD and representative of 3 biological replicates. * $P < 0.05$, ** $P < 0.01$, *** $P < 0.001$, ^ $P > 0.05$.

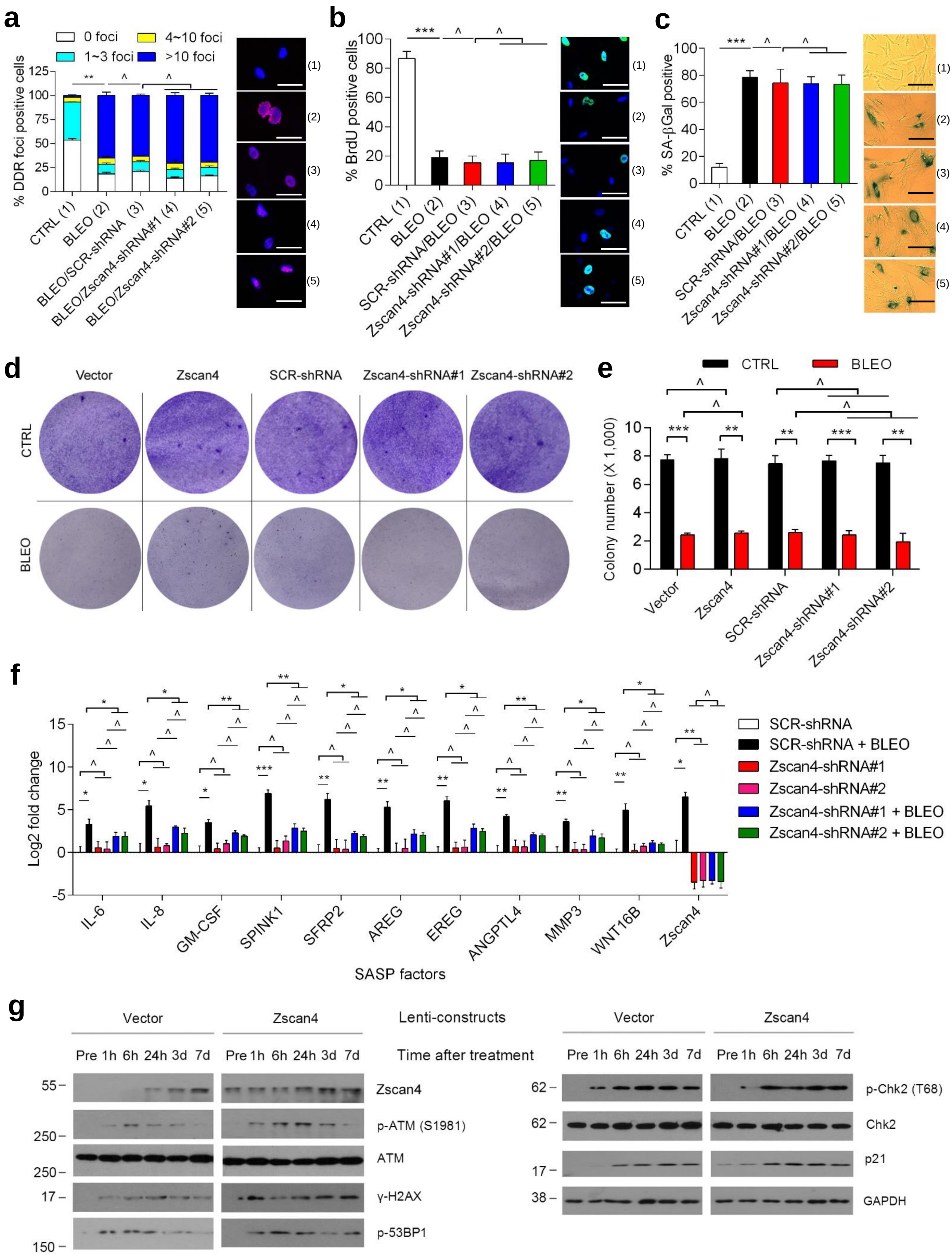
a
0 foci
4~10 foci
1~3 foci
>10 foci
% DDR foci positive cells
CTRL (1)
BLEO (2)
BLEO/SCR-shRNA (3)
BLEO/Zscan4-shRNA#1 (4)
BLEO/Zscan4-shRNA#2 (5)
b
% BrdU positive cells
CTRL (1)
BLEO (2)
SCR-shRNA/BLEO (3)
Zscan4-shRNA#1/BLEO (4)
Zscan4-shRNA#2/BLEO (5)
c
% SA-βGal positive
CTRL (1)
BLEO (2)
SCR-shRNA/BLEO (3)
Zscan4-shRNA#1/BLEO (4)
Zscan4-shRNA#2/BLEO (5)
d
Vector
Zscan4
SCR-shRNA
Zscan4-shRNA#1
Zscan4-shRNA#2
CTRL
BLEO
e
CTRL
BLEO
Colony number (X 1,000)
Vector
Zscan4
SCR-shRNA
Zscan4-shRNA#1
Zscan4-shRNA#2
f
Log2 fold change
SCR-shRNA
SCR-shRNA + BLEO
Zscan4-shRNA#1
Zscan4-shRNA#2
Zscan4-shRNA#1 + BLEO
Zscan4-shRNA#2 + BLEO
IL-6
IL-8
GM-CSF
SPINK1
SFRP2
AREG
EREG
ANGPTL4
MMP3
WNT16B
Zscan4
SASP factors
g
Vector
Zscan4
Lenti-constructs
Time after treatment
Pre 1h 6h 24h 3d 7d
Zscan4
p-ATM (S1981)
ATM
γ-H2AX
p-53BP1
p-Chk2 (T68)
Chk2
p21
GAPDH

Supplementary Figure 3. Influence of Zscan4 knockdown on DNA damage response (DDR), DNA synthesis, cellular senescence, colony formation, and expression of canonical SASP factors, and effect of Zscan4 overexpression on DDR signaling. (**a**) Statistics of DDR foci (DDF) in PSC27 stable sublines established via lentiviral infection with constructs that encode scramble shRNA (SCR-shRNA) or gene-specific shRNA for Zscan4 (Zscan4-shRNA#1 or Zscan4-shRNA#2), then subject to bleomycin treatment (50 µg/ml). The number of DDR foci per cell was counted and calculated into percentage of overall cell populations examined. Right, representative images (red, γH2AX; blue, DAPI), with the numeric numbers corresponding to those provided in the left graph. At least 200 cells counted per sample. Scale bars, 15 µm. Data analyzed using two-way ANOVA. (**b**) DNA synthesis assessment by BrdU incorporation with the number of BrdU-positive cells counted and calculated into percentage. Right, representative images (green, BrdU; blue, DAPI), with the numeric numbers corresponding to those provided in the left graph. At least 200 cells counted per sample. Scale bars, 15 µm. Data analyzed using Student's *t*-test. (**c**) Cellular senescence evaluation by SA-β-Gal staining of above cell sublines, with the percentage of positively stained cells shown in percentage. Right, representative images. At least 200 cells counted per sample. Scale bars, 20 µm. Data analyzed using Student's *t*-test. (**d**) Representative images of *in vitro* clonogenic assay in culture dishes performed with cell sublines tested above, either untreated or treated with bleomycin. (**e**) Statistics for cell-derived colonies grown in each of Zscan4-overxpression or Zscan4-knockdown groups. The colony numbers were counted and graphed. (**f**) PSC27 stable sublines established via lentiviral infection with scramble RNA (SCR-shRNA) or Zscan4 shRNAs (Zscan4-shRNA#1 or #2) construct, were treated by

bleomycin. Seven days post-treatment, cells were lysed for qRT-PCR analysis of SASP factor expression, with values normalized to SCR-shRNA per factor. Data analyzed using Student's *t*-test. (**g**) Immunoblot examination of Zscan4 and a subset of typical DDR factors (ATM, H2AX, 53BP1, Chk2) and p21 in PSC27 cells infected with lentivirus carrying either an empty vector or the Zscan4 construct. Cells were treated by bleomycin, with lysates collected at the individual time points after genotoxic treatment. GAPDH, loading control. Data in bar graphs are shown as mean ± SD and representative of 3 biological replicates. *$P < 0.05$, **$P < 0.01$, ***$P < 0.001$, ^$P > 0.05$. Images are representative of 3 independent experiments. CTRL, control. BLEO, bleomycin.

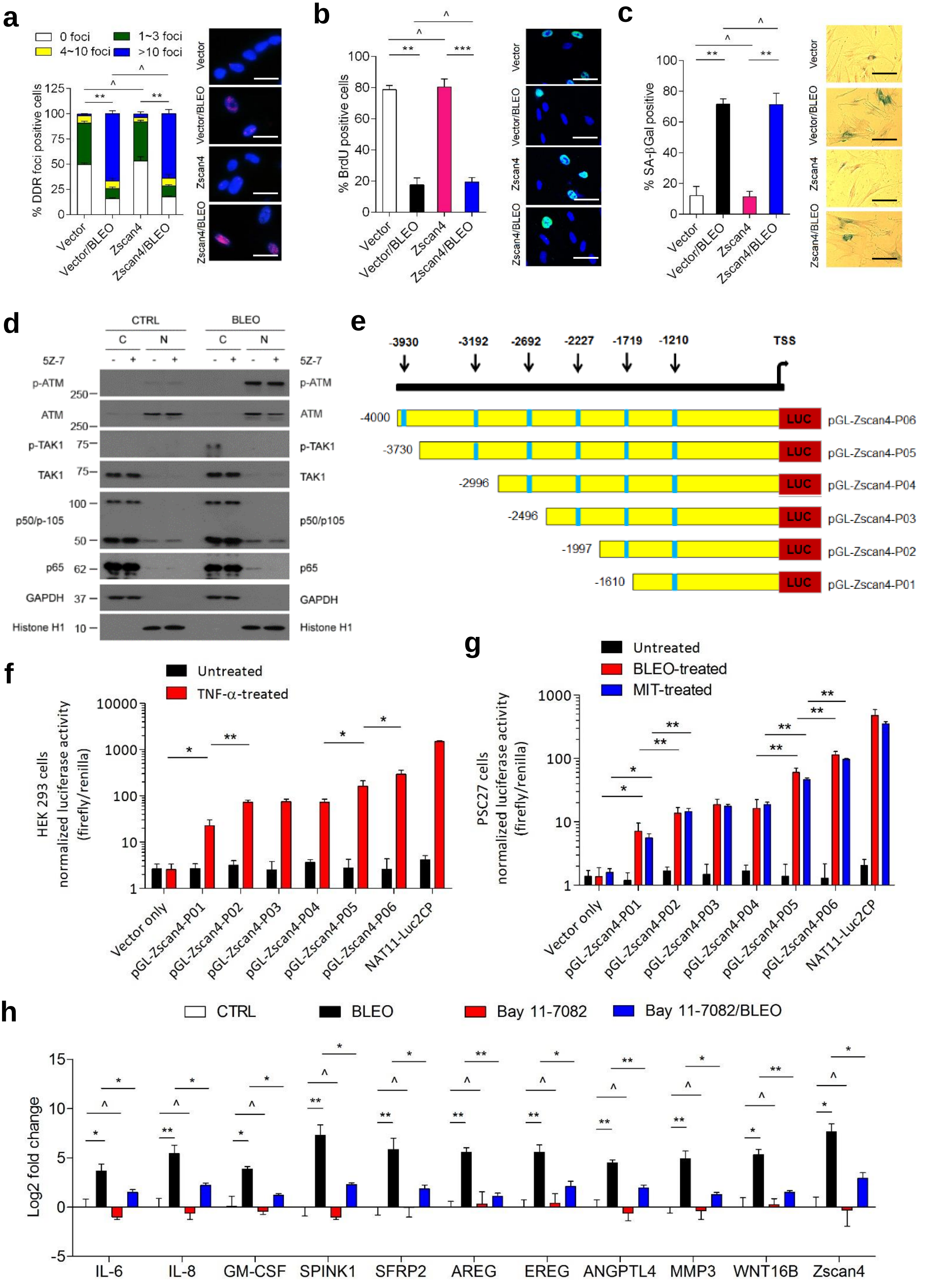
a
0 foci
1~3 foci
4~10 foci
>10 foci
% DDR foci positive cells
Vector
Vector/BLEO
Zscan4
Zscan4/BLEO
b
% BrdU positive cells
c
% SA-β-Gal positive
d
CTRL
BLEO
C
N
5Z-7
p-ATM
ATM
p-TAK1
TAK1
p50/p-105
p50/p105
p65
GAPDH
Histone H1
e
-3930
-3192
-2692
-2227
-1719
-1210
TSS
-4000
-3730
-2996
-2496
-1997
-1610
LUC
pGL-Zscan4-P06
pGL-Zscan4-P05
pGL-Zscan4-P04
pGL-Zscan4-P03
pGL-Zscan4-P02
pGL-Zscan4-P01
f
Untreated
TNF-α-treated
HEK 293 cells
normalized luciferase activity
(firefly/renilla)
Vector only
NAT11-Luc2CP
g
Untreated
BLEO-treated
MIT-treated
PSC27 cells
h
CTRL
BLEO
Bay 11-7082
Bay 11-7082/BLEO
Log2 fold change
IL-6
IL-8
GM-CSF
SPINK1
SFRP2
AREG
EREG
ANGPTL4
MMP3
WNT16B
Zscan4

Supplementary Figure 4. Influence of Zscan4 overexpression on breast stromal cell phenotypes and regulation of genotoxicity-induced Zscan4 expression by the NF-κB complex. (**a**) Statistics of DDR foci in HBF1203 stable sublines established via lentiviral infection and treated by bleomycin (50 ug/ml). The number of cells displaying each category of DDR foci was counted and converted into percentage per cell population. Right, representative images (red, γH2AX; blue, DAPI). At least 200 cells counted per sample. Scale bars, 15 µm. Data analyzed using two-way ANOVA. (**b**) DNA synthesis evaluation by BrdU incorporation of cell sublines assayed in (a), with BrdU-positive cells calculated in percentage. Right, representative images (green, BrdU; blue, DAPI). At least 200 cells counted per sample. Scale bars, 15 µm. (**c**) Cellular senescence assessment by SA-β-Gal staining of above cell sublines, with positively stained cells shown in percentage. Right, representative images. At least 200 cells counted per sample. Scale bars, 20 µm. (**d**) Cytoplasmic and nuclear protein lysates prepared from control and doxorubicin-treated HBF1203 cells were assessed for TAK1 activation and NF-κB nuclear translocation. Cells treated with or without 5Z-7 to suppress TAK1 were analyzed in parallel. GAPDH and Histone H1, cytoplasmic and nuclear loading controls, respectively. (**e**) Schematic representation of putative NF-κB binding sites in the proximal region of human Zscan4 promoter. A set of reporter constructs was generated by sequential cloning of the promoter fragments into a pGL4.22 vector that allows expression of firefly luciferase driven by the exogenous promoters (pGL-Zscan4-P01 to P06). Arrow heads on the top denotes the core site of each putative NF-κB binding motif. Numeric numbers at the left marks the length of each segmental promoter clone. TSS. Transcription start site. (**f**) Measurement of luciferase activities upon exposure of HEK 293 cells pre-transfected with

Zscan4 promoter expression constructs to TNF-α at 20 ng/ml in culture. The empty vector was used as a negative control, while the construct NAT11-Luc2CP encoding multiple copies of typical NF-κB binding sequences and an optimized IL-2 minimal promoter served as a positive control. (**g**) Luciferase activity assay with PSC27 cells that were transfected with each of the constructs used in (**f**) prior to treatment by 50 μg/ml bleomycin (BLEO) or 1 μM mitoxantrone (MIT). (**h**) Expression analysis of canonical SASP factors upon treatment by 50 μg/ml BLEO, 5 μM Bay 11-7082, or both. Cells were lysed 7 days post treatment. Signals were normalized to untreated (CTRL) samples per factor set. Data in bar plots are shown as mean ± SD and representative of 3 biological replicates. *$P < 0.05$, **$P < 0.01$, ^$P > 0.05$. Data in **b, c, f, g** and **h** were analyzed using Student's *t*-test.

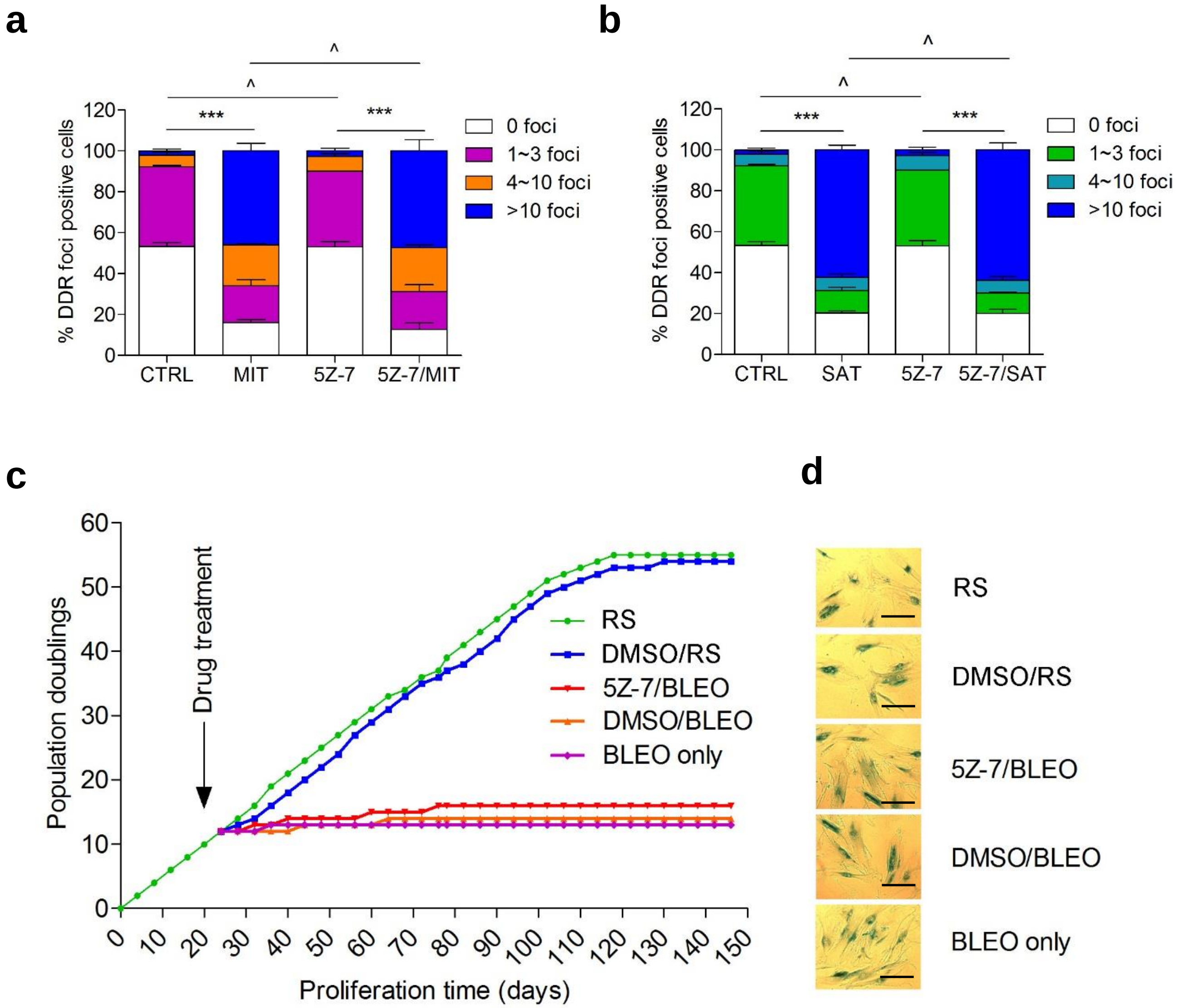
a
b
c
d
% DDR foci positive cells
0 foci
1~3 foci
4~10 foci
>10 foci
CTRL
MIT
5Z-7
5Z-7/MIT
SAT
5Z-7/SAT
***
^
Population doublings
Drug treatment
Proliferation time (days)
RS
DMSO/RS
5Z-7/BLEO
DMSO/BLEO
BLEO only

Supplementary Figure 5. TAK1 inhibition does not change the DNA damage extent and proliferative potential of stromal cells exposed to genotoxicity. (**a**) Statistics of DDF in PSC27 cells upon treatment by mitoxantrone (MIT, 1 μM), in the presence or absence of the TAK1 inhibitor 5Z-7-Oxozeaenol (5Z-7, 500 nM). (**b**) Statistics of DDR foci for stromal cells upon treatment by satraplatin (SAT, 10 μM), in the presence or absence of 5Z-7. (**c**) Population doubling-based proliferative assay of stromal cells under different treatment conditions. RS, replicative senescence. Proliferation time was counted in days. (**d**) Representative images of SA-β-Gal staining of cells as assayed in (**c**). Data in bar plots are shown as mean ± SD. All data are representative of 3 biological replicates. *$P < 0.05$, ***$P < 0.001$, ^$P > 0.05$. Data in **a** and **b** were analyzed using two-way ANOVA.

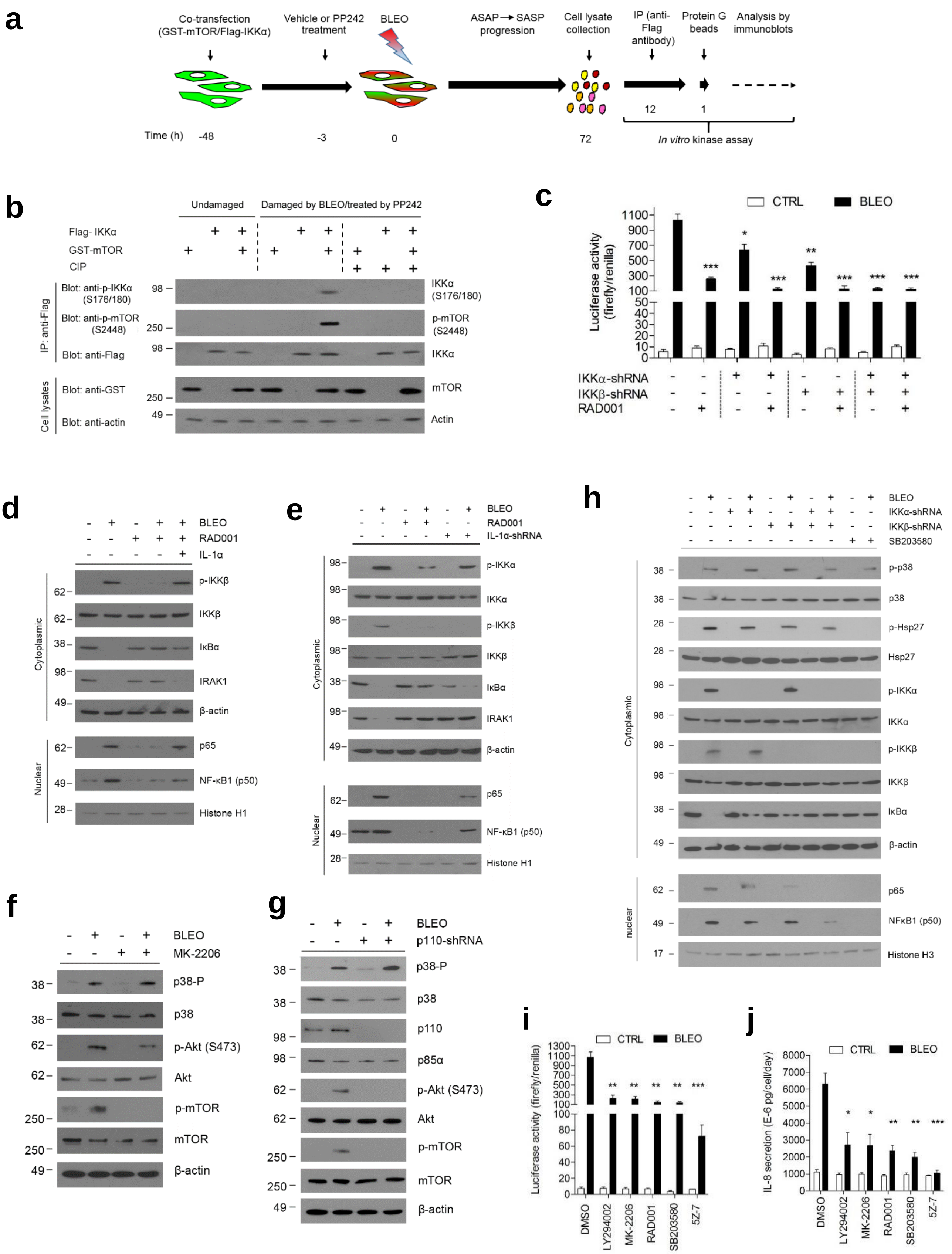
a
Co-transfection (GST-mTOR/Flag-IKKα)
Vehicle or PP242 treatment
BLEO
ASAP → SASP progression
Cell lysate collection
IP (anti-Flag antibody)
Protein G beads
Analysis by immunoblots
Time (h) -48 -3 0 72
12 1
In vitro kinase assay
b
Undamaged
Damaged by BLEO/treated by PP242
Flag- IKKα
GST-mTOR
CIP
IP: anti-Flag
Blot: anti-p-IKKα (S176/180)
Blot: anti-p-mTOR (S2448)
Blot: anti-Flag
Cell lysates
Blot: anti-GST
Blot: anti-actin
IKKα (S176/180)
p-mTOR (S2448)
IKKα
mTOR
Actin
c
CTRL
BLEO
Luciferase activity (firefly/renilla)
IKKα-shRNA
IKKβ-shRNA
RAD001
d
BLEO
RAD001
IL-1α
Cytoplasmic
Nuclear
p-IKKβ
IKKβ
IκBα
IRAK1
β-actin
p65
NF-κB1 (p50)
Histone H1
e
BLEO
RAD001
IL-1α-shRNA
Cytoplasmic
Nuclear
p-IKKα
IKKα
p-IKKβ
IKKβ
IκBα
IRAK1
β-actin
p65
NF-κB1 (p50)
Histone H1
h
BLEO
IKKα-shRNA
IKKβ-shRNA
SB203580
Cytoplasmic
nuclear
p-p38
p38
p-Hsp27
Hsp27
p-IKKα
IKKα
p-IKKβ
IKKβ
IκBα
β-actin
p65
NFκB1 (p50)
Histone H3
f
BLEO
MK-2206
p38-P
p38
p-Akt (S473)
Akt
p-mTOR
mTOR
β-actin
g
BLEO
p110-shRNA
p38-P
p38
p110
p85α
p-Akt (S473)
Akt
p-mTOR
mTOR
β-actin
i
CTRL
BLEO
Luciferase activity (firefly/renilla)
DMSO
LY294002
MK-2206
RAD001
SB203580
5Z-7
j
CTRL
BLEO
IL-8 secretion (E-6 pg/cell/day)
DMSO
LY294002
MK-2206
RAD001
SB203580
5Z-7

Supplementary Figure 6. Interaction of mTOR with IKK complex, IL-1α/NF-κB positive feedback loop and p38/PI3K/Akt pathways are implicated in cytoplasmic signaling to support the chronic SASP development. (**a**) Illustrative workflow of cell treatments in culture and subsequent co-IP experiments for *in vitro* kinase assay. (**b**) Identification of IKKα as a substrate of mTOR-mediated phosphorylation through *in vitro* kinase assay. PSC27 cells were transfected with a Flag-IKKα and/or GST-mTOR construct before genotoxic damage, and lysates were processed by IP followed by immunoblot analysis. CIP, calf intestinal phosphatase. Actin, loading control. (**c**) Reporter assay of NF-κB activity in stromal cells upon genetic manipulation by shRNA-mediated knockdown of IKK subunits or pharmacological treatment with RAD001 followed by genotoxic stress. (**d**) Exogenous IL-1α (20 pg/ml) rescues the RAD001-suppressed SASP under genotoxic conditions. After exposure of PSC27 cells to bleomycin, phosphorylation of IKKβ, cytoplasmic amounts of IKBα and IRAK1, and nuclear translocation of NK-κB subunits (p65 and p50) were determined by immunoblots. (**e**) Elimination of IL-1α from stromal cells does not abrogate SASP induced by DNA damage. In contrast to the conditions of (**d**), IL-1α was eliminated from PSC27 cells by shRNA prior to bleomycin treatment, with lysates analyzed for phosphorylation of IKKα and IKKβ, cytoplasmic preservation of IKBα and IRAK1, and NK-κB nuclear transportation. (**f**) Chemical treatment with MK-2206 (100 nM), a pan-Akt inhibitor, abrogated mTOR activation induced by genotoxic stress to stromal cells. Phosphorylation of p38, Akt and mTOR was examined by immunoblots. (**g**) Genetic elimination of p110, the catalytic subunit of PI3K by shRNA, dampened Akt/mTOR signaling in DNA-damaged stromal cells. Phosphorylation of p38, Akt and mTOR was examined. (**h**) Functional analysis of p38 in DNA-damaged stromal cells by examining its

correlation with NF-κB activation. PSC27 cells were either transduced with shRNA constructs against IKKα, IKKβ, or treated with SB203580 before DNA damage. Antibodies to phosphorylated and total p38, Hsp27, IKKα and IKKβ, total IKBα, and nuclear contents of the NF-κB subunits were used for immunoblots. (**i**) Reporter assay of NF-κB activity. The PI3K inhibitor LY294002 (1 μM), Akt inhibitor MK-2206 (100 nM), mTOR inhibitor RAD001 (50 nM), p38 inhibitor SB203580 (10 μM) and TAK1 inhibitor 5Z-7 (500 nM) are used to treat PSC27 cells prior to DNA damage. Luciferase signals were calculated by normalizing the readings of firefly/renilla. Statistical significance was relative to the value of undamaged cells. (**j**) Measurement of IL-8 production in the CM of stromal cells by ELISA assay. Cells were pre-treated with the small molecule inhibitors as in (**i**) before exposure to bleomycin in culture. Seven days post genotoxic stress, IL-8 concentration was measured in the unit of 10^{-6} pg/cell/day. Statistical significance relative to the control group. 5Z-7, 5*Z*-7-oxozeaenol. Data in bar plots are shown as mean ± SD. All date are representative of 3 biological replicates. *P < 0.05, **P < 0.01, ***P <0.001. Data in **c, i** and **j** were analyzed using Student's *t*-test.

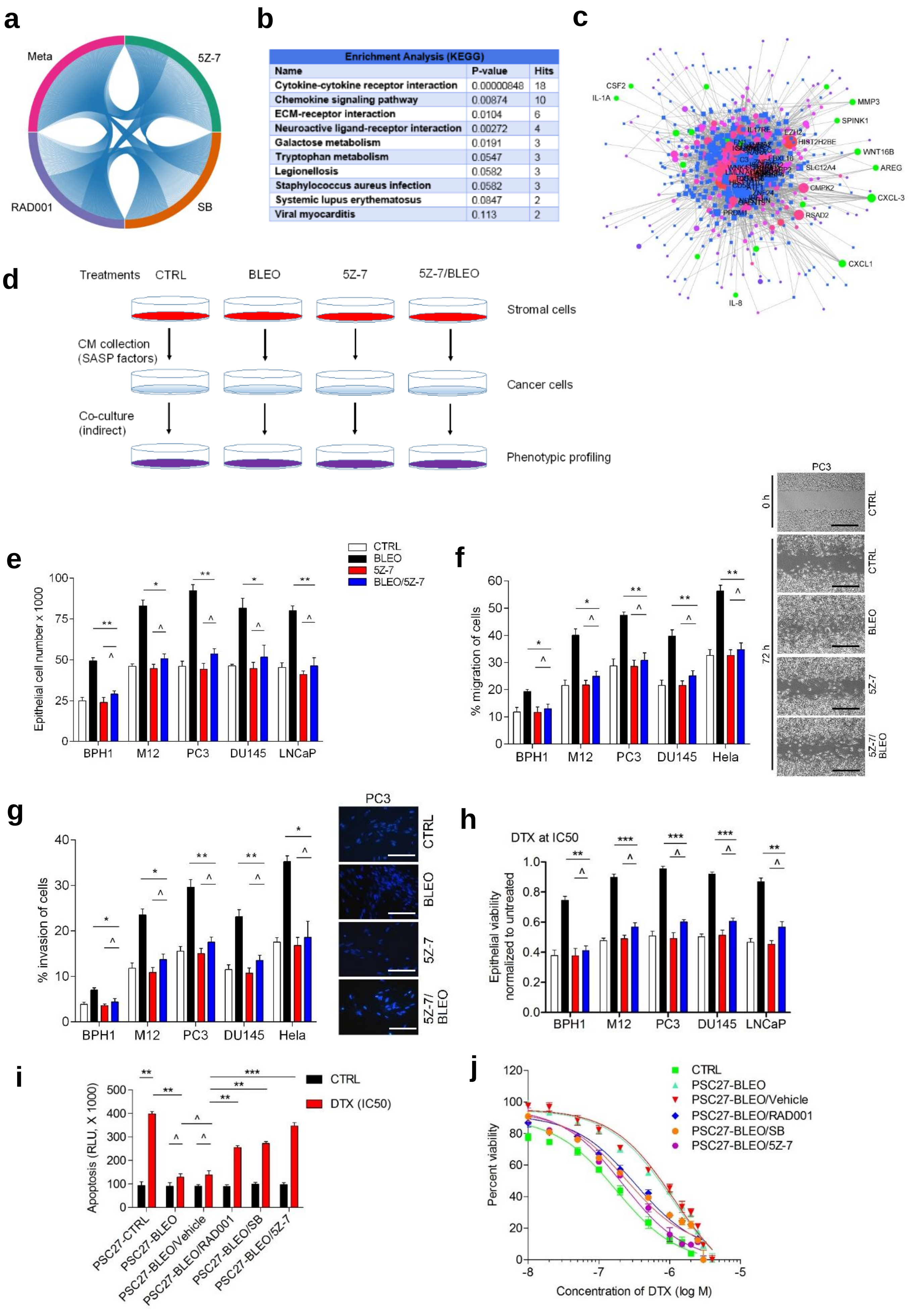
a
Meta
5Z-7
RAD001
SB
b
Enrichment Analysis (KEGG)
Name | P-value | Hits
Cytokine-cytokine receptor interaction | 0.00000848 | 18
Chemokine signaling pathway | 0.00874 | 10
ECM-receptor interaction | 0.0104 | 6
Neuroactive ligand-receptor interaction | 0.00272 | 4
Galactose metabolism | 0.0191 | 3
Tryptophan metabolism | 0.0547 | 3
Legionellosis | 0.0582 | 3
Staphylococcus aureus infection | 0.0582 | 3
Systemic lupus erythematosus | 0.0847 | 2
Viral myocarditis | 0.113 | 2
c
CSF2
IL-1A
MMP3
SPINK1
WNT16B
AREG
CXCL-3
CXCL1
IL-8
EZH2
HIST2H2BE
SLC12A4
CMPK2
RSAD2
PRDM1
d
Treatments CTRL BLEO 5Z-7 5Z-7/BLEO
Stromal cells
CM collection (SASP factors)
Cancer cells
Co-culture (indirect)
Phenotypic profiling
e
CTRL
BLEO
5Z-7
BLEO/5Z-7
Epithelial cell number x 1000
BPH1 M12 PC3 DU145 LNCaP
f
% migration of cells
BPH1 M12 PC3 DU145 Hela
PC3
0 h
72 h
CTRL
BLEO
5Z-7
5Z-7/BLEO
g
% invasion of cells
BPH1 M12 PC3 DU145 Hela
PC3
CTRL
BLEO
5Z-7
5Z-7/BLEO
h
DTX at IC50
Epithelial viability normalized to untreated
BPH1 M12 PC3 DU145 LNCaP
i
CTRL
DTX (IC50)
Apoptosis (RLU, X 1000)
PSC27-CTRL
PSC27-BLEO
PSC27-BLEO/Vehicle
PSC27-BLEO/RAD001
PSC27-BLEO/SB
PSC27-BLEO/5Z-7
j
CTRL
PSC27-BLEO
PSC27-BLEO/Vehicle
PSC27-BLEO/RAD001
PSC27-BLEO/SB
PSC27-BLEO/5Z-7
Percent viability
Concentration of DTX (log M)

Supplementary Figure 7. Comparative transcriptomic profiling of SASP-inhibited stromal cells and consequential influence on malignant phenotypes of cancer cells. (**a**) Chord diagram delineates the correlation of significantly downregulated genes (fold change > 2) by 5Z-7, SB or RAD001 in bleomycin-treated stromal cells. Meta, a meta-dataset combined from the raw datasets generated individually by 3 candidate SASP inhibitors for increased statistical power and cross study validation. (**b**) Summary of enrichment analysis by the KEGG pathway evaluation for top 809 genes whose expression was downregulated (fold change > 2) by 5Z-7 in bleomycin-treated cells. An IMEx interactome database was selected for protein-protein interaction assessment. (**c**) Construction of a nodal network from the top 809 genes described above. Green dots, canonical SASP factors. Connecting lines, putative protein-protein interactions. Representative SASP factors downregulated by 5Z-7 are shown to highlight the effect of TAK1 inhibition. Nodes, 580; edges, 4046; seeds, 252. (**d**) Workflow of indirect co-culture of stromal cells with cancer epithelial cells. Soluble factor-containing CM from stromal cells were collected after *in vitro* drug treatment and applied to cancer cells to for phenotype analysis. (**e**) Proliferation assay of an immortalized but benign epithelial cell line BPH1 and malignant cell lines M12, PC3, DU145 and LNCaP (all of prostate origin). Cells were cultured for 3 days with the CM from PSC27 cells treated in several conditions as indicated in (**a**). (**f**) Migration assay of cancer cells cultured for 3 days with different types of CM from stromal cells. BPH1, M12, PC3 and LNCaP lines were examined in parallel with HeLa line, which served as a positive control. Right, representative images of PC3 cell migration measured at 0 h and 72 h, respectively. Scale bars, 100 μm. (**g**) Invasiveness assay of cancer cell lines with collagen-based transwells in culture condition. Right, representative images of

PC3 cell invasion across the transwell membrane, followed by DAPI staining to locate invading cells. Scale bars, 50 μm. (**h**) Chemoresistance assay of prostate epithelial cells cultured with various types of CM from stromal cells. DTX was applied at the concentration of IC50 value pre-determined per cell line. DTX, docetaxel. (**i**) Apoptotic assay for combined activities of caspase 3/7 determined 24 h after exposure of PC3 cells to CM of stromal cells while being treated by DTX. RLU, relative luciferase unit. (**j**) Dose response curve (non-linear regression fit) by measuring the viability of PC3 cells across a range of MIT concentrations (10^{-8} to 10^{-5} M). Blue, orange and pink lines represent PC3 response to CM of PSC27 treated by bleomycin combined with RAD001, SB or 5Z-7, respectively. Data shown in bar graphs are mean ± SD. All data are representative of 3 independent experiments. SB, SB203580. 5Z-7, 5*Z*-7-oxozeaenol. Data in **e, f, g, h** and **i** were analyzed using Student's *t*-test.

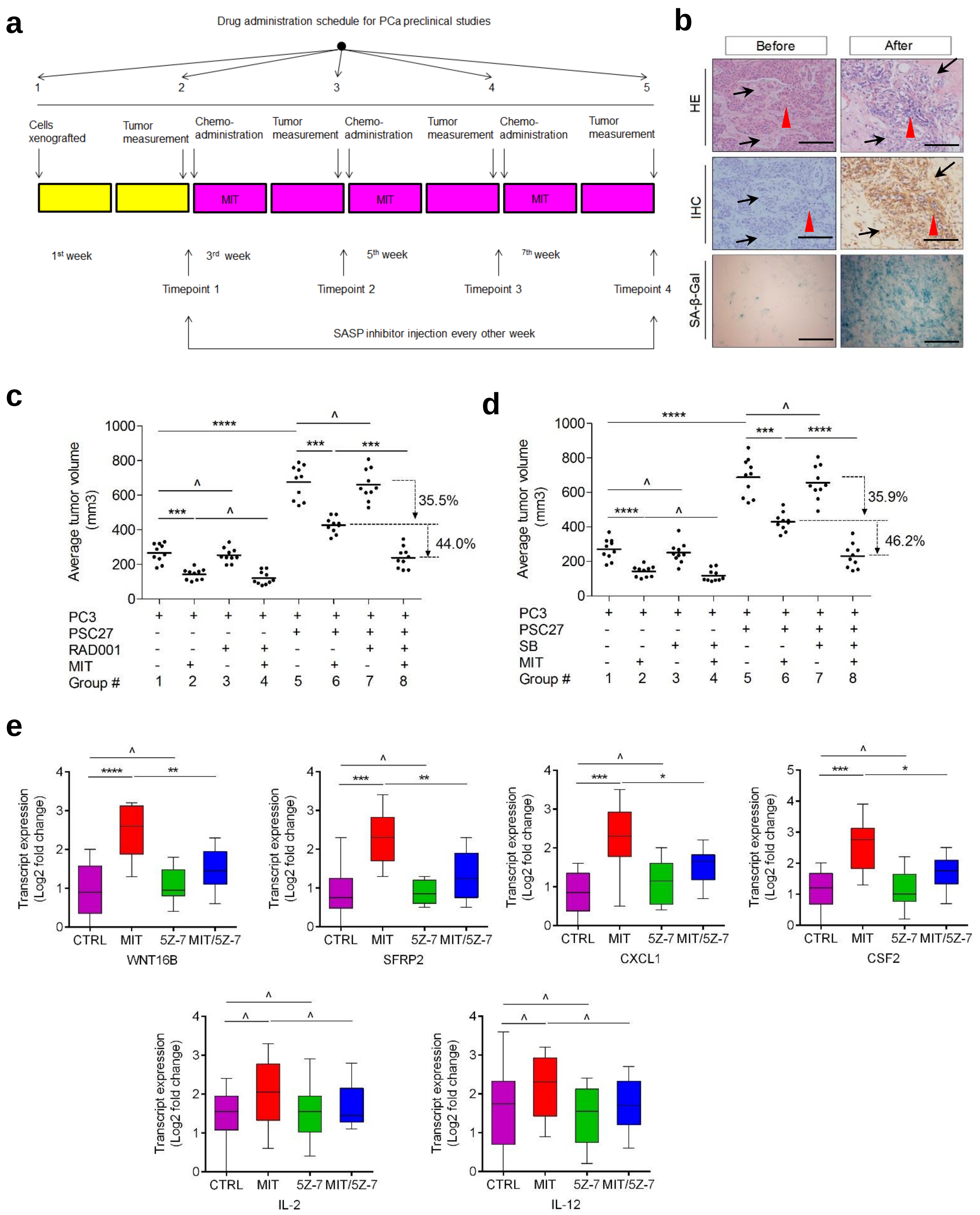
a
Drug administration schedule for PCa preclinical studies
1
2
3
4
5
Cells xenografted
Tumor measurement
Chemo-administration
Tumor measurement
Chemo-administration
Tumor measurement
Chemo-administration
Tumor measurement
MIT
MIT
MIT
1st week
3rd week
5th week
7th week
Timepoint 1
Timepoint 2
Timepoint 3
Timepoint 4
SASP inhibitor injection every other week
b
Before
After
HE
IHC
SA-β-Gal
c
Average tumor volume (mm3)
1000
800
600
400
200
0
****
^
***
***
^
***
^
35.5%
44.0%
PC3 + + + + + + + +
PSC27 - - - - + + + +
RAD001 - - + + - - + +
MIT - + - + - + - +
Group # 1 2 3 4 5 6 7 8
d
Average tumor volume (mm3)
1000
800
600
400
200
0
****
^
***
****
^
****
^
35.9%
46.2%
PC3 + + + + + + + +
PSC27 - - - - + + + +
SB - - + + - - + +
MIT - + - + - + - +
Group # 1 2 3 4 5 6 7 8
e
Transcript expression (Log2 fold change)
CTRL
MIT
5Z-7
MIT/5Z-7
WNT16B
^
****
**
SFRP2
^
***
**
CXCL1
^
***
*
CSF2
^
***
*
IL-2
^
^
^
IL-12
^
^
^

Supplementary Figure 8. Preclinical schedule, treatment-induced cellular senescence, tumor regression by combinational administration and SASP factor expression in mice carrying prostate tumors. (**a**) Drug administration scheme and tumor surveillance for preclinical investigation. PC3 cells along or admixed with PSC27 cells were inoculated subcutaneously to SCID mice 2 weeks prior to the scheduled MIT dosing, which was given on the first day of each week starting from the 3rd week, then given every other week with a total number of 3 doses. The SASP inhibitors (RAD001, SB203580 or 5Z-7) were given 12 h before each time of MIT delivery (totally 3 doses in the regimen). At the end of 8 weeks, mice were sacrificed with tumor volume measured, and histologically analyzed. (**b**) Representative images of *in vivo* cellular senescence caused by MIT-mediated chemotherapy. PC3/PSC27 tumors were acquired from experimental mice at the end of therapeutic regimen and subject to histological assessment. Images from staining of HE, IHC (anti-p16 as primary antibody) and SA-β-gal staining are provided for both before- and after-treatment samples for comparison. Black arrows, stromal cells. Red arrowheads, cancer cells. Scale bars, 150 μm. (**c**) Statistic quantification of endpoint volume of PC3 tumors subcutaneously implanted with or without stromal cells to SCID mice in the absence or presence of co-administered RAD001, followed by an 8-week chemotherapeutical regimen. (**d**) Similar quantification of endpoint volume of PC3 tumors described as above in (**c**), in the absence or presence of co-administered SB, in an 8-week treatment regimen. (**e**) Quantitative transcript expression analysis of representative canonical SASP factors expressed in stromal cells isolated from the tumours of SCID mice. Animals that had both stromal and cancer cells in the tumour foci were selected for examination. IL-2 and IL-12, factors not associated with the SASP but assayed as negative

controls. Data are shown as mean ± SD and representative of 3 independent experiments. MIT, mitoxantrone. SB, SB203580. 5Z-7, 5*Z*-7-oxozeaenol. N = 10 per treatment arm. *$P < 0.05$, **$P < 0.01$, ***$P < 0.001$, ****$P < 0.0001$, ^$P > 0.05$. Data in **c, d** and **e** were analyzed using Student's *t*-test.

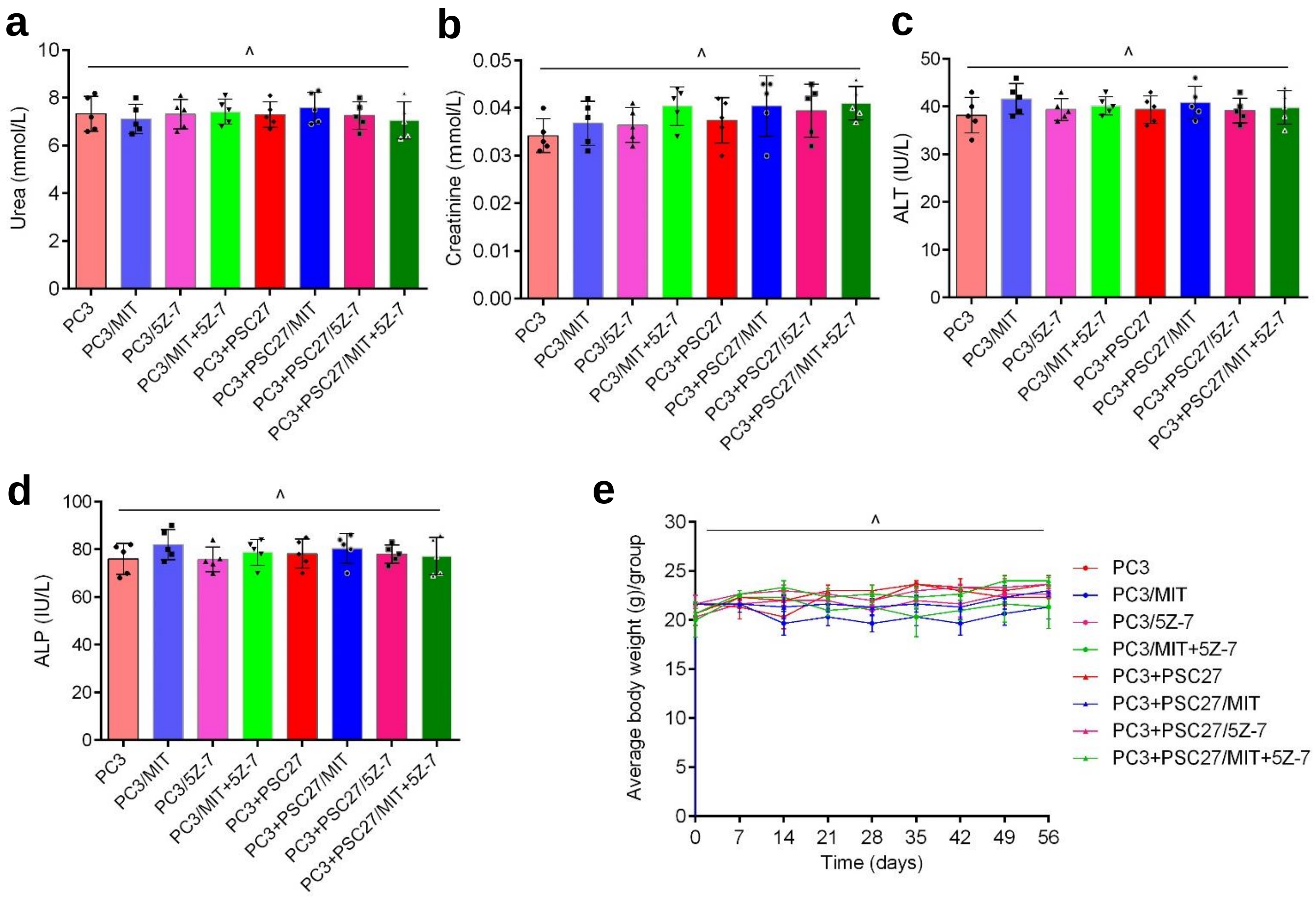

a
Urea (mmol/L)
0
2
4
6
8
10
^
PC3
PC3/MIT
PC3/5Z-7
PC3/MIT+5Z-7
PC3+PSC27
PC3+PSC27/MIT
PC3+PSC27/5Z-7
PC3+PSC27/MIT+5Z-7
b
Creatinine (mmol/L)
0.00
0.01
0.02
0.03
0.04
0.05
^
c
ALT (IU/L)
0
10
20
30
40
50
^
d
ALP (IU/L)
0
20
40
60
80
100
^
e
Average body weight (g)/group
0
5
10
15
20
25
30
^
Time (days)
0
7
14
21
28
35
42
49
56
PC3
PC3/MIT
PC3/5Z-7
PC3/MIT+5Z-7
PC3+PSC27
PC3+PSC27/MIT
PC3+PSC27/5Z-7
PC3+PSC27/MIT+5Z-7

Supplementary Figure 9. Appraisal of MIT and/or 5Z-7 treatment effects on mouse biochemistry and body weight. (**a**) Serum urea measurement with terminal bleeds (cardiac punctures) taken at the end of therapeutic regimens. Serum creatinine assessment with samples collected in (**a**). (**b**) Serum alanine aminotransferase (ALT) evaluation with samples of (**a**). (**c**) Serum alkaline phosphatase (ALP) appraisal with samples of (**a**). (**d**) Mouse body weight determination performed on a weekly basis. Data are shown as mean ± SD and representative of 3 independent experiments. MIT, mitoxantrone. 5Z-7, 5*Z*-7-oxozeaenol. N = 5 per treatment arm. ^$P > 0.05$. Data in **a-e** were analyzed using Student's *t*-test.

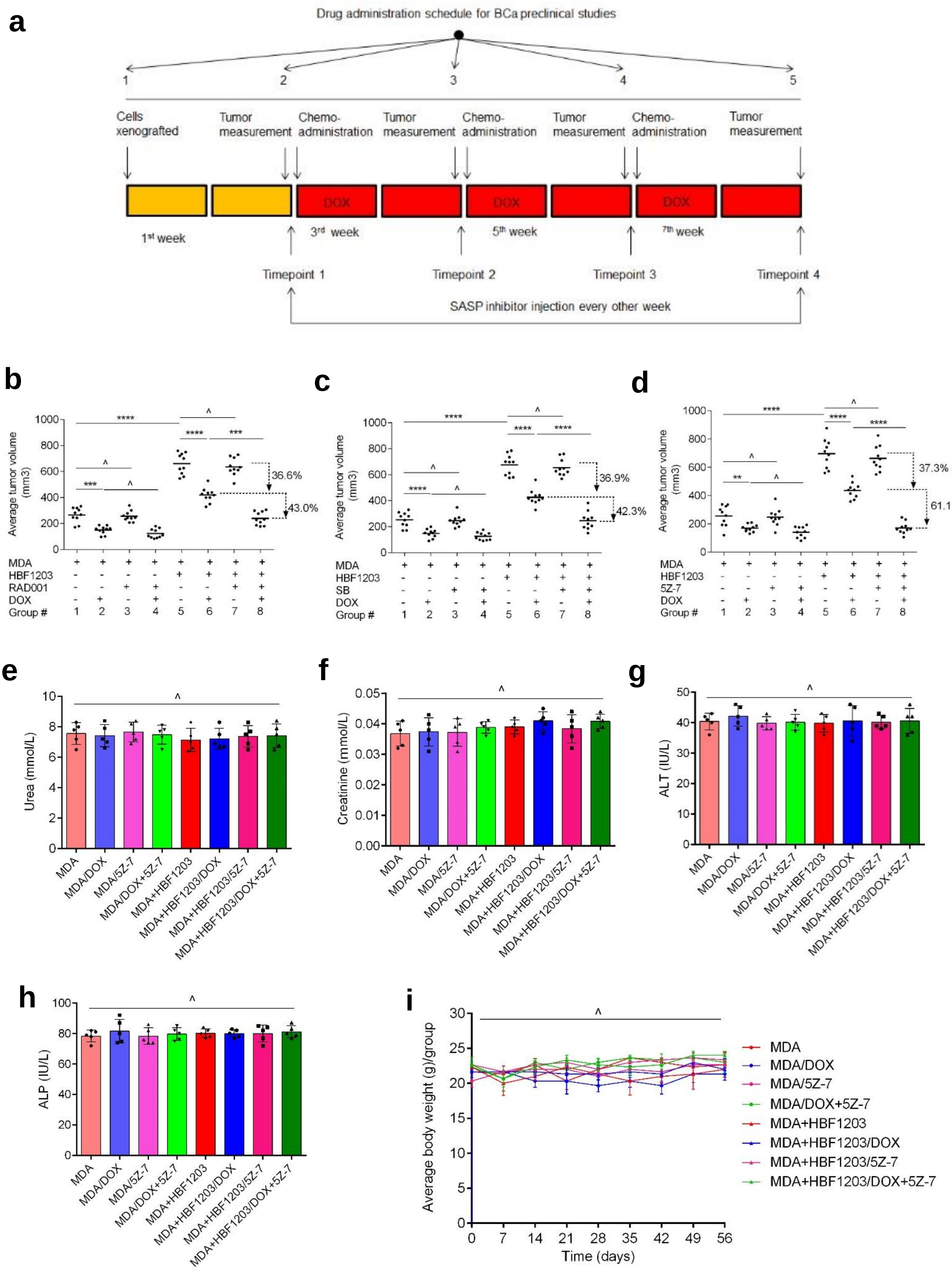
a
Drug administration schedule for BCa preclinical studies
Cells xenografted
Tumor measurement
Chemo-administration
DOX
1st week
3rd week
5th week
7th week
Timepoint 1
Timepoint 2
Timepoint 3
Timepoint 4
SASP inhibitor injection every other week
b
c
d
Average tumor volume (mm3)
MDA
HBF1203
RAD001
SB
5Z-7
DOX
Group #
36.6%
43.0%
36.9%
42.3%
37.3%
61.1%
e
f
g
h
i
Urea (mmol/L)
Creatinine (mmol/L)
ALT (IU/L)
ALP (IU/L)
Average body weight (g)/group
Time (days)
MDA
MDA/DOX
MDA/5Z-7
MDA/DOX+5Z-7
MDA+HBF1203
MDA+HBF1203/DOX
MDA+HBF1203/5Z-7
MDA+HBF1203/DOX+5Z-7

Supplementary Figure 10. Preclinical schedule, combinational administration, biochemistry and body weight measurement of animals carrying breast tumors. (**a**) Drug administration scheme and tumor surveillance for preclinical investigation. MDA-MB-231 cells along or admixed with HBF1203 cells were inoculated subcutaneously to SCID mice 2 weeks prior to the scheduled DOX dosing, which was given on the first day of each week starting from the 3rd week, then given every other week with a total number of 3 doses. The SASP inhibitors (RAD001, SB203580 or 5Z-7) were given 12 h before each time of DOX delivery (totally 3 doses in the regimen). At the end of 8 weeks, mice were sacrificed with tumor volume measured, and histologically analyzed. (**b**) Statistic quantification of endpoint volume of breast tumors subcutaneously implanted with or without stromal cells to SCID mice in the absence or presence of co-administered RAD001, followed by an 8-week chemotherapeutical regimen. (**c**) Statistic quantification of endpoint volume of breast tumors subcutaneously implanted with or without stromal cells to SCID mice in the absence or presence of co-administered SB, followed by an 8-week chemotherapeutical regimen. (**d**) Statistic quantification of endpoint volume of breast tumors subcutaneously implanted with or without stromal cells to SCID mice in the absence or presence of co-administered 5Z-7, followed by an 8-week chemotherapeutical regimen. (**e**) Serum urea measurement with terminal bleeds (cardiac punctures) taken at the end of therapeutic regimens. (**f**) Serum creatinine assessment with samples collected in (**e**). (**g**) Serum alanine aminotransferase (ALT) evaluation with samples of (**e**). (**h**) Serum alkaline phosphatase (ALP) appraisal with samples of (**e**). (**i**) Mouse body weight determination performed on a weekly basis. Data are shown as mean ± SD and representative of 3 independent experiments. MDA, MDA-MB-231. DOX, doxorubicin. SB, SB203580. 5Z-7,

5*Z*-7-oxozeaenol. N = 10 per treatment arm for experiments b-d, and N = 5 per treatment arm for experiments e-i. $^{**}P < 0.01$, $^{***}P < 0.001$, $^{****}P < 0.0001$, $^{\wedge}P > 0.05$. Data in **b-i** were analyzed using Student's *t*-test.

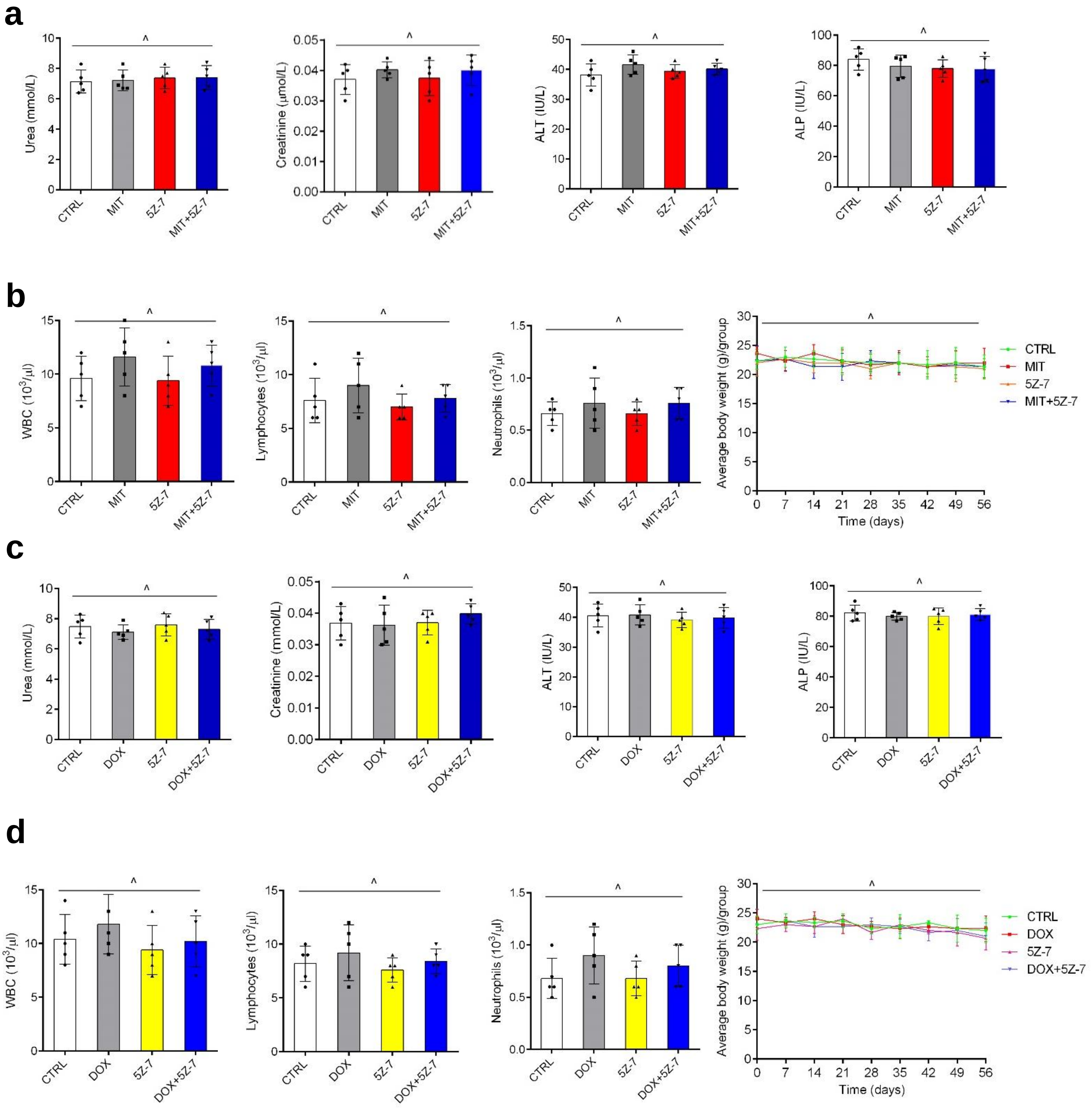
a
Urea (mmol/L)
Creatinine (μmol/L)
ALT (IU/L)
ALP (IU/L)
CTRL
MIT
5Z-7
MIT+5Z-7
^
b
WBC (10^3/μl)
Lymphocytes (10^3/μl)
Neutrophils (10^3/μl)
Average body weight (g)/group
Time (days)
CTRL
MIT
5Z-7
MIT+5Z-7
^
c
Urea (mmol/L)
Creatinine (mmol/L)
ALT (IU/L)
ALP (IU/L)
CTRL
DOX
5Z-7
DOX+5Z-7
^
d
WBC (10^3/μl)
Lymphocytes (10^3/μl)
Neutrophils (10^3/μl)
Average body weight (g)/group
Time (days)
CTRL
DOX
5Z-7
DOX+5Z-7
^

Supplementary Figure 11. Biochemistry, body weight measurement and regular blood counts of immune-competent animals (C57BL/6 mice) carrying prostate or breast tumors treated by chemotherapeutic agents. C57BL/6 mice were administered with MIT (or DOX) on a schedule that resembles those designed for drug treatment of animals carrying prostate or breast tumors, without a tumor uptake period of the first 2 weeks, as there was no tumor implantation performed for these immune-competent animals. (**a**) Serum urea, creatinine, alanine aminotransferase (ALT) and alkaline phosphatase (ALP) measurement with terminal bleeds (cardiac punctures) taken at the end of therapeutic regimens (6 weeks). MIT and 5Z-7 were administered either alone or together as dual agents. (**b**) WBC, lymphocyte and neutrophil counts were performed with mice examined in (**a**). Mouse body weights were determined once per week. (**c**) Serum urea, creatinine, alanine aminotransferase (ALT) and alkaline phosphatase (ALP) measurement with terminal bleeds (cardiac punctures) taken at the end of therapeutic regimens (6 weeks). DOX and 5Z-7 were administered either alone or together as dual agents. (**d**) WBC, lymphocyte and neutrophil counts were performed with mice examined in (**c**). Mouse body weights were determined once per week. Data are shown as mean ± SD and representative of 3 independent experiments. MIT, mitoxantrone. DOX, doxorubicin. 5Z-7, 5*Z*-7-oxozeaenol. WBC, white blood count. N = 5 per treatment arm. ^$P > 0.05$. Data in **a-d** were analyzed using Student's *t*-test.

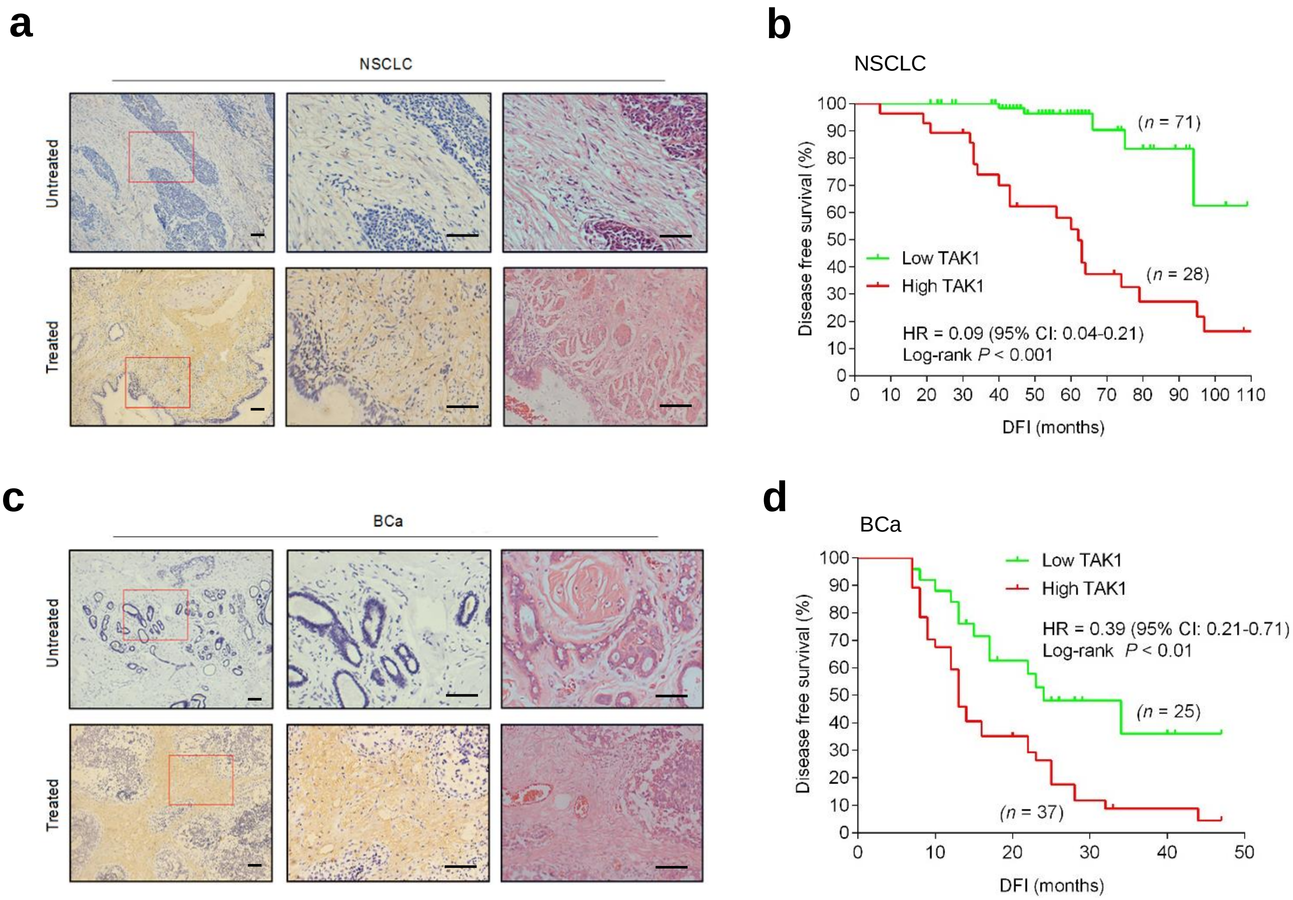
a
NSCLC
Untreated
Treated
b
NSCLC
Disease free survival (%)
(n = 71)
(n = 28)
Low TAK1
High TAK1
HR = 0.09 (95% CI: 0.04-0.21)
Log-rank P < 0.001
DFI (months)
c
BCa
Untreated
Treated
d
BCa
Disease free survival (%)
Low TAK1
High TAK1
HR = 0.39 (95% CI: 0.21-0.71)
Log-rank P < 0.01
(n = 25)
(n = 37)
DFI (months)

Supplementary Figure 12. Activation of TAK1 in the TME by genotoxic chemotherapy and its pathological correlation with disease free survival (DFS) of cancer patients in the post-treatment stage. (**a**) IHC and HE staining of phosphorylated TAK1 (p-TAK1) in the tumors of NSCLC patients after chemotherapeutic treatments. In each tumor type, red rectangular in the left IHC image is zoomed into the middle image, with an HE-staining image supplied on the right. Scale bars, 100 μm. (**b**) Kaplan-Meier analysis of NSCLC patients stratified according to TAK1 activation per p-TAK1 staining intensity (low, average score < 2, green line, n = 71; high, average score ≥ 2, red line, n = 28). DFS represents the length (months) of period calculated from the date of NSCLC diagnosis to the point of first time disease relapse. Survival curves calculated according to the Kaplan–Meier method, with $P < 0.05$ considered significant. (**c**) IHC and HE staining of phosphorylated TAK1 (p-TAK1) in the tumors of BCa patients after chemotherapeutic treatments. In each tumor type, red rectangular in the left IHC image is zoomed into the middle image, with an HE-staining image supplied on the right. Scale bars, 100 μm. (**d**) Survival analysis performed for BCa patients stratified according to TAK1 activation per p-TAK1 staining intensity (low, average score < 2, green line, n = 25; high, average score ≥ 2, red line, n = 37). Data in **b** and **d** were compared with log-rank (Mantel-Cox) test.

Fig. 1g

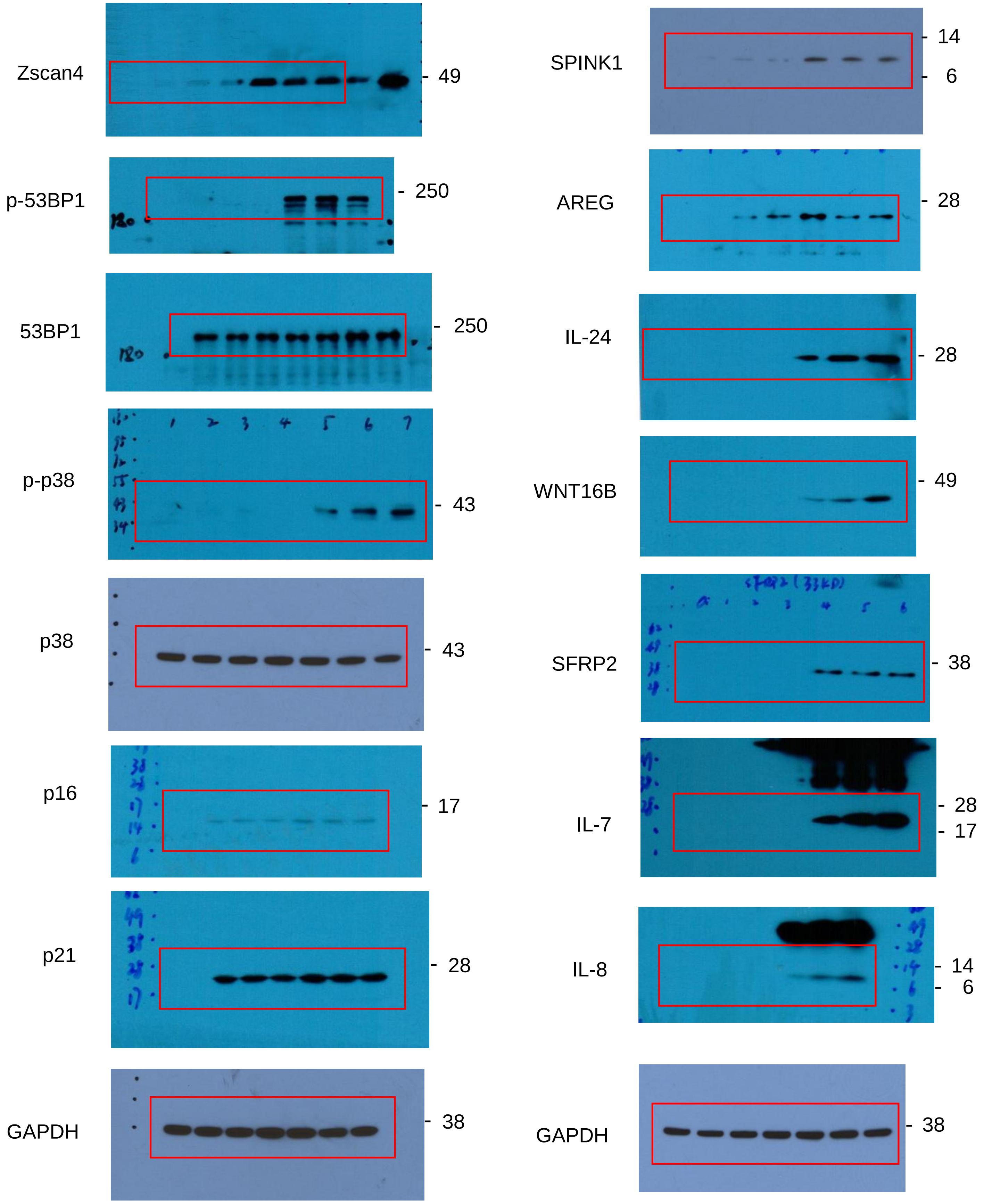

Supplementary Figure 13. The uncropped scans of immunoblots

Fig. 3e left

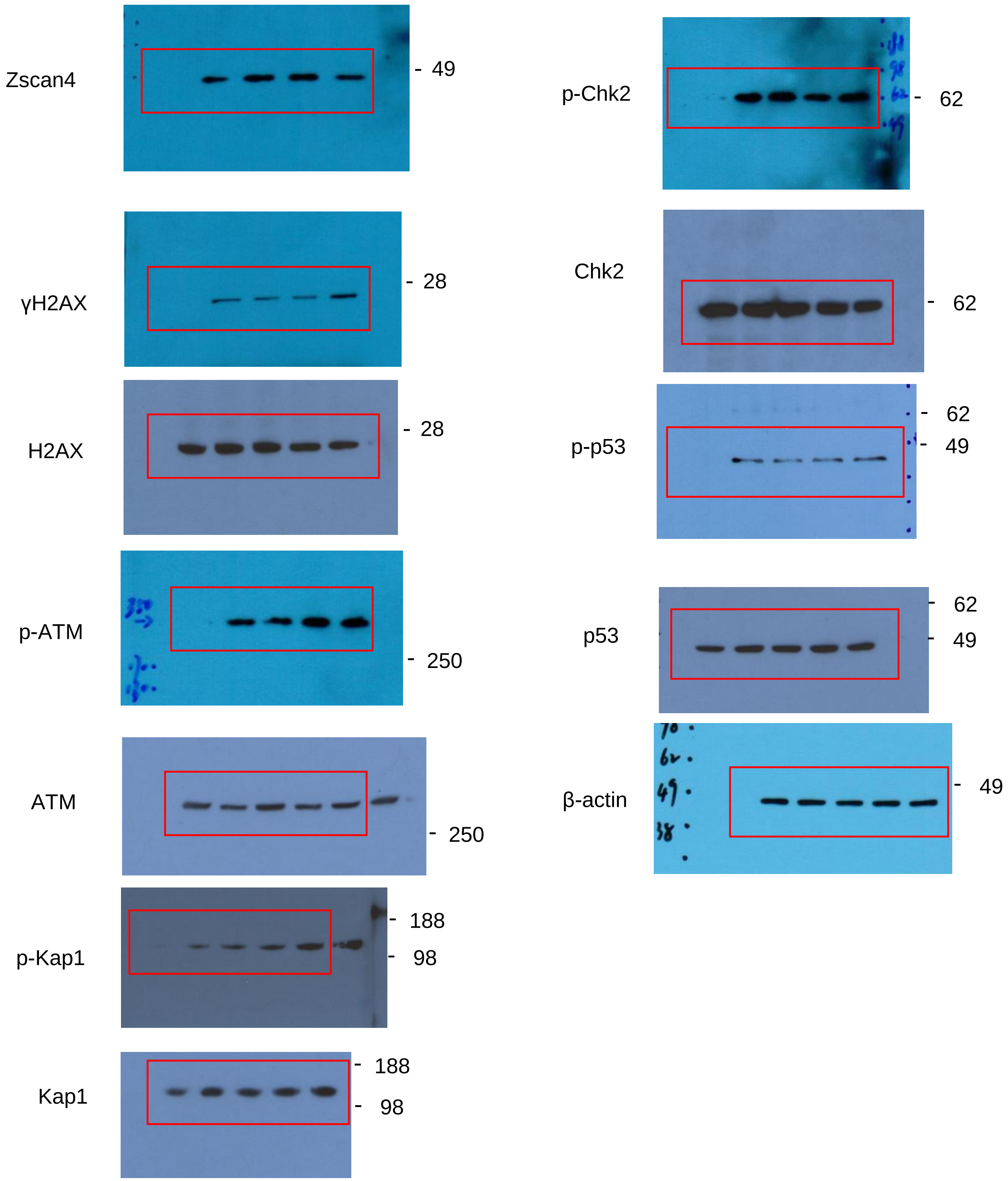

Supplementary Figure 13. The uncropped scans of immunoblots (continued)

Fig. 3e right

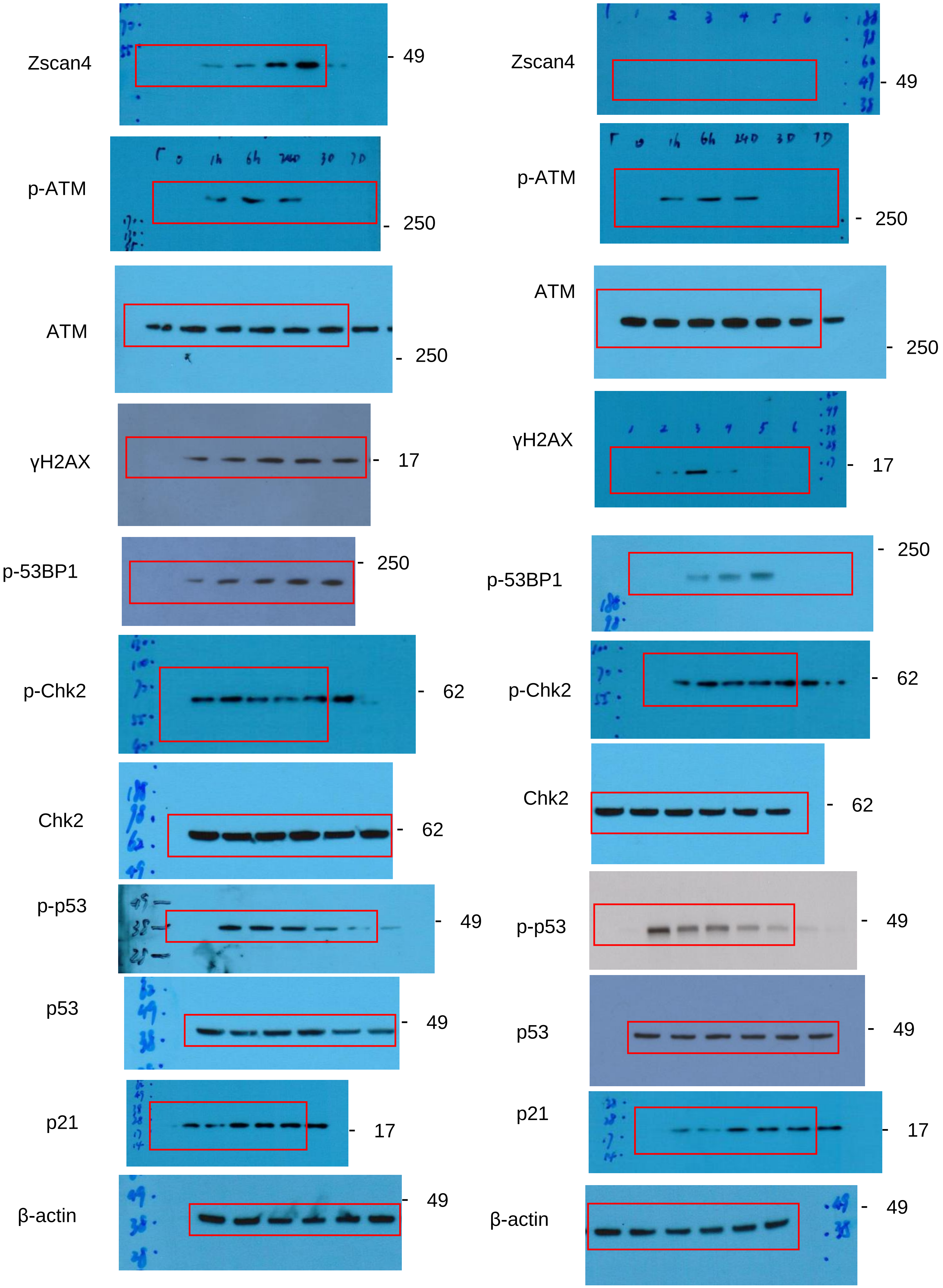

Supplementary Figure 13. The uncropped scans of immunoblots (continued)

Fig. 4a

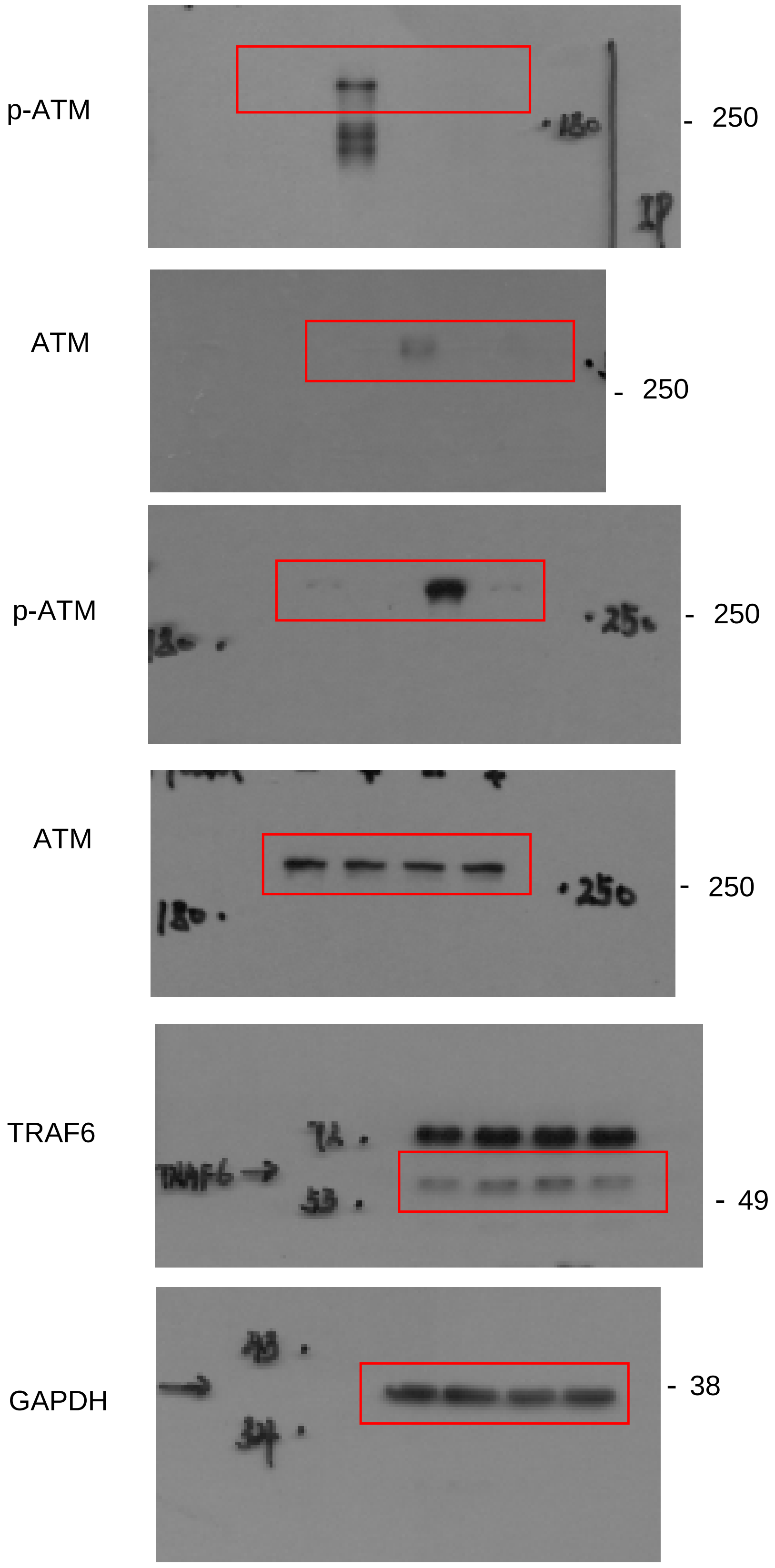

Supplementary Figure 13. The uncropped scans of immunoblots (continued)

Fig. 4b

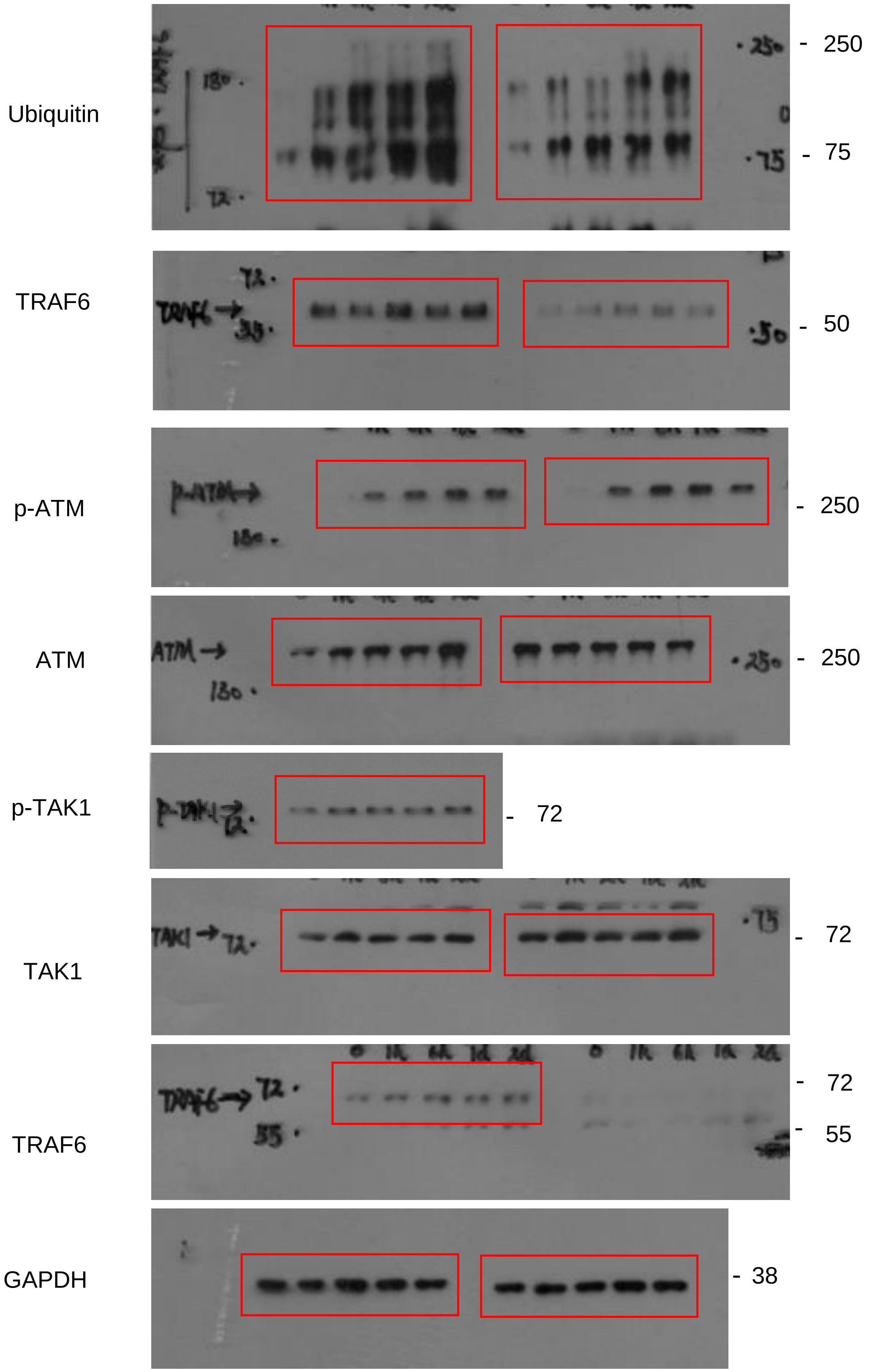

Supplementary Figure 13. The uncropped scans of immunoblots (continued)

Fig. 4c

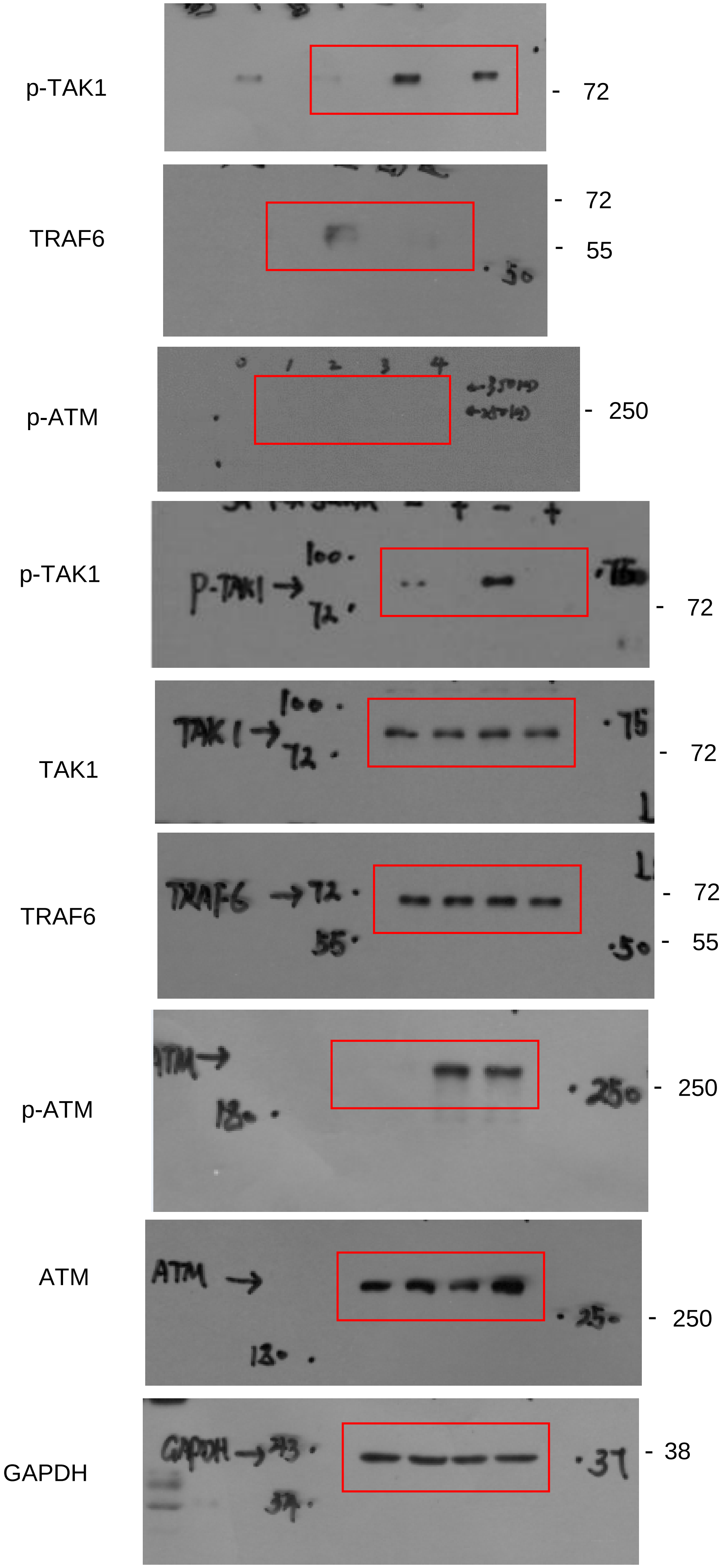

Supplementary Figure 13. The uncropped scans of immunoblots (continued)

Fig. 4d

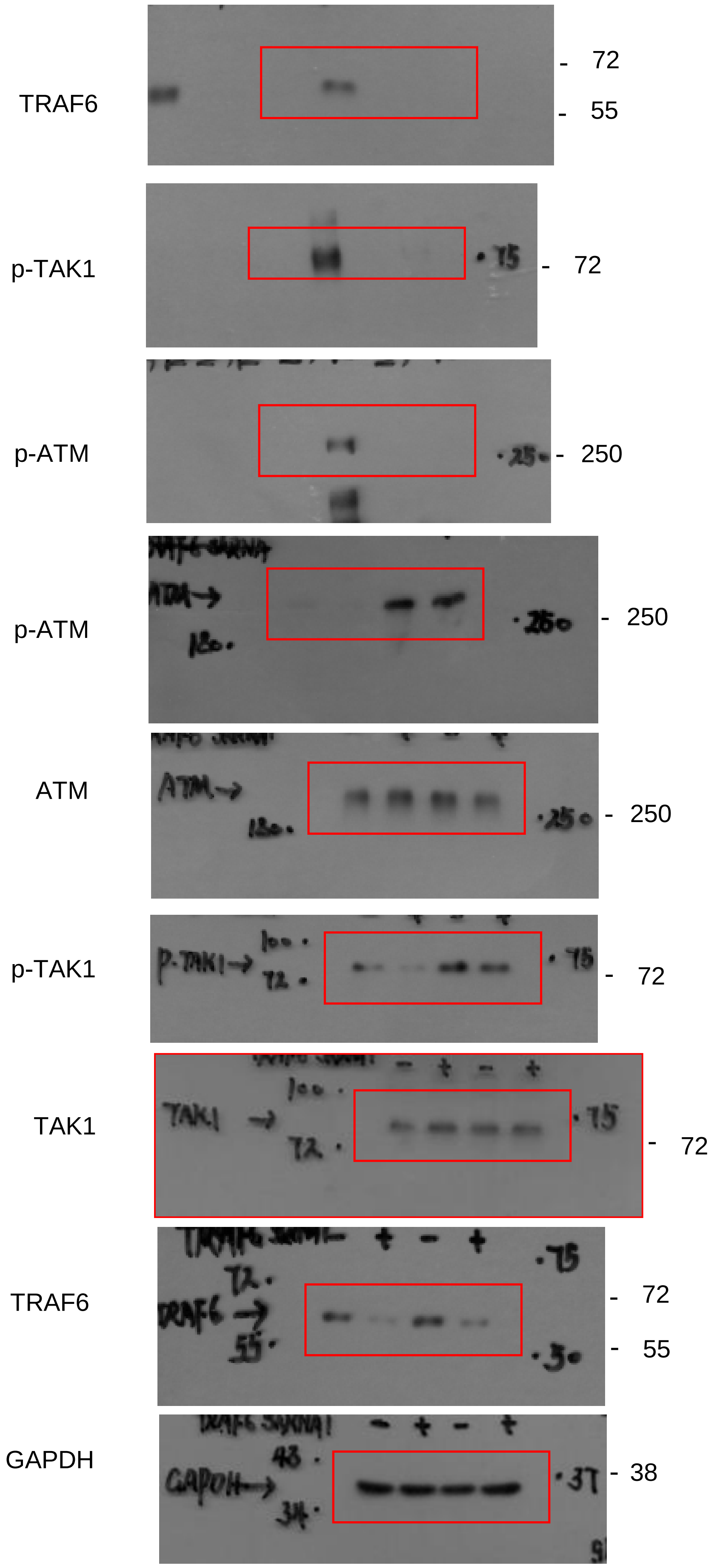

Supplementary Figure 13. The uncropped scans of immunoblots (continued)

Fig. 4e

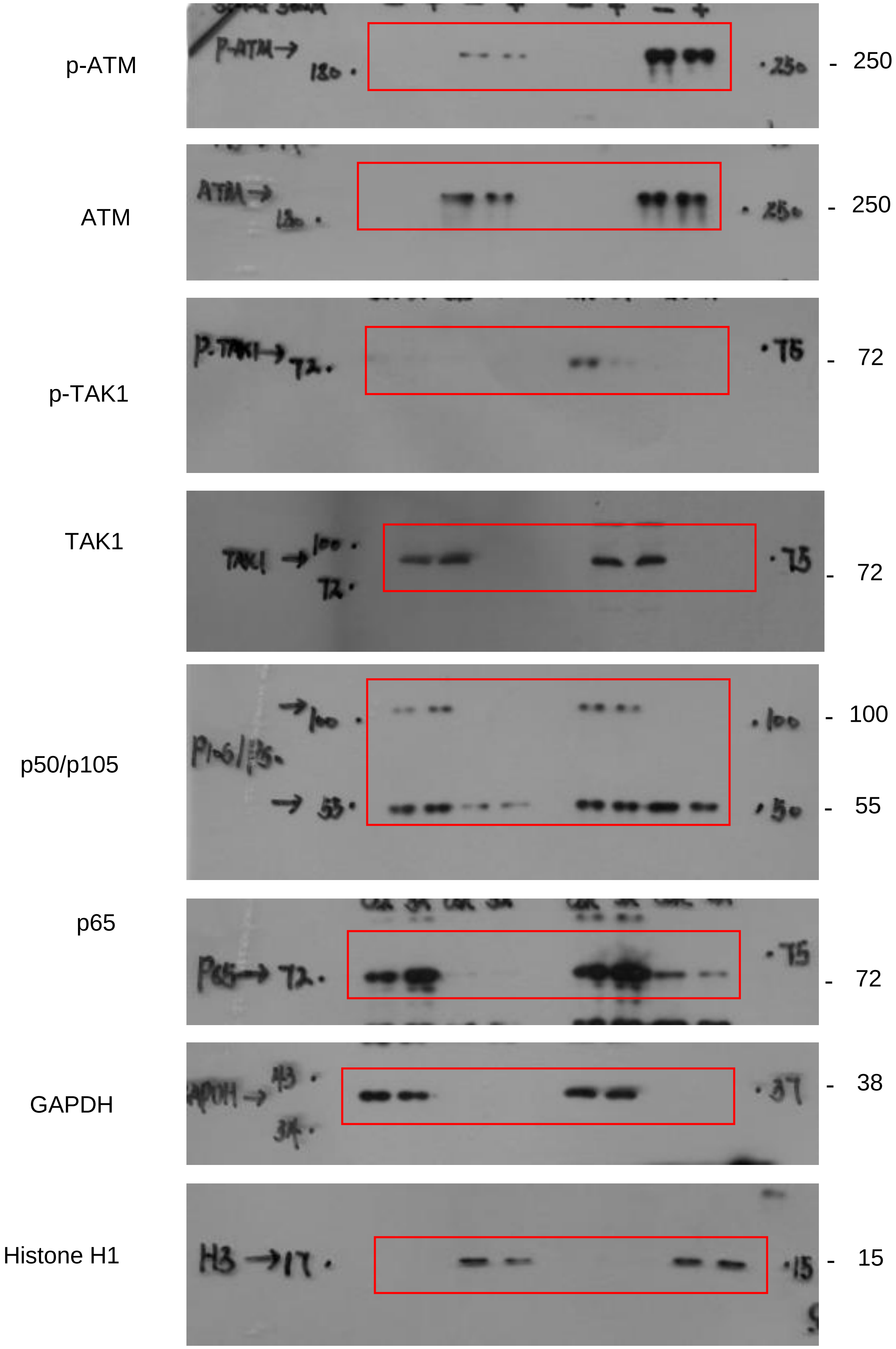

Supplementary Figure 13. The uncropped scans of immunoblots (continued)

Fig. 4h

IL-6 — 38

Timp-1 — 28

Zscan4 — 49

GAPDH — 38

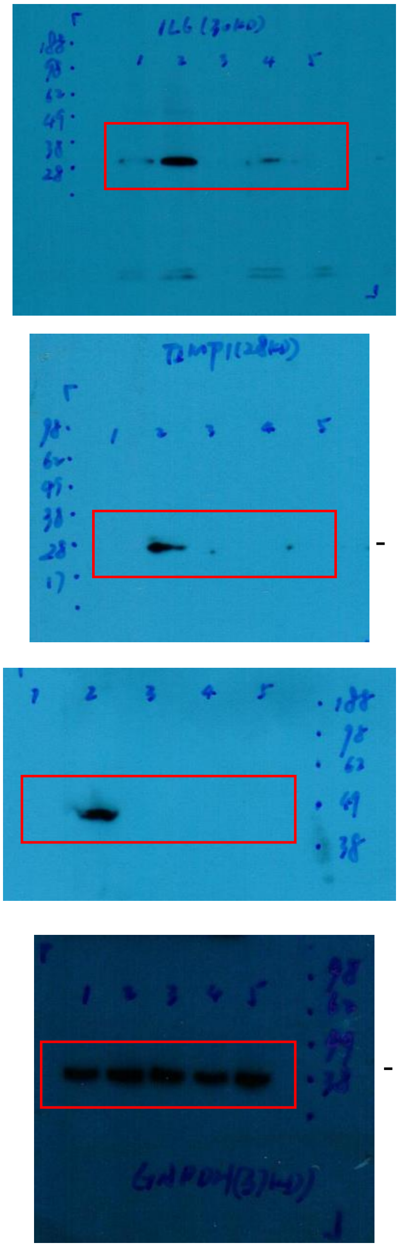

Supplementary Figure 13. The uncropped scans of immunoblots (continued)

Fig. 5a

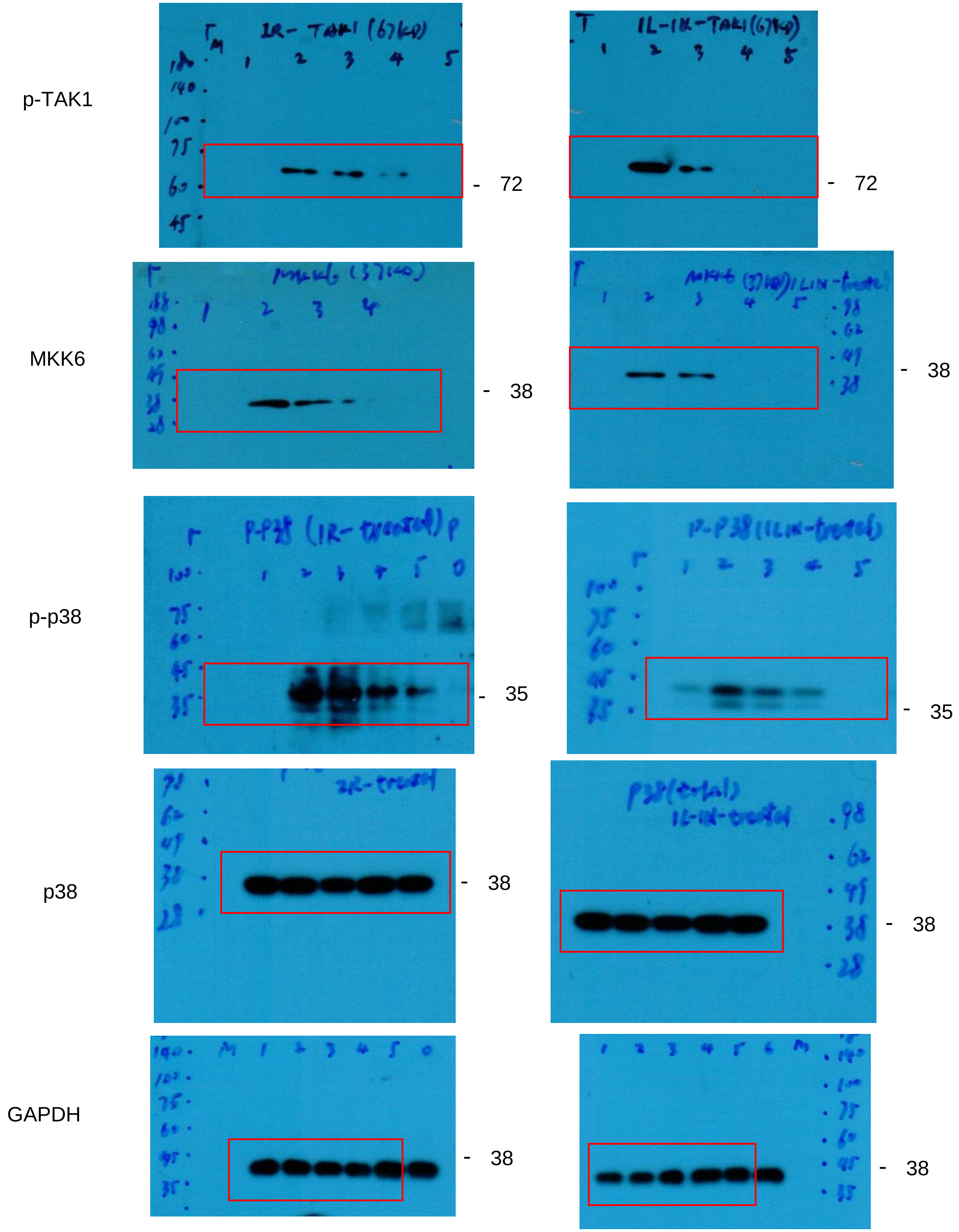

Supplementary Figure 13. The uncropped scans of immunoblots (continued)

Fig. 5b

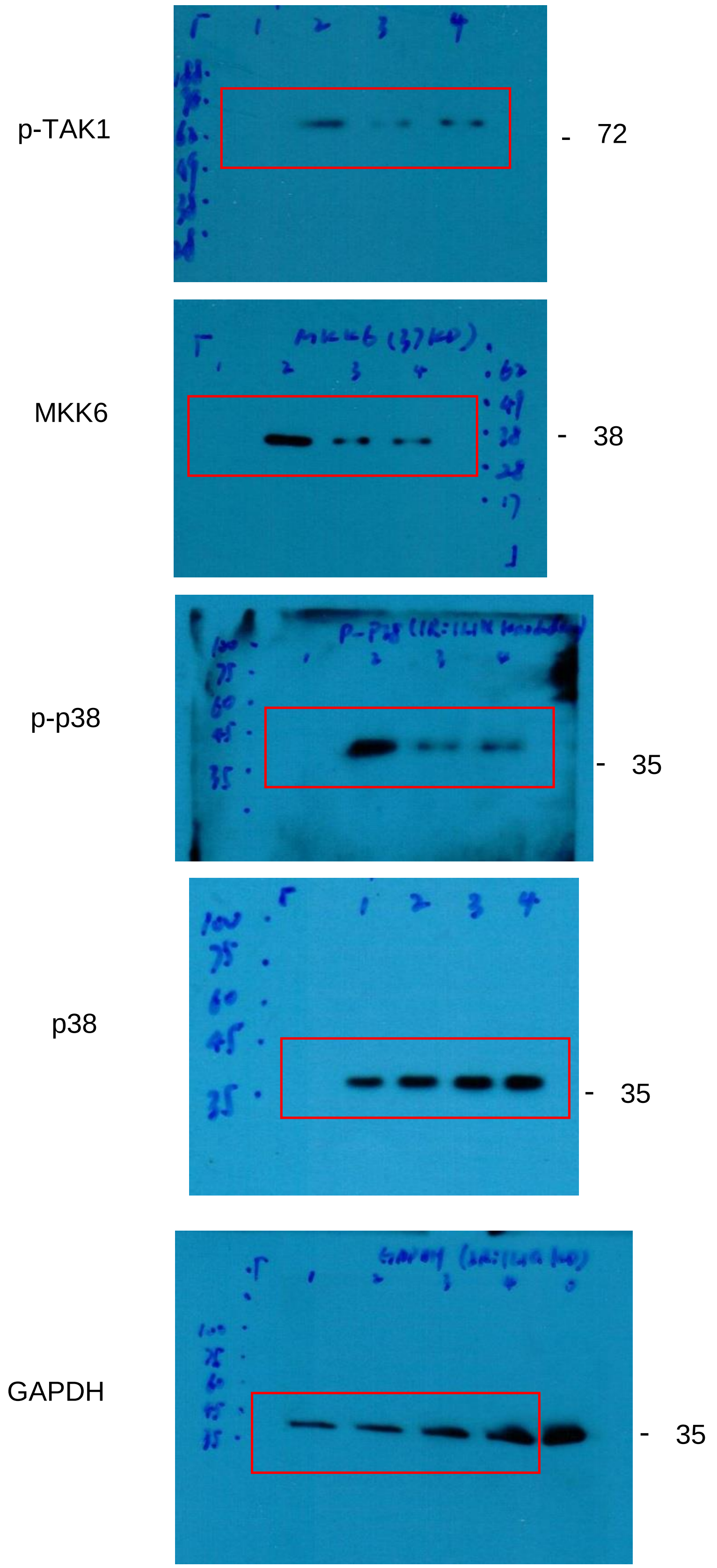

Supplementary Figure 13. The uncropped scans of immunoblots (continued)

Fig. 5e

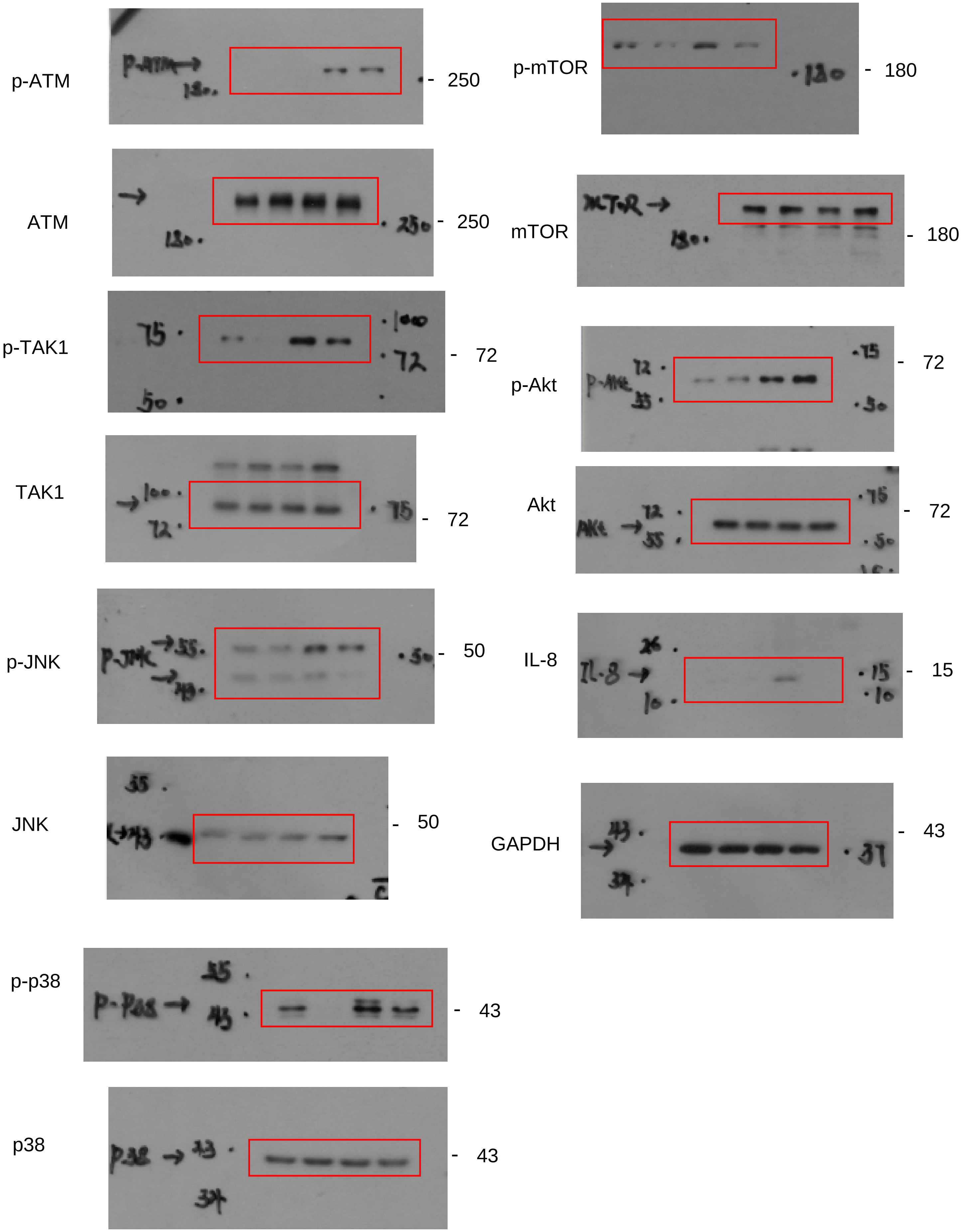

Supplementary Figure 13. The uncropped scans of immunoblots (continued)

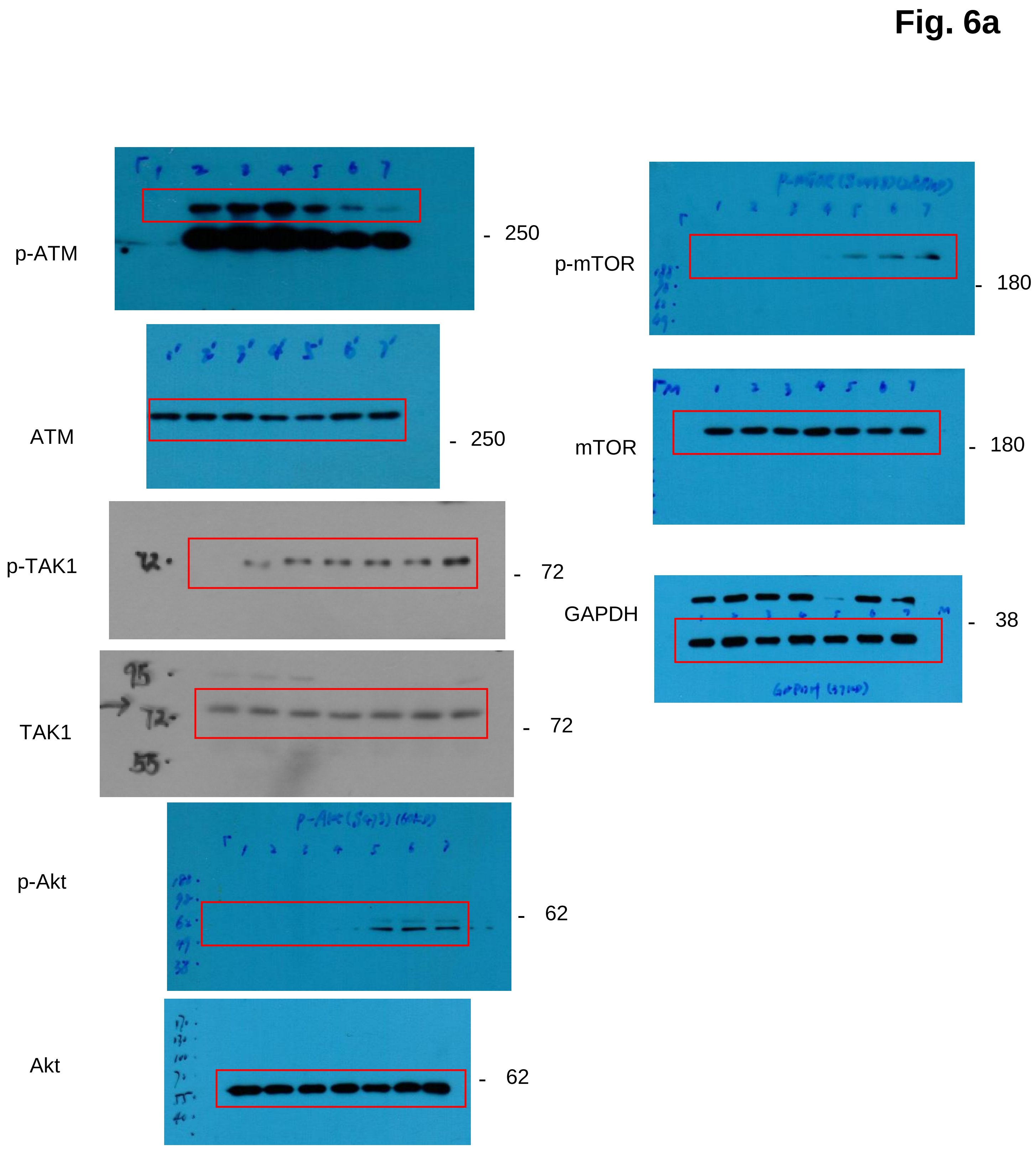

Supplementary Figure 13. The uncropped scans of immunoblots (continued)

Fig. 6c

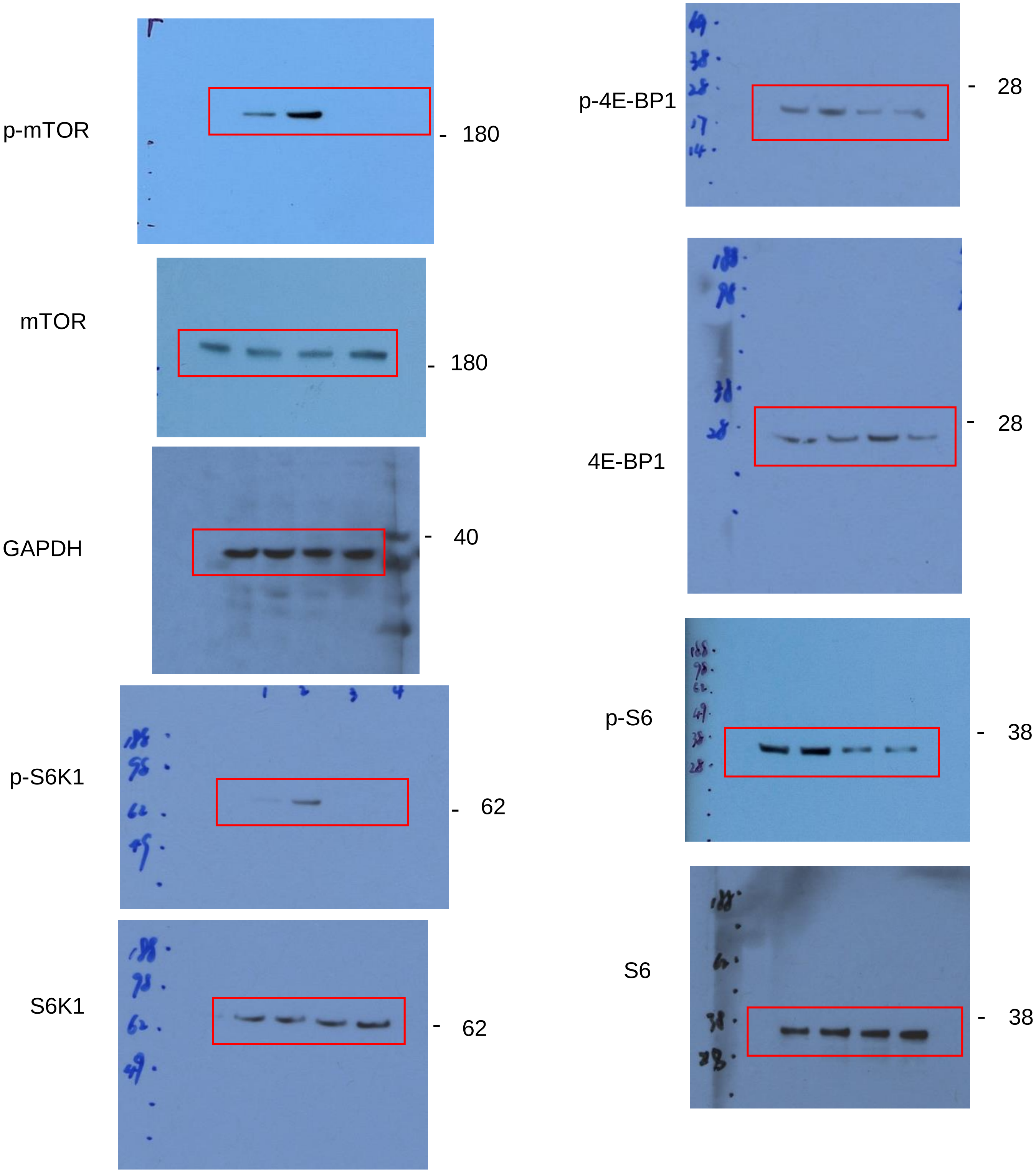

Supplementary Figure 13. The uncropped scans of immunoblots (continued)

Fig. 6f

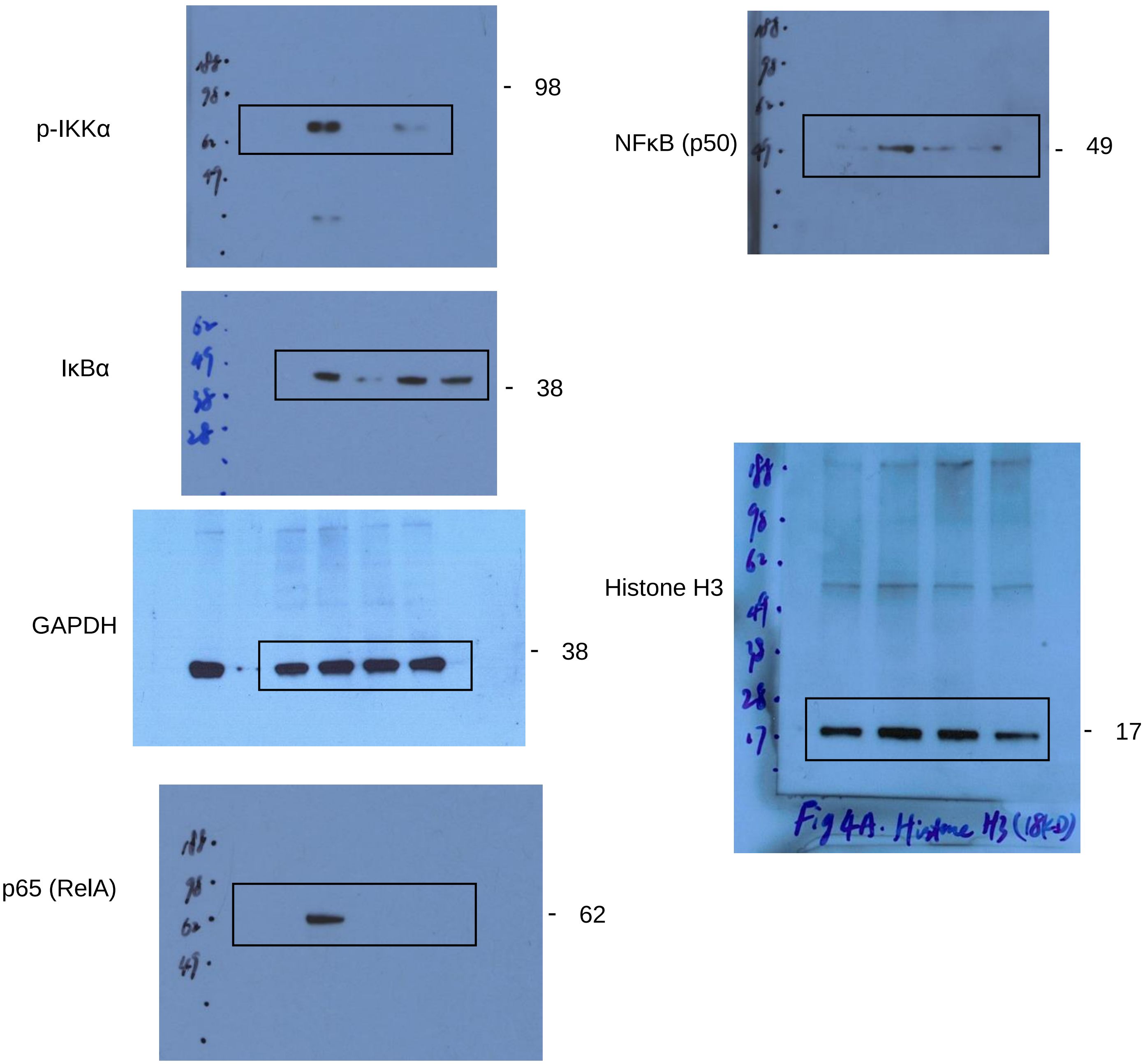

Supplementary Figure 13. The uncropped scans of immunoblots (continued)

Fig. 6h

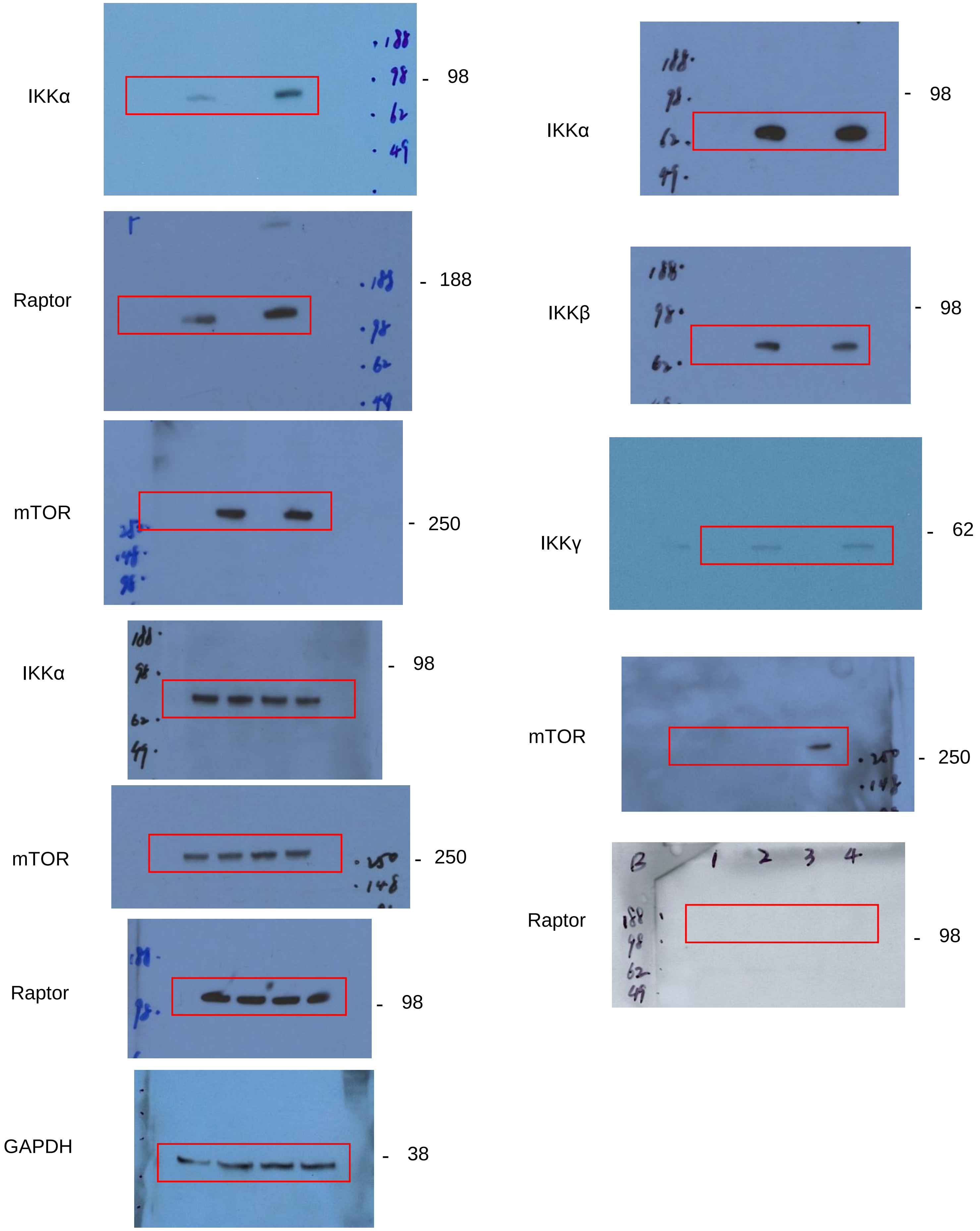

Supplementary Figure 13. The uncropped scans of immunoblots (continued)

Fig. S1h

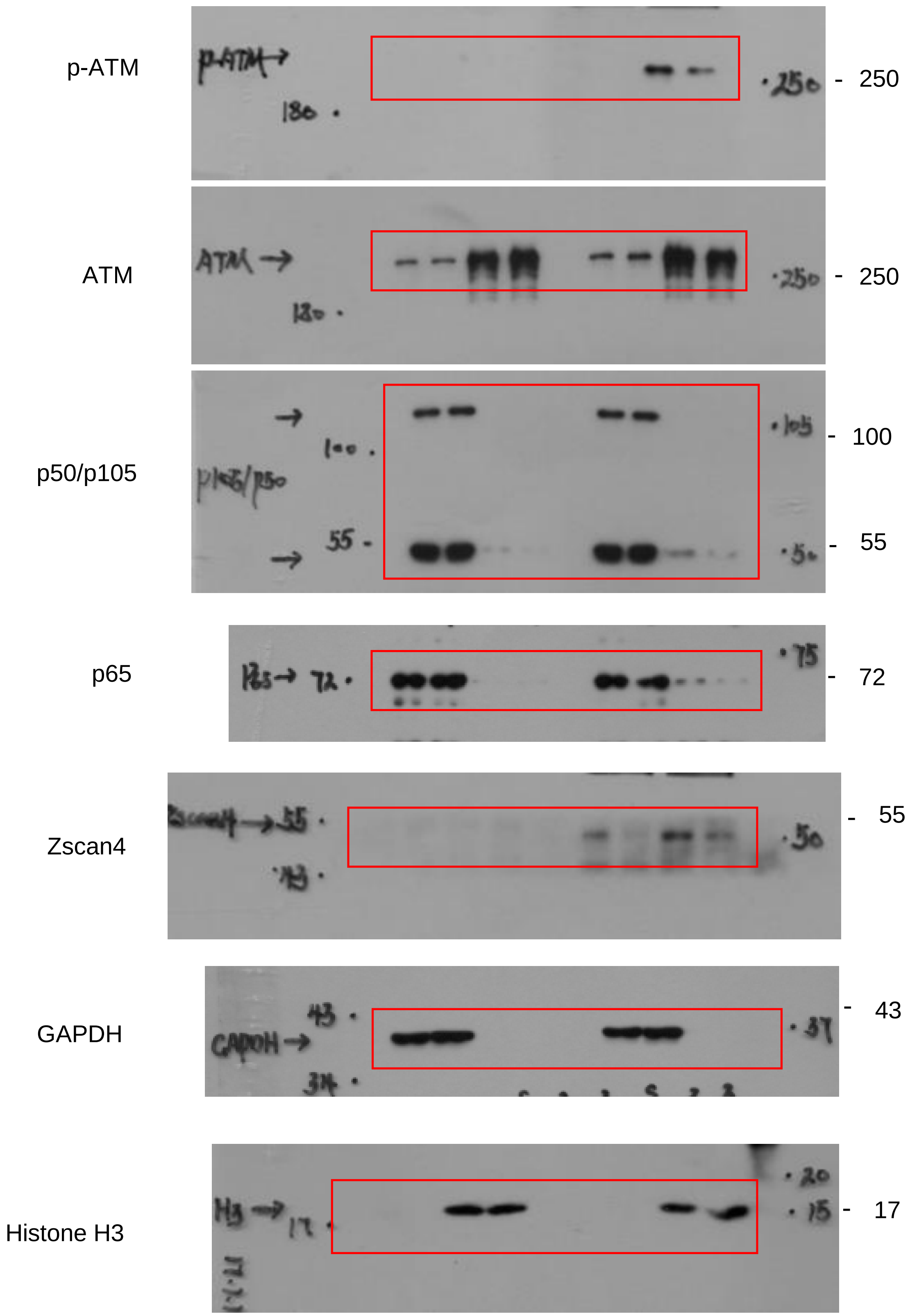

Supplementary Figure 13. The uncropped scans of immunoblots (continued)

Fig. S1i

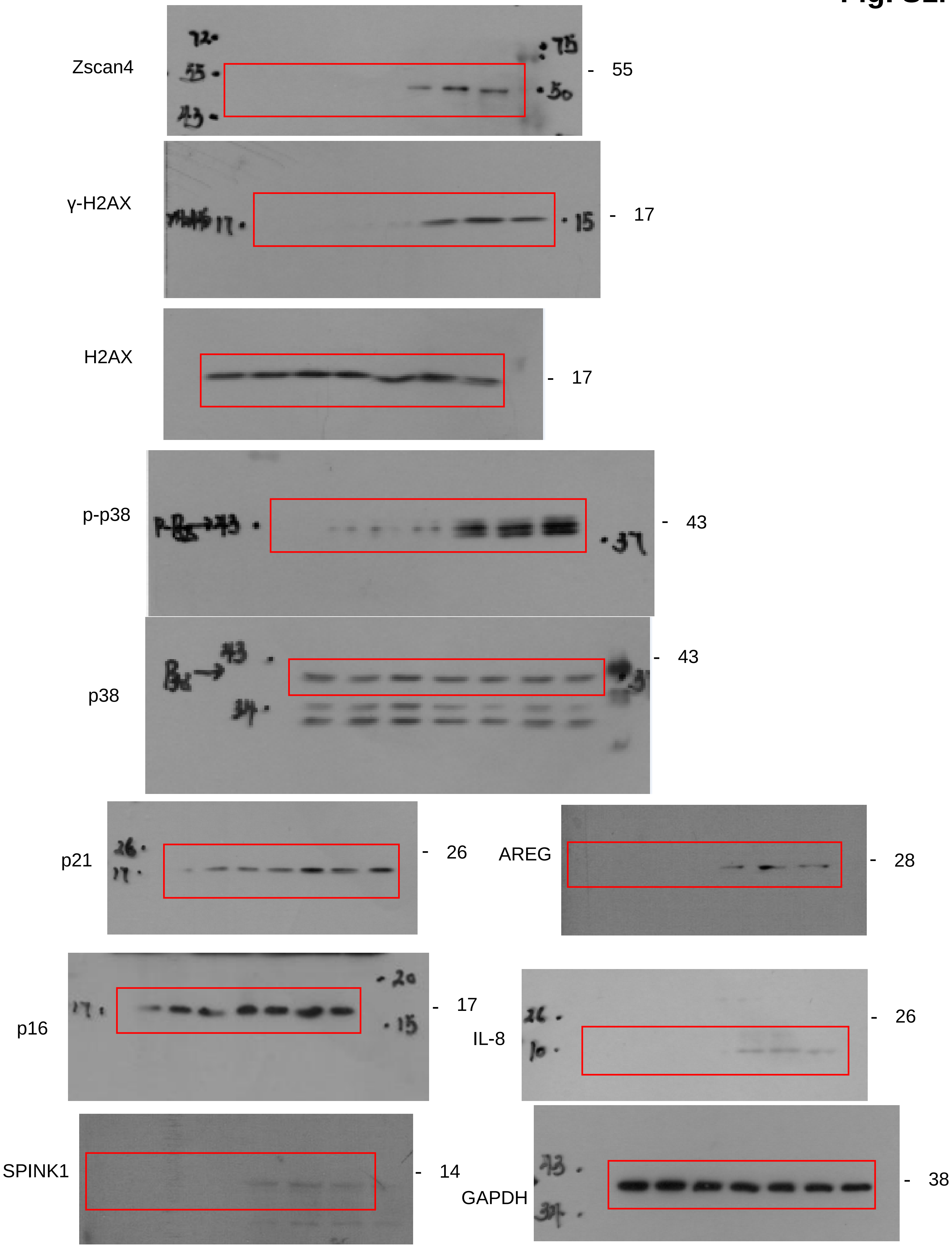

Supplementary Figure 13. The uncropped scans of immunoblots (continued)

Fig. S3g

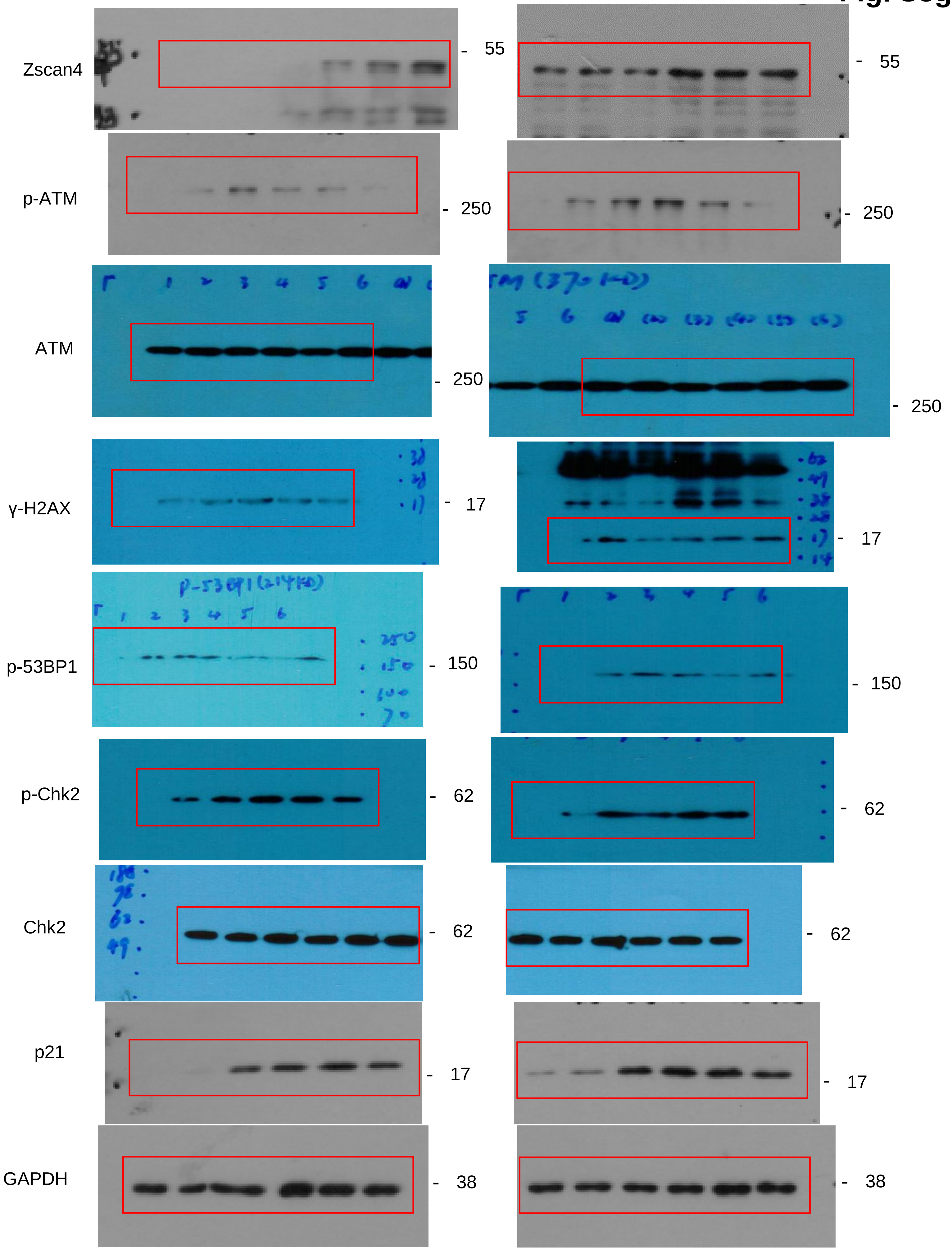

Supplementary Figure 13. The uncropped scans of immunoblots (continued)

Fig. S4d

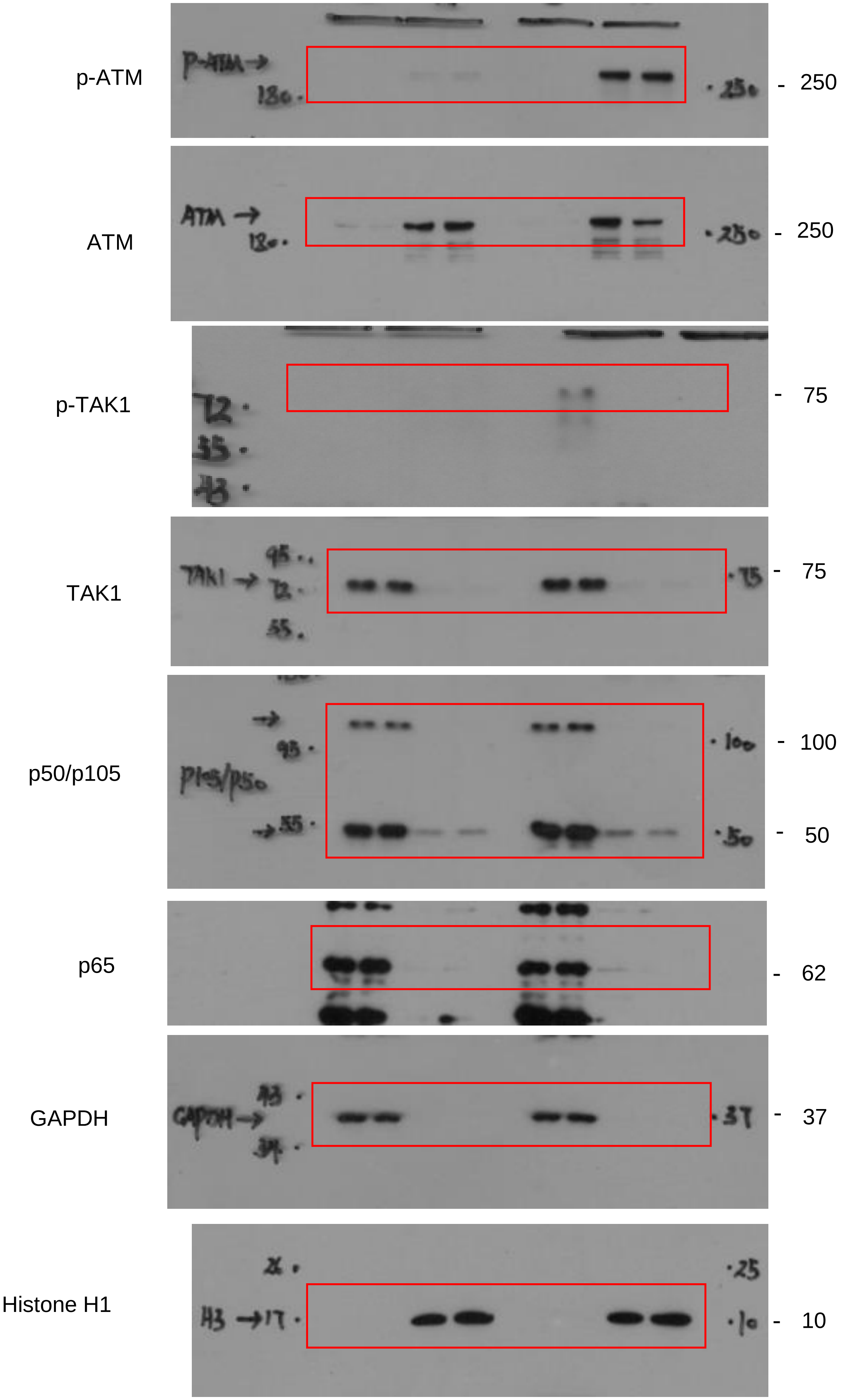

Supplementary Figure 13. The uncropped scans of immunoblots (continued)

Supplementary Table 1 (Primer sequences for qRT-PCR assays)

Target name	Forward (5'-3')	Reverse (5'-3')
**IL-6**	TACCCCCAGGAGAAGATTCC	TTTTCTGCCAGTGCCTCTTT
**IL-8**	GTGCAGTTTTGCCAAGGAGT	CTCTGCACCCAGTTTTCCTT
**TIMP-1**	AATTCCGACCTCGTCATCAG	TGCAGTTTTCCAGCAATGAG
**CSF2**	CCCCAGTCACCTGCTGTTAT	TGGAATCCTGAACCCACTTC
**SPINK1**	CCTTGGCCCTGTTGAGTCTA	GCCCAGATTTTTGAATGAGG
**SFRP2**	GCCTCGATGACCTAGACGAG	GATGCAAAGGTCGTTGTCCT
**AREG**	TGGATTGGACCTCAATGACA	AGCCAGGTATTTGTGGTTCG
**EREG**	CGTGTGGCTCAAGTGTCAAT	TGGAACCGACGACTGTGATA
**ANGPTL4**	GCCTATAGCCTGCAGCTCAC	AGTACTGGCCGTTGAGGTTG
**MMP3**	GCAGTTTGCTCAGCCTATCC	GAGTGTCGGAGTCCAGCTTC
**WNT16B**	GCTCCTGTGCTGTGAAAACA	TGCATTCTCTGCCTTGTGTC
**Zscan4**	CTAGGCCTGAAGAGGGAGGT	CTCCATGGGATCCTTTCTGA
**IL-2**	TGCAACTCCTGTCTTGCATT	GCCTTCTTGGGCATGTAAAA
**IL-12**	GATGGCCCTGTGCCTTAGTA	TCAAGGGAGGATTTTTGTGG
**CXCL1**	AGGGAATTCACCCCAAGAAC	TGGATTTGTCACTGTTCAGCA
**RPL13A**	GTACGCTGTGAAGGCATCAA	CGCTTTTTCTTGTCGTAGGG

Supplementary Table 2 (key resources table)

REAGENT or RESOURCE	SOURCE	IDENTIFIER
**Antibodies**		
ATM (rabbit) (1:1000)	Abways	Cat# CY5207
Phosphor-ATM (rabbit) (1:1000)	Abways	Cat# CY5111
53BP1 (rabbit) (1:1000)	Proteintech	Cat# 20002-1-AP
Phosphor-53BP1 (mouse) (1:1000)	Santa Cruz	Cat# sc-135748
H2AX (rabbit) (1:2000)	Proteintech	Cat# 10856-1-AP
γ-H2AX (rabbit) (1:1000)	Cell signaling	Cat #9718
TRAF6 (rabbit) (1:1000)	Abways	Cat# CY5174
TAK1 (rabbit) (1:1000)	Proteintech	Cat# 12330-2-AP
Phosphor-TAK1 (rabbit) (1:1000)	Abcam	Cat# ab109404
p38 (rabbit) (1:1000)	Proteintech	Cat# 14064-1-AP
Phosphor-p38 (rabbit) (1:2000)	R&D	Cat# AF869
JNK (rabbit) (1:1000)	Proteintech	Cat# 10023-1-AP
Phosphor-JNK (rabbit) (1:1000)	Cell signaling	Cat# 4668
mTOR (rabbit) (1:1000)	Proteintech	Cat# 20657-1-AP
Phosphor-mTOR (rabbit) (S2448) (1:1000)	Cell signaling	Cat# 2971
AKT (rabbit) (1:2000)	Proteintech	Cat# 10176-2-AP
Phosphor-AKT (rabbit) (S473) (1:1000)	Abcam	Cat# ab81283
p105/p50 (rabbit) (1:1000)	Abways	Cat# CY5040
p65 (mouse) (1:1000)	Santa Cruz	Cat# sc-8008
Zscan4 (rabbit) (1:1000)	Abclonal	Cat# A12015
IL-8 (rabbit) (1:1000)	Proteintech	Cat# 60141-2-Ig
E-cadherin (rabbit) (1:2000)	Proteintech	Cat# 20874-1-AP
N-cadherin (rabbit) (1:2000)	Proteintech	Cat# 22018-1-AP
Vimentin (mouset) (1:2000)	Vazyme	Cat# Ab101
GAPDH (mouse) (1:2000)	Vazyme	Cat# Ab103
β-actin (goat) (1:1000)	Santa Cruz	Cat# sc-1615
Histone H1 (rabbit) (1:500)	Proteintech	Cat# 17510-1-AP
Histone H3 (rabbit) (1:1000)	Proteintech	Cat# 17168-1-AP
AREG (goat) (1:500)	R&D	Cat# AF262
IL-1α (mouse) (1:500)	R&D	Cat# MAB200R
IL-7 (goat) (1:400)	R&D	Cat# AF407
IL-8 (mouse) (1:500)	R&D	Cat# MAB208
MKK6 (rabbit) (1:1000)	Cell signaling	Cat# 8550
GST (mouse) (1:500)	Cell signaling	Cat# 2614
Raptor (mouse) (1:500)	Millipore	Cat# 05-1470
SFRP2 (rabbit) (1:400)	Millipore	Cat# 06-004
p70 S6K1 (rabbit) (1:500)	Proteintech	Cat# 14485-1-AP
Phosphor-p70 S6K1 (rabbit) (1:1000)	Millipore	Cat# 07-018
4E-BP1 (mouse) (1:1000)	Santa Cruz	Cat# sc-81149
Phosphor-4E-BP1 (rabbit) (1:500)	Cell signaling	Cat# 13396

S6 ribosomal protein (rabbit) (1:1000)	Cell signaling	Cat# 2217
Phosphor-S6 (rabbit) (1:1000)	Cell signaling	Cat# 4858
IKKα (rabbit) (1:1000)	Cell signaling	Cat# 2682
Phosphor-IKKα (rabbit) (1:500)	Cell signaling	Cat# 2697
IKKβ (mouse) (1:1000)	Novus Biologicals	Cat# NB100-56509
Phosphor-IKKβ (rabbit) (1:200)	Bioss	Cat# bs-3232R
IRAK1 (rabbit) (1:500)	Abcam	Cat# ab5522
IL-24 (goat) (1:400)	R&D	Cat# AF1965
IKBα (rabbit) (1:500)	Abcam	Cat# ab32518
PI3K-p110 (mouse) (1:1000)	Santa Cruz	Cat# sc-8010
PI3K-p85 (rabbit) (1:500)	Epitomics	Cat# 5405-1
SPINK1 (mouse) (1:500)	Abnova	Cat# H00006690-M01
WNT16B (mouse) (1:1000)	BD Biosciences	Cat# 552595
**Chemicals, Peptides, and Recombinant Proteins**		
MK-2206	Selleck Chemicals	S1078; CAS: 1032350-13-2
SB203580	MedChem Express	HY-10256; CAS: 152121-47-6
RAD001	Selleck Chemicals	S1120; CAS: 159351-69-6
5Z-7-Oxozeaenol	TOCRIS	3604; CAS: 66018-38-0
PP242	Selleck Chemicals	S2218; CAS: 1092351-67-1
ATP, [γ-^{32}P]-3000Ci/mmol 10mCi/ml Lead, 5 mCi	Perkin Elmer	NEG002A005MC;CAS: 51963-61-2
KU55933	TOCRIS	3544; CAS: 587871-26-9
BAY 11-7082	Selleck Chemicals	S2913; CAS: 19542-67-7
Bleomycin	Selleck Chemicals	S1214; CAS: 9041-93-4
Mitoxantrone	Selleck Chemicals	S1889; CAS: 65271-80-9
Satraplatin	MedChem Express	HY-17576; CAS: 129580-63-8
Docetaxel	TOCRIS	4056; CAS: 114977-28-5
Paclitaxel	MedChem Express	HY-B0015; CAS: 33069-62-4
Vincristine	Selleck Chemicals	S1241; CAS: 2068-78-2
D-Luciferin	BioVision	7903; CAS: 115144-35-9
Recombinant Human IL-1α	R&D	200-LA
Calf Intestinal Phosphatase	New England Biolabs	M0290S
Normal Goat Serum	Thermo Fisher	01-6201
**Critical Commercial Assays**		
Caspase Glo 3/7	Promega	G8090

Dual-Luciferase® Reporter Assay System	Promega	E1910
Beta-Galactosidase Staining Kit	BioVision	K802-250
BrdU In Vitro Labeling Kit	BD Pharmingen	550891
**Deposited Data**		
Raw and analyzed data	This paper	GEO: GSE82033
Raw and analyzed data	This paper	GEO: GSE90866
**Experimental Models: Cell Lines**		
BPH1	Gift from Prof Simon Hayward	N/A
M12	Gift from Prof Stephen Plymate	N/A
PC3	ATCC	N/A
DU145	ATCC	N/A
LNCaP	ATCC	N/A
PSC27	Gift from Prof Peter Nelson	N/A
**Experimental Models: Organisms/Strains**		
Mouse: ICR SCID	Taconic	ICRSC
**Oligonucleotides**		
shRNA1 targeting sequence:Zscan4: AATCTTGGATCAGAAAATTCA	This paper	N/A
shRNA2 targeting sequence:Zscan4: CAAATAGTTTCCCTAATCATC	This paper	N/A
shRNA3 targeting sequence:Zscan4: CTACGGGTGCATCAGATAATT	This paper	N/A
shRNA1 targeting sequence:TAK1: CCCGTGTGAACCATCCTAATA	This paper	N/A
shRNA2 targeting sequence:TAK1: CGCCCTTCAATGGAGGAAATT	This paper	N/A
shRNA3 targeting sequence:TAK1: CATCCCAATGGCTTATCTTAC	This paper	N/A
shRNA1 targeting sequence:TRAF6: GCCACGGGAAATATGTAATAT	This paper	N/A
shRNA2 targeting sequence:TRAF6: CGGAATTTCCAGGAAACTATT	This paper	N/A

shRNA3 targeting sequence:TRAF6: CCTGGATTCTACACTGGCAAA	This paper	N/A
shRNA1 targeting sequence:IL-1α: GCCAAAGTTCCAGACATGTTT	This paper	N/A
shRNA2 targeting sequence:IL-1α: GAATGACGCCCTCAATCAAAG	This paper	N/A
shRNA3 targeting sequence:IL-1α: GCCAATGATCAGTACCTCACG	This paper	N/A
Scramble shRNA sequence: GATTAATAGGATTGCAGAATT	This paper	N/A
See Table S1 for primers used for SASP qRT-PCR assays	This paper	N/A
See Table S2 for Primers used for Zscan4 promoter cloning and ChIP PCR	This paper	N/A
**Recombinant DNA**		
Plasmid: pLenti-CMV-Zscan4-puro	This paper	N/A
Plasmid: pCMV-Flag-IKKα	This paper	N/A
Plasmid: pCMV-GST-mTOR	This paper	N/A
Plasmid: pCMV-VSV-G	Addgene	8454
Plasmid: pCMV-dR8.2dvpr	Addgene	8455
Plasmid: pLKO.1 puro	Addgene	8453
**Software and Algorithms**		
CellProfiler	Broad Institute	http://www.cellprofiler.org
R version 3.1.2	R Foundation	https://www.r-project.org
PANTHER	Thomas Lab	http://pantherdb.org/

Venn diagram	Bioinformatics & Evolutionary Genomics	http://bioinformatics.psb.ugent.be/webtools/Venn/
Gene Set Enrichment Analysis	Broad Institute	http://software.broadinstitute.org/gsea/index.jsp
NetworkAnalyst	McGill	www.networkanalyst.ca
Agilent GeneSpring GX software (v12.1)	Agilent Technologies	http://www.agilent.com/en-us/products/software-informatics/life-sciences-informatics/genespring-gx
Prism5	GraphPad	www.graphpad.com/scientificsoftware/ prism

Supplementary Table 3
(Primer sequences for Zscan4 promoter cloning and ChIP-PCR assays)

A. Promoter cloning

Amplicon name	Forward	Reverse
Zscan4-P01	CAGTGGCTGTGTGTCTTTGCCTTC	GTGGCAGAGGGCAAGGCTAATGCTG
Zscan4-P02	CTACATACTGAGGCGGATTGGC	GTGGCAGAGGGCAAGGCTAATGCTG
Zscan4-P03	GGGGACCCCAGTCTTTGCGTTCA	GTGGCAGAGGGCAAGGCTAATGCTG
Zscan4-P04	CTTTGCGAGGCCCAAGTAGGTTTAC	GTGGCAGAGGGCAAGGCTAATGCTG
Zscan4-P05	TGCACTCTGCTAATGCAAGCTACAG	GTGGCAGAGGGCAAGGCTAATGCTG
Zscan4-P06	AGCGCCCCCTCCTCATGGAGACCG	GTGGCAGAGGGCAAGGCTAATGCTG

B. ChIP-PCR

Amplicon name	Forward	Reverse
Zscan4-p1	AGCGCCCCCTCCTCATGGAGACCG	CATGAGGACTGCCAGGCTCTCAGA
Zscan4-p2	CACTCCTGTAATCCCAACAC	GTTCAAGCAATTCTCCTGCC
Zscan4-p3	AGCACTACCTGCCTTCCCAA	ATGAGCAGGAGGATTTATCC
WNT16B-p1	CAGGAAAGGTCATGACACACC	AGAGCAGCCTGGGGATCT
SFRP2-p1	ATTCATTACCCGGCTCCTCT	CCTGCCTAGAGATCCACGAG
IL-6-p1	AAATGCCCAACAGAGGTCA	CACGGCTCTAGGCTCTGAAT
IL-8-p1	AAAACTATAGGAGCTACATT	TCGCTTCTGGGCAAGTACA

Supplementary Table 4 (Clinical and histopathological features of NSCLC patients)

ID	CS	TNM	DS	MD (cm)	DFS	DFS time (M)	Z score	T score
151113	IIIa	T2N2M0	1	6.5	0	40	0.5	0.6
151535	Ib	T2aN0M0	1	4	0	41	1.5	1.8
145951	Ib	T2aN0M0	1	4	0	38	1.2	1.5
144996	Ib	T2aN0M0	1	3.2	0	39	1.4	1.2
142402	Ib	T2N0M0	3	1.6	0	60	1.0	1.4
140984	Ib	T2aN0M0	1	2.5	0	64	1.0	1.5
122657	IIIa	T2N2M0	1	8	1	43	3.5	3.8
120144	IV	T2N2M1	1	3.6	0	59	0.5	0.7
120415	IV	T4N0M1	1	1.7	1	32	2.5	3.0
155010	Ib	T2N0M0	3	2	0	74	1.1	1.4
136492	IIb	T3N0M0	3	5.5	0	93	0.2	0.4
145779	Ia	T1bN0M0	2	2	0	52	0.7	0.7
143105	Ib	T2aN0M0	2	2.5	0	53	1.2	1.0
121724	Ib	T2N0M0	3	3	0	59	0.4	0.5
136189	IV	T2aN0M1	3	3	0	47	0.5	0.9
146462	Ib	T2N0M0	1	0.8	0	62	1.8	1.9
132109	IV	T2N1M1a	NA	2	0	82	1.1	1.5
136740	IIIa	T2aN1M0	1	1.5	0	63	0.2	0.5
135413	Ib	T2N0M0	1	1.5	1	47	1.5	1.5
112881	IIIb	T4N0M0	1	2.5	1	40	1.8	1.5
110410	IIb	T2N1M0	NA	2.5	1	33	2.5	2.9
103911	IIIb	T4N0M0	NA	2	0	92	0.7	0.3
111506	IV	T2N1M1	2	1.7	0	109	0.5	0.8
121792	Ib	T2N0M0	2	1	1	63	3	3.5
123353	IIIa	T4N0M0	1	2.2	0	62	0.2	0.2
134769	IIIa	T2aN2M0	1	4	0	40	0.8	1
120667	IIb	T2N1M0	2	1.5	0	61	0.9	1.2
113755 A4	IIIa	T3N2M0	1	NA	0	63	1.1	1
114273 A1	IIIb	T4N2M0	1	NA	0	65	0.4	0
114390 A4	IV	T3N2M1	2	3	0	62	1.4	1.8
114449 A1	IIIa	T2N2M0	2	1	1	62	3.6	4
121504 A1	IIIa	T2N2M0	1	9	1	56	2.8	2.5
121749 A2	IIa	T3N0M0	2	2.5	0	55	0.7	0.9
122847 A4	IIa	T3N0M0	NA	5	0	54	0.9	0.5
122901 A1	IIIa	T2bN2M0	NA	5	0	57	1	1.5
123284 A1	IIIa	T2N2M0	NA	3	0	53	0.9	1.2
124141 A4	IIIa	T2bN2M0	1	NA	0	51	0.5	0.9
124450 A2	Ib	T2aN0M0	NA	5	0	52	0.9	1.2
126255 A3	IIIa	T2N2M0	2	2.5	0	48	1.4	1.6
130647 A2	IIIb	T4N2M0	1	5	0	48	0.7	0.9
130681 A0	IIIb	T4N2M0	NA	0.3	0	47	0.8	1
130819 B0	Ia	T1aN0M0	NA	1.1	0	46	0.9	1.1
130961 A1	IIIa	T2N2M0	NA	0.3	0	57	1.2	1.5
132330 B0	Ia	T1aN0M0	NA	2.8	0	44	1.1	0.7
132557 A1	IIIa	T2N2M0	NA	NA	0	43	0.8	0.8
132683 A2	IIIa	T4N0M0	NA	NA	0	44	0.6	1.2
133582 A4	IIIa	T2bN2M0	1	2	0	42	0.9	0.6
134675 A3	Ib	T2aN2M0	1	3.5	0	43	0.6	0.6
146648 A4	Ib	T2aN2M0	2	3	0	27	0.5	1
147707 A3	IIIa	T2N2M0	1	4	0	28	1.2	1.5
148025 A2	IIIb	T4N2M0	2	3.5	1	21	3.2	3
149209 A2	IIIb	T4N2M0	2	1.3	0	27	0.5	0.5
150130 B1	IIIa	T2aN2M0	2	2.5	0	24	0.3	0
150449 A5	IIIa	T4N1M0	2	4	0	23	1.3	1.5

151691 A1	IIIa	T4N0M0	2	1	0	21	1	1.8
120706	IIIa	T4N0M0	2	0.8	0	80	0.5	0.8
072521	IIIa	T4N0M0	NA	NA	0	108	2.9	2.5
072915	IIIa	T4N0M0	NA	NA	0	108	3.5	3.8
130960	Ib	T2aN0M0	2	1.2	1	19	3.5	3.8
137395	IIIa	T4N1M0	2	1.5	1	60	4	3.9
134232-2	IIa	T2bN0M0	2	7	1	63	2.5	2.2
125687-1	IIIa	T2N2M0	1	4	0	61	1.2	1.6
115002-2	IIIa	T2aN1M0	1	4	0	113	2.4	2.8
131153-1	Ib	T2N0M0	2	2	0	53	0.6	0.4
134506-1	Ia	T1N0M0	1	2	1	33	2.5	2.8
136015	IIIa	T2bN2M0	1	6	0	45	1.2	1.5
140528	Ib	T2N0M0	1	6	0	52	1.1	1.7
093006	Ia	T1N0M0	2	6.5	1	97	2.9	2.4
112665-1	Ib	T2aN0M0	2	6	0	103	1.4	1.8
132836-2	Ib	T2N0M0	2	2.5	0	63	1.5	1.5
091198-2	Ib	T2N0M0	2	1.5	0	89	1.6	1.9
114810	IV	T4N1M1	1	7	0	72	3.4	3.8
092480	Ib	T2N0M0	NA	4	1	94	2.5	1.5
147810	Ib	T2N0M0	2	2	0	60	0.6	1
115081-2	IIIa	T2N2M0	2	5	1	79	2.6	2.2
090003-2	IIIa	T2N2M0	NA	1	1	95	2.8	2.9
141729	Ia	T1bN0M0	2	4.5	1	43	3	2.5
142221	Ib	T2N0M0	2	4.5	1	64	3.2	3.8
137866	Ib	T2N0M0	2	3	0	61	1.7	1.9
111253-2	IIIa	T2N2M0	2	3.5	1	7	2.3	2.8
144139	IIIa	T2bN2M0	2	6	0	38	1.3	1.7
148510	IIa	T2bN0M0	1	5	0	32	2.8	2.9
113814-1	IIb	T3N0M0	2	4	0	74	3.8	3.3
101954	Ib	T2N0M0	2	4.5	0	109	1.7	1.9
112286-1	Ia	T1N0M0	2	3.2	0	63	1	0.6
123646-2	Ia	T1N0M0	1	4	0	62	0.3	0.3
114106	IIIA	T4N0M0	2	8	0	83	0.8	1
114349-3	Ia	T1aN0M0	2	1.7	0	65	0.5	0.5
131185	IIIA	T4N0M0	2	2	1	45	2.8	2.2
131320-2	Ia	T1aN0M0	2	NA	0	93	1.4	1.8
124304-1	IIIA	T4N0M0	2	2	0	74	2.2	2.2
100430	IV	T3N2M1	1	1	0	80	0.8	0.4
093202	Ia	T1N0M0	2	2	0	73	0	0.8
112315	Ia	T1N0M0	2	1.5	1	30	3.5	3.5
137782	IIB	T3N0M0	1	5.5	1	34	3.6	3.2
122540	Ia	T1N0M0	2	1.5	0	46	0.6	1
090594	Ib	T2N0M0	2	2.5	1	40	0.8	2.2
091382	Ib	T2N0M0	2	5	1	66	1	0.7
090710	Ia	T1N0M0	2	2.5	1	75	1.5	1.9

Abbreviations:
ID, pathological ID number;
CS, clinical stage;
TNM, TNM stage;
DS, differentiation stage;
MD, maximal diameter;
DFS, disease free survival status (recurrent/mets/death, 1; no disease, 0);
Z score, Zscan4 score;
T score, TAK1 score.
(Both Z and T scores were averaged from 3 independent blind readings per IHC-stained sample for pathological assessment)

Supplementary Table 5 (Clinical and histopathological features of BCa patients)

ID	CS	TNM	MD	ER	PR	ErbB2	Ki67	DFS	DFS time (M)	Z score	T score
153402-17	IIa	T2N0M0	0	-	-	+	+	NA	40	0.8	0.5
152935-13	IIb	T2N1M0	0	-	-	+	+	1	32	1.4	2.9
161894-16	IIa	T2N0M0	0	-	-	+	+	NA	41	1.2	1.8
158061-9	IIa	T2N0M0	0	+	-	+	+	NA	34	1	0.5
158060-14	IIa	T2N0M0	0	+	-	+	+	NA	28	0	0.7
187928-15	I	T1N0M0	0	++	-	++	+	NA	14	0.5	1
180393-22	IIIa	T1N2M0	0	-	-	-	+	1	22	2.9	2.2
190855-11	IIa	T1N1M0	0	++	+	-	+	1	22	1.5	1.9
166497-34	IIa	T2N1M0	0	+	-	-	+	1	12	2.7	2.0
178259-14	IIa	T1N1M0	0	-	-	+++	+	1	17	1.0	1.6
162598-16	IIIa	T1N2M0	0	-	-	++	+	1	14	2.5	2.5
183638-14	IIa	T2N0M0	0	+	+	+++	+	NA	25	0.5	0.9
187862-15	IIb	T2N1M0	0	+	-	-	+	1	14	3.5	3.8
190220-35	IIa	T2N0M0	0	+++	-	-	+	NA	20	3.0	3.5
182175-13	IIa	T2N0M0	0		-	+++	+	NA	33	2.2	2.1
184543-11	IIa	T2N0M0	0	-	+	+++	+	NA	14	1.2	1.2
179751-16	IIa	T1N1M0	0	+	-	-	+	1	24	1.1	1.8
180720-9	IIa	T1N1M0	0	+	+	+	+	1	22	3.0	3.8
180718-18	IIb	T2N1M0	0	-	-	+	+	1	23	2.3	2.8
184776-28	IIIa	T1N2M0	0	+	-	-	+	1	25	3.6	3.1
180714-13	IIb	T3N0M0	1	-	-	+++	+	NA	18	1.2	1.8
179955-14	IIa	T2N0M0	0	-	-	+++	+	NA	29	0	0.5
183244-14	IIb	T2N1M0	0	-	-	+++	+	1	25	2.4	2.9
184023-16	IIIa	T1N2M0	0	++	+	-	+	1	25	3.8	3.1
194682-15	IIb	T2N1M0	0	+		+++	+	1	17	1.5	1.0
97769	IIIa	T3N2M0	1	-	-	+++	+	1	28	2.5	2.9
113871	IIb	T2N1M0	0	-	-	+++	+	1	7	1.5	1.8
112048	IIIb	T4N2M0	0	+++	+++	+++	+	1	9	2.5	2.1
112009-2	IIIa	T3N2M0	1	+++	++	+	+	1	9	2.8	2.2
112523	IIIb	T4N2M0	0	+++	-	-	+	1	9	3.6	3.9
113137	IIIa	T3N2M0	1	-	-	+++	++	1	8	2.5	2.2
113389	IIIc	T4N3M0	0	+++	-	++	+	1	8	3.5	3.9
113432	IIa	T1N1M0	0	+++	+++	-	+	1	8	1.6	1.0
113515	IIIc	T3N3M0	1	+++	+++	++	+	1	8	3.5	3.9
113668	I	T1N0M0	0	-	-	-	+	1	13	1.2	2.8
114304	I	T1N0M0	0	-	-	+++	+	NA	13	1.2	1.2
114256	IIIa	T2N2M0	0	-	-	+++	+++	1	7	2.5	2.0
114083	IIa	T2N0M0	0	-	-	-	++	NA	12	1.2	1.6
113020-3	IIIb	T4N2M0	0	-	-	++	++	1	8	2.5	2.1
114160	IIIb	T4N1M0	0	+++	++	+	+	1	7	3.0	3.5
106548	IIIa	T1N2M0	0	+	-	-	+	1	16	3.8	3.2
107125	IIIa	T2N2M0	1	+++	+++	-	++	1	15	1.5	1.9
108766	IIb	T2N1M0	0	+++	++	++	+	1	13	1.8	1.8
108862	IIIb	T4N1M0	1	+++	++	-	+	1	13	3.2	3.0
109377-13	IIb	T2N1M0	0	-	-	-	+	1	12	2.4	2.9
102122	IIIa	T1N2M0	0	+++	+	+	+	1	10	3.0	3.7
103564	IIIa	T2N2M0	0	+++	++	++	+	1	8	2.5	2.1
103781	I	T1N0M0	0	++	-	+++	+	NA	26	0.5	2.5
103853	I	T1N0M0	0	-	-	+	++	NA	25	0.2	0.8
104419	IIa	T2N0M0	0	-	-	+++	++	NA	25	0	0.5
109994	IIIc	T3N3M0	1	++	+	++	+++	1	12	3.5	3.8
110138	IIb	T2N1M0	0	+++	+	++	++	1	13	1.5	1.2

110292	IIa	T2N0M0	0	+	-	-	+	NA	13	2.8	2.8
110780	IIb	T3N0M0	1	+++	+++	++	++	NA	17	3.0	3.5
99559	IIIa	T1N2M0	0	+++	++	+	++	1	25	2.5	2.5
97922	IIb	T2N1M0	0	+	-	-	+++	1	28	2.7	2.2
100430	IIa	T1N1M0	0	-	-	++	+++	1	24	4.0	3.6
101372	IIa	T1N1M0	0	++	+++	++	+	1	23	1.5	1.1
111628	IIIa	T2N2M0	0	+++	-	++	+	1	10	1.2	1.8
110929	IIIa	T3N2M0	1	+++	+++	-	+	1	11	2.9	3.2
110875	I	T1N0M0	0	-	-	-	+	NA	17	1.2	1.8
110291	IIIb	T4N2M0	0	++	+++	-	+	1	11	4.0	3.8

Abbreviations:
ID, pathological ID number;
CS, clinical stage;
TNM, TNM stage;
MD, maximal diameter (<5.0 cm, 0;⩾5.0 cm, 1);
DFS, disease free survival status;
ER, estrogen receptor;
PR, progesterone receptor;
ErbB2, Her2 receptor;
Z score, Zscan4 score;
T score, TAK1 score.
(Both Z and T scores were averaged from 3 independent blind readings per IHC-stained sample for pathological assessment)
